# Bifunctional Double-Salt Ionic Liquids Containing both 4-Chloro-2-Methylphenoxyacetate and l-Tryptophanate Anions with Herbicidal and Antimicrobial Activity

**DOI:** 10.1021/acsomega.1c05048

**Published:** 2021-12-03

**Authors:** Daria Szymaniak, Kamil Ciarka, Katarzyna Marcinkowska, Tadeusz Praczyk, Daniela Gwiazdowska, Katarzyna Marchwińska, Filip Walkiewicz, Juliusz Pernak

**Affiliations:** † Faculty of Chemical Technology, Institute of Chemical Technology and Engineering, Poznan University of Technology, ul. Berdychowo 4, Poznań 60-965, Poland; ‡ PPC ADOB, ul. Kołodzieja 11, Poznań 61-070, Poland; § Institute of Plant Protection, National Research Institute, ul. Węgorka 20, Poznań 60-318, Poland; ∥ Department of Natural Science and Quality Assurance, Institute of Quality Science, Poznan University of Economics and Business, al. Niepodległości 10, Poznań 61-875, Poland

## Abstract

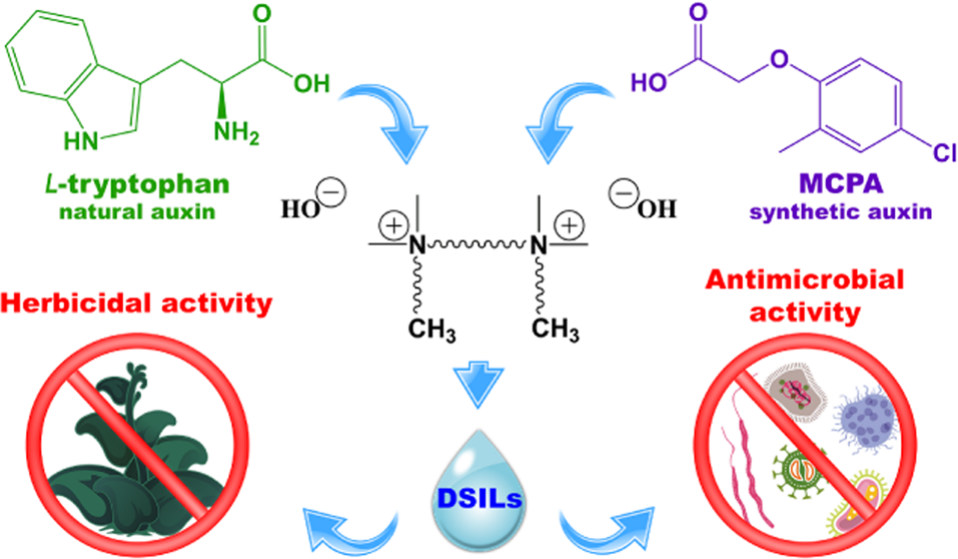

The goal of this research was to obtain and characterize ionic liquids based on a bisammonium cation and both 4-chloro-2-methylphenoxyacetate (MCPA) and l-tryptophanate anions. The concept of including two structurally different anions was utilized to achieve improved biological activity, while crucial functional traits could be designed by modifying the cation. The synthesis process was efficient and resulted in high yields. Subsequent analyses (nuclear magnetic resonance (NMR), Fourier transform infrared (FT-IR) spectroscopy, and high-performance liquid chromatography (HPLC)) confirmed the chemical structure, purity, and molar ratio of ions in the obtained compounds. The described compounds are novel and have not been previously described in the literature. Evaluations of physicochemical properties indicated that the obtained double-salt ionic liquids (DSILs) exhibited high thermal stability, high solubility in water, and surface activity. A biological activity assessment using greenhouse tests revealed that the herbicidal efficiency of the studied DSILs was notably increased compared to the reference commercial herbicide (even by ∼50% in the case of oilseed rape), which could be attributed to their high wettability toward hydrophobic surfaces. The compounds also efficiently inhibited the growth of several microbial species, with minimum inhibitory concentration (MIC) and minimum bactericidal concentration (MBC)/minimum fungicidal concentration (MFC) values at the level of several μg·mL^–1^. The length of the spacer and alkyl substituent in the cation notably influenced the physicochemical and biological properties of the DSILs, which allowed us to design the structures of the obtained compounds in accordance with needs. The presented results confirm the high application potential of the described DSILs and provide a new and promising path for obtaining new and efficient plant-protection agents.

## 1 Introduction

Auxins are phytohormones that control many aspects of plant growth and development. Auxins play a leading role in the mechanisms of cell division, growth, development, and aging of fruit, shoots, and roots. Indole acetic acid (IAA) is the main natural auxin.^1−3^ IAA can be produced by organisms *via* Trp-dependent and Trp-independent mechanisms.^4^ Trp-dependent IAA biosynthesis pathways use tryptophan as a precursor,^5^ which is converted into indolylpyruvate (IPA) by TAA transaminase and then into IAA by flavin monooxygenase.^6^

Synthetic auxins, which were designed and obtained based on the structures and functions of natural auxins, are widely used in agrochemistry. They are used in agriculture not only as growth stimulants but also as herbicides.^7^ Weeds are a major problem in crop plant production, as they can reduce crop yields by 34%.^8^ Therefore, research regarding new herbicides is needed and has been one of the most common research topics in the Weed Science Society of America (WSSA) in recent years. The WSSA distinguishes 35 classes of herbicides, and WSSA Group 4 comprises agents that behave like natural auxins.^9^ They mimic the action of IAA by binding to receptors. Examples of synthetic auxins with herbicidal activity include 1-naphthylacetic acid (1-NAA), 2,3,6-trichlorobenzoic acid (TBA), 2,4-dichloro-phenoxyacetic acid (2,4-D), 4-chloro-2-methylphenoxy acetic acid (MCPA), 4-(4-chloro-2-methylphenoxy)butanoic acid (MCPB), and 4-(2,4-dichlorophenoxy)butyric acid (2,4-DB). Their direct impacts on yields and the signaling pathways and resistance mechanisms in weeds are under investigation. Since the invention of 2,4-D in 1945, 41 types of weeds have developed resistance to this herbicide.^10,11^

Ionic liquids (ILs) are compounds that consist only of ions, and their melting point does not exceed 100 °C. Originally, they were produced to replace volatile solvents. In addition, they are used in the synthesis, catalysis, and electrochemical and chemical analysis of bioactive compounds. They can serve, *inter alia*, as antistatic or dispersing agents as well as adjuvants. Due to the multitude of cation–anion combinations, it is possible to design compounds with virtually any characteristics.^12−14^ The conversion of biologically active compounds into ILs expands the spectrum of their applications. Examples include herbicides, fungicides, feeding deterrents, and growth regulators.^15^ ILs containing anions with a regulatory effect have also been described in the literature. The conversion of 2-chloroethyltrimethylammonium chloride into an IL results in a compound with retained bioactivity that exhibits unique properties depending on the anion included.^16^ Ammonium salts containing the IBA anion and an amino acid cation show regulatory properties toward germination and shoot and root development.^17^ A convenient method of obtaining ammonium salts with an l-tryptophanate anion, which shows plant growth regulation activity and improved physicochemical properties, was described in previous reports.^18,19^ Herbicidal ionic liquids (HILs) are a new approach to known bioactive compounds. The latest reviews outline the broad scope of this research topic, which provides numerous opportunities that still lie ahead of researchers in this field.^20,21^ HILs that incorporate many herbicidal anions have been investigated, including MCPA,^22^ 2,4-D,^23^ dicamba,^24^ 2,4-DP,^25^ nicosulfuron,^26^ and a nonselective pelargonate of natural origin.^27^ Fungicidal ionic liquids are mainly based on compounds containing a triazole group.^28^ Examples of such cations include tebuconazole and propioconazole derivatives.^29^ It is possible to convert fungicidal diols, e.g., dibenzothiophene-5,5-dione, into ILs by replacing the hydroxyl groups with quaternary ammonium groups. Thus, the obtained dichloride retains its fungicidal character.^30^ The combination of two fungicidal cations, thiabendaziol and imazalil, with a single anion allows improved properties to be achieved due to the synergistic effect of bioactive ions complemented by the physicochemical properties of the anion.^31^ Quaternary ammonium salts that combine herbicidal and fungicidal properties are also known.^32^ Such compounds can combine the tebuconazole or propioconazole cation with an herbicidal anion, which results in the formation of ILs with dual properties.^33^ Chemical modification consisting of joining two tebuconazole molecules with an alkyl spacer allows bisammonium cations to be obtained, and their combination with herbicidal anions results in compounds with both herbicidal and fungicidal properties.^34^ Bifunctional HILs are an example of compounds in which biological activity is derived from both the anion and the cation. The transformation of MCPA and 2-chloroethyltrimethylammonium chloride into an IL results in a product that exhibits both an anion-derived herbicidal effect and cation-derived growth regulator activity.^35^ The combination of herbicidal dicamba with tropine characterized by regulatory properties has also been reported.^36^ Double-salt herbicidal ionic liquids (DSHILs) are a particular example of ILs with activity derived from more than one ion. In their case, the ammonium cation is converted into an ionic liquid with two anions in a fixed proportion. DSHILs designed in this way are characterized by herbicidal activity derived from both anions and by physicochemical properties provided by the cation.^37,38^ The most advanced strategy for the synthesis of DSHILs is the combination of two anions with biological activity with a bisammonium cation containing two quaternary nitrogen atoms. Such compounds may include one anion with herbicidal properties and another of natural origin, which leads to unique physicochemical and herbicidal properties.^39^ HILs are characterized by improved properties compared to commercially available products containing the same active substances. Through the use of a rationally designed cation, it is possible to obtain a compound with improved physicochemical properties that translate into herbicidal activity.^40^

Considering the above-mentioned information, it seems reasonable to assume that attention should be focused on the combination of natural and synthetic auxins and the possibility of converting them into DSILs. The aim of this study was to develop sophisticated methods for the synthesis of efficient and environmentally friendly DSILs containing bisammonium cations and both MCPA and l-tryptophanate anions. In addition, the herbicidal and antimicrobial activities of the obtained DSILs were evaluated. To complete the analysis of the results, the influence of anions combination in DSILs on the tested physicochemical and biological properties was also investigated.

## 2 Results and Discussion

### 2.1 Synthesis

**Scheme 1:**
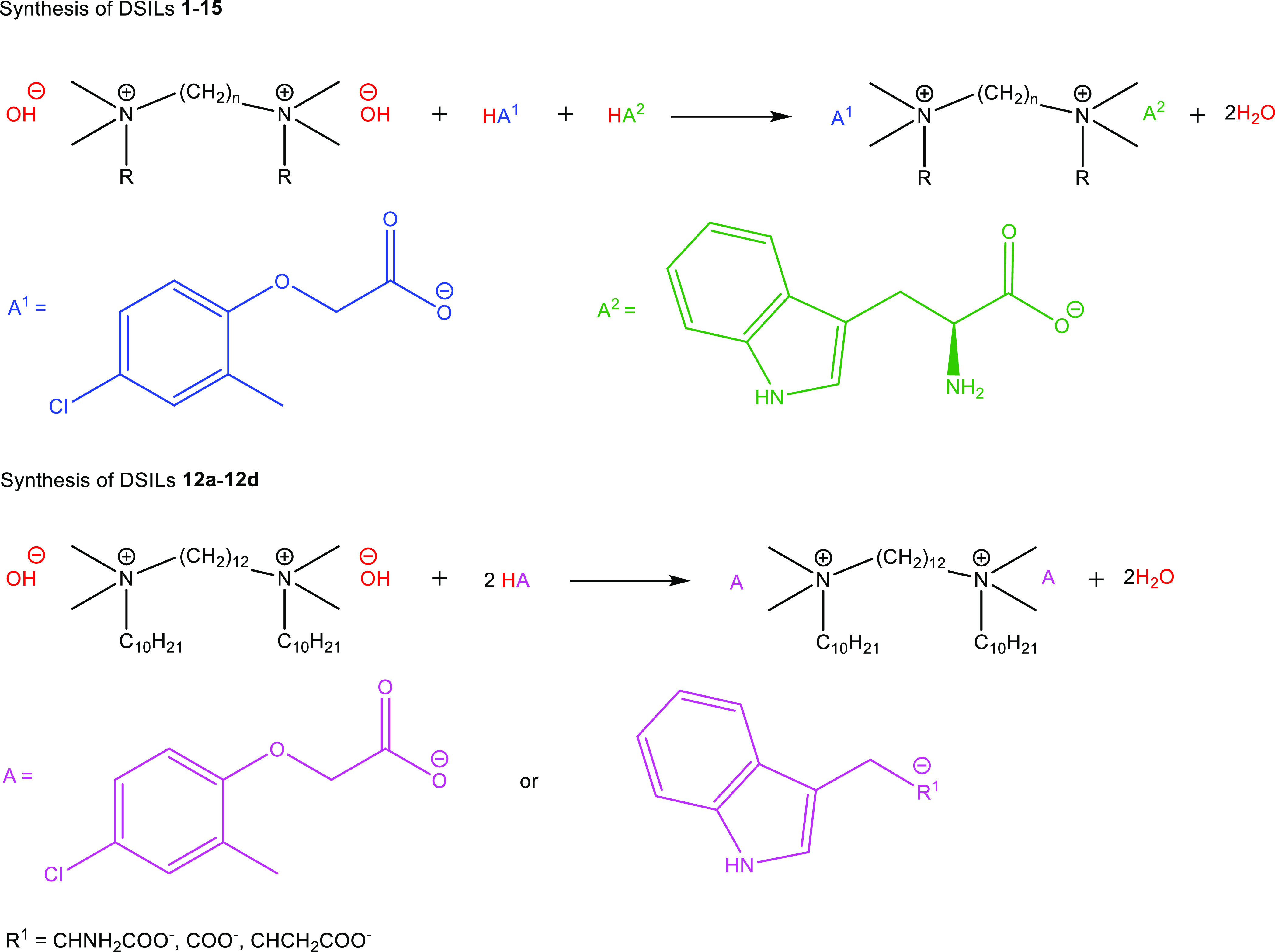
Synthesis of DSILs **1**–**15** and **12a**–**12d**

The DSILs presented in Table 1 were synthesized by a two-step process. In the first step of the synthesis, the bromide anions of the bisammonium dibromide were exchanged for hydroxide anions using a highly alkaline ion-exchange resin in anhydrous methanol, according to the method previously described by our research team.^39^ The second step used an Easy-Max reactor to perform a direct acid-base reaction between the bisammonium hydroxides, MCPA and TRP at a 1:1:1 stoichiometric ratio or between bisammonium hydroxides and auxins (MCPA, TRP, IAA, or IBA) at a 1:2 stoichiometric ratio. The reactions (Scheme 1) occurred immediately at room temperature. The synthesis process was efficient and resulted in high yields ranging from 95 to 99%. The halide level determined by the AgNO_3_ test was below 500 ppm for all prepared DSILs. For all obtained DSILs the water content was determined by the Karl–Fischer method. The water content did not exceed 600 ppm. All obtained DSILs were liquids at 25 °C. The exchange of bromide anions with organic anions lowered the melting points by ∼100 to 270 °C compared to their precursors.^18,39,41−43^ The results of elemental analysis (CHN) are included in the Supporting Information (Table S1) and confirmed the high purity of the obtained DSILs. The compounds were not previously described in the literature.

**Table 1:** Yields of the Synthesized DSILs and the Results of DSC/TG Analysis^a^

DSILs	–(CH_2_)*_n_*–	R	yield [%]	molar ratio of anions MCPA:TRP	*T*_g_ [°C]	*T*_c_ [°C]	*T*_m_ [°C]	*T*_5%_ [°C]	*T*_50%_ [°C]
**1**	–(CH_2_)_6_–	C_8_H_17_	97	1:0.94	18			139	241
**2**	–(CH_2_)_6_–	C_10_H_21_	97	1:0.93	24			147	258
**3**	–(CH_2_)_6_–	C_12_H_25_	96	1:1.01	23			159	272
**4**	–(CH_2_)_6_–	C_14_H_29_	99	1:1.01	11			162	288
**5**	–(CH_2_)_6_–	C_16_H_33_	97	1:1.03	17	33	79	160	289
**6**	–(CH_2_)_8_–	C_8_H_17_	99	1:0.95	8			136	250
**7**	–(CH_2_)_8_–	C_10_H_21_	95	1:0.97	15			132	266
**8**	–(CH_2_)_8_–	C_12_H_25_	98	1:1.05	21			129	279
**9**	–(CH_2_)_8_–	C_14_H_29_	98	1:0.93	11			142	299
**10**	–(CH_2_)_8_–	C_16_H_33_	98	1:0.95	14			147	315
**11**	–(CH_2_)_12_–	C_8_H_17_	96	1:0.95	11			136	276
**12**	–(CH_2_)_12_–	C_10_H_21_	96	1:0.93	3			134	284
**13**	–(CH_2_)_12_–	C_12_H_25_	96	1:0.93	2			135	293
**14**	–(CH_2_)_12_–	C_14_H_29_	95	1:0.93	15			142	325
**15**	–(CH_2_)_12_–	C_16_H_33_	95	1:0.96	10			131	328
**12a**	–(CH_2_)_12_–	C_10_H_21_	97	2:0	–27			213	271
**12b**	–(CH_2_)_12_–	C_10_H_21_	98	0:2	16			152	315
**12c**	–(CH_2_)_12_–	C_10_H_21_	96	0:2 (IAA)	–14			213	282
**12d**	–(CH_2_)_12_–	C_10_H_21_	97	0:2 (IBA)	–21			238	296

a *T*_m_, melting point; *T*_c_, temperature of crystallization; *T*_g_, glass-transition temperature; *T*_5%_, decomposition temperature of 5% of the sample; *T*_50%_, decomposition temperature of 50% of the sample.

**Figure 1:**
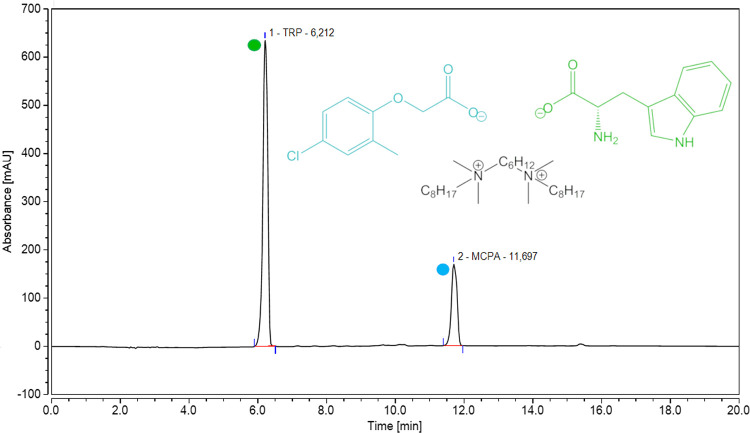
HPLC chromatogram of DSIL **1**.

The structures of the synthesized DSILs were confirmed by nuclear magnetic resonance (NMR) and Fourier transform infrared (FT-IR) spectroscopy analyses, and the results are included in the Supporting Information (Table S1 and Figures S1–S57). In the ^1^H NMR spectra of the DSILs (**1**–**15**, **12a**–**12d**), chemical shifts originating from the cation and both anions were observed. Characteristic signals associated with the TRP anion occurred at ∼3.65 (from the CH–NH_2_ proton) and 7.18 from the CH group in the pyrrole ring. Characteristic resonance signals of the MCPA anion occurred at σ [ppm] = 2.25 (from the CH_3_ group of the aromatic ring) and 4.40 (from the CH_2_-COO^–^ group). For the IAA anions, characteristic resonance signals distinguishing them from the TRP anions occurred at ∼3.59 ppm (from the CH_2_ group), and for the IBA anions—at 2.35, 2.02, and 2.80 ppm (respectively, from the CH_2_–CH_2_–CH_2_ groups). In turn, the resonance signals at ∼1.70 to 0.90 ppm confirm the presence of carbon chains in the bisammonium cation. In the ^13^C NMR spectrum, there were two signals characteristic of carboxyl groups, located at ∼180 ppm for the TRP anion and at ∼176 ppm for the MCPA anion. A resonance signal at ∼16 ppm confirms the presence of a methyl group attached to the aromatic ring in the MCPA anion. For the IAA anions, the characteristic chemical shifts distinguishing them from the TRP anions occurred at σ [ppm] = 30.65 ppm (from the CH_2_ group), and those distinguishing them from the IBA anions occurred at ∼27.35, 27.15, and 30.55 ppm (respectively from the CH_2_–CH_2_–CH_2_ groups). The presence of the bisammonium cation was confirmed by resonance signals in the range of 31–14 ppm. In the FT-IR spectra, the characteristic signals from the TRP anion appeared at the following approximate *V*_max_ values [cm^–1^]: 650 (benzene ring), 745 (CH_2_, rocking vibration), 880 (substituted ring 1,4 distribution), 997 (C–N, stretching vibration), 1097 (aryl group), 1140 (C–C, stretching vibration), 1230 (CH_2_ wagging vibration), 1400 (−C=O, stretching vibration), 1460 (−COO^–^, stretching vibration), and 1560 (C=O, stretching vibration). The presence of the MCPA anion was confirmed by signals with the following *V*_max_ [cm^–1^] values: 721 (Cl–C, stretching vibration), 800 (substituted ring 1,2 distribution), 900 (substituted ring 1,1 distribution), 1240 (=C–O–C, stretching vibration), and 1500 (−COO^–^, stretching vibration). After analyzing the FT-IR spectra of DSILs **12a**–**12d**, all signals characteristic of TRP, MCPA, IAA, and IBA anions were observed. Comparing the spectra of compounds **12b** and **12c** with that of **12d**, the differences in the spectra were small—the signal from the C–N group at 997 cm^–1^ disappeared, and an additional signal appeared at a *V*_max_ of 721 cm^–1^ (−CH_2_–, rocking vibration). Characteristic signals of bisammonium cations were recorded in the range of 2850–3000 cm^–1^, originating from the CH_alif_ groups. ILs containing TRP and MCPA anions (**1**–**15**) were subjected to quantitative high-performance liquid chromatography (HPLC) analysis. The application of ion pair chromatography allowed the separation and quantification of both anions. The retention times were equal to 6.2 and 11.7 min for TRP and MCPA anions, respectively. An exemplary chromatogram is presented in Figure 1. The operating conditions are included in the Supporting Information (Figure S58).

The measured retention times matched the retention times of the standards. Based on the calibration curves, the molar ratio of the anions was calculated (Table 1). HPLC chromatograms are included in the Supporting Information (Figures S58–S74). Both anions were confirmed to be present in equimolar amounts. In the case of monoanionic ILs, the concentrations of MCPA and TRP were confirmed for **12a** and **12b**, respectively. The observed variations did not exceed the measurement error. NMR spectroscopy, FT-IR and HPLC analyses fully confirmed the structures of the synthesized DSILs.

### 2.2 Thermal Analysis

In the literature regarding a series of bis-cationic compounds, the influence of the cation core (imidazolium, ammonium, or phosphonium) and spacer length on the thermal properties has been widely described.^44−46^ Described systems containing two identical cations were mainly influenced by the incorporation of two different cation cores into one compound, which resulted in the formation of liquid crystals.^45−47^ Most bis-cationic compounds described in the literature include a single popular type of anion, e.g., major halides such as bromide, chloride, or NTf_2_ and BF_4_ in the case of ILs. On the other hand, the effect of the incorporation of two different anions and one cation was described in a monocationic system containing fractional anions at a molar ratio of glyphosate/MCPA equal to 8.48:0.152, and the thermal behavior of the product underwent only slight changes.^38^ Similarly, in studies regarding the combination of the ammonium cation and the MCPA–Dikamba anionic system, no significant changes in thermal properties were observed.^37^ However, the results of our research examining equimolar amounts of TRP-MCPA anions showed more drastic changes in the thermal properties of the resulting ILs. For all prepared DSILs (Table 1), only a glass transition occurred, which suggests that the incorporation of the examined anion system reduced the molecular order to a minimum. Most likely, the most ordered system occurs in the same cation with two bromide anions, which was previously described in the literature.^45^ For example, in the case of bis(*N*-dodecyl-*N*,*N*-dimethylammonium) dibromides described in the literature with spacers ranging from 2 to 10 carbon atoms, thermal phase transitions, such crystallization and melting points, were observed in all cases. These dibromides also showed correlations between the length of the spacer and the number and type of mesophases (ordered smectic phases and/or sematic liquid crystals). In our study, no similar observation could be established.

For DSILs **1**–**15**, the tendency of a glass transition with the elongation of alkyl substituents was not uniform. In the case of DSILs with a hexyl spacer (**1**–**5**), the glass-transition temperature (*T*_g_) was generally unchanged, ranging from 17 to 24 °C. Only for DSIL **4** did the glass-transition temperature decrease to 11 °C. A similar tendency was observed for DSILs **6**–**10**, with slightly lower glass-transition temperatures equal to 8, 15, and 14 °C for DSILs **6**, **7**, and **10**, respectively. In the case of the dodecyl alkyl substituent (**8**), an increase in *T*_g_ reaching 21 °C was observed. For DSILs **11**–**15** with long spacers, the glass-transition temperatures were lower for decyl (**13**) and dodecyl (**14**) groups, reaching 3 and 2 °C, respectively. In the case of extreme substituent lengths in DSILs **11** and **15**, the glass-transition temperature was higher and reached ∼10 °C. Only in the case of compound **5**, additional crystallization at 33 °C and a melting point at 79 °C were observed. The occurrence of a glass transition indicates an amorphic character of the obtained ILs. Detailed analysis (HPLC) showed that the molar ratios of the anions (MCPA/TRP) ranged from 1:0.93 to 1:1.03 (Table 1). The detailed influence of molar ratios of the anions in the range of 2:0 to 0:2 was studied.

**Figure 2:**
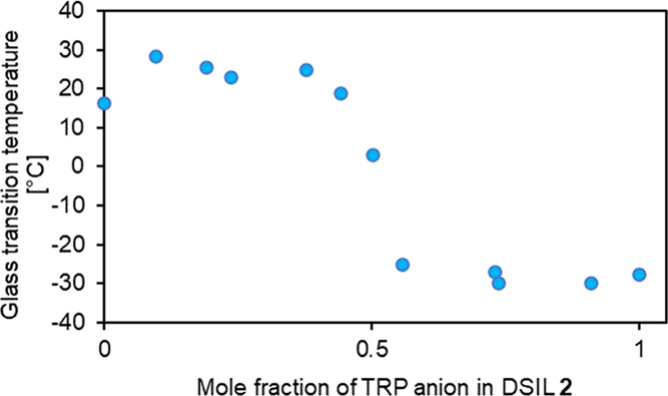
Influence of the molar fraction of the l-tryptophan anion in DSIL **2**.

The influence of l-tryptophan anion molar ration in DSIL **2** is presented in Figure 2. The curve is “s-shaped”. This characteristic is commonly achieved in polymeric systems with various reactive additives. The occurrence of hydrogen bond donors (e.g., NH or NH_2_), acceptors (e.g., carbonyl or ether group), and aromatic rings in both MCPA and TRP anions in the obtained ILs as well as the character of the curve (Figure 2) indicated complex intramolecular interactions. The TGA results indicated that the studied DSILs were thermally stable up to 131 °C. The compound containing only the MCPA anion (**12a**) decomposed at 213 °C, and the DSIL containing the same cation and two anions of TRP (**12b**) decomposed at 152 °C. For DSILs containing a mixed MCPA/TRP anion system with six carbon atoms in the spacer (**1–5**), decomposition occurred from 139 to 160 °C. Elongation of the spacer to eight carbon atoms (**6**–**10**) resulted in a minimal reduction in thermal stability from 129 (**8**) to 147 °C (**10**). Further spacer elongation (**11**–**15**) did not produce significant differences in terms of thermal stability, with decomposition occurring at 131 °C for **15** and 142 °C for **14**. The exchange of one MCPA anion for TRP caused a decrease in the thermal stability of the IL. The elongation of the alkyl substituent lowered the dynamics of thermal decomposition for DSILs (**11**–**15**), and the value of *T*_50%_ increased from 276 to 328 °C with increasing alkyl chain lengths.

### 2.3 Chemical Stability

The chemical stability of aqueous solutions of the obtained DSIL **12** was tested at 80 °C. In the framework of this study, ^1^H and ^13^C NMR spectra were obtained before and after heating. In the ^1^H NMR spectrum of DSIL **12** after heating, a decrease in signal integration and intensity by 20–80% and shifts in resonance signals in the ^1^H spectra by ∼1.5 ppm were observed. Analysis of the ^13^C NMR spectra of DSIL **12** after heating indicated the disappearance of the signals from carbon atoms in the indolyl ring (σ [ppm] = 138.68, 129.43, 125.30, 120.80, 120.10, and 112.39) and the carbon atom in the carboxyl group (σ = 180.94 ppm). The observed differences clearly indicate a change in the chemical structure of DSIL **12** after heating. The conversion of l-tryptophan to an ion reduced its chemical stability, which was also observed in our previous research regarding amino acid ILs with tetraalkylammonium cations.^18,19^ A comparison of the NMR spectra of DSIL **12** and its degradation products is shown in Figures S75 and S76.

### 2.4 Solubility Studies

The solubility test was carried out using popular organic solvents in ascending order of polarity index values according to Snyder’s scale. The results of the test are presented in Table 2.

**Table 2:**
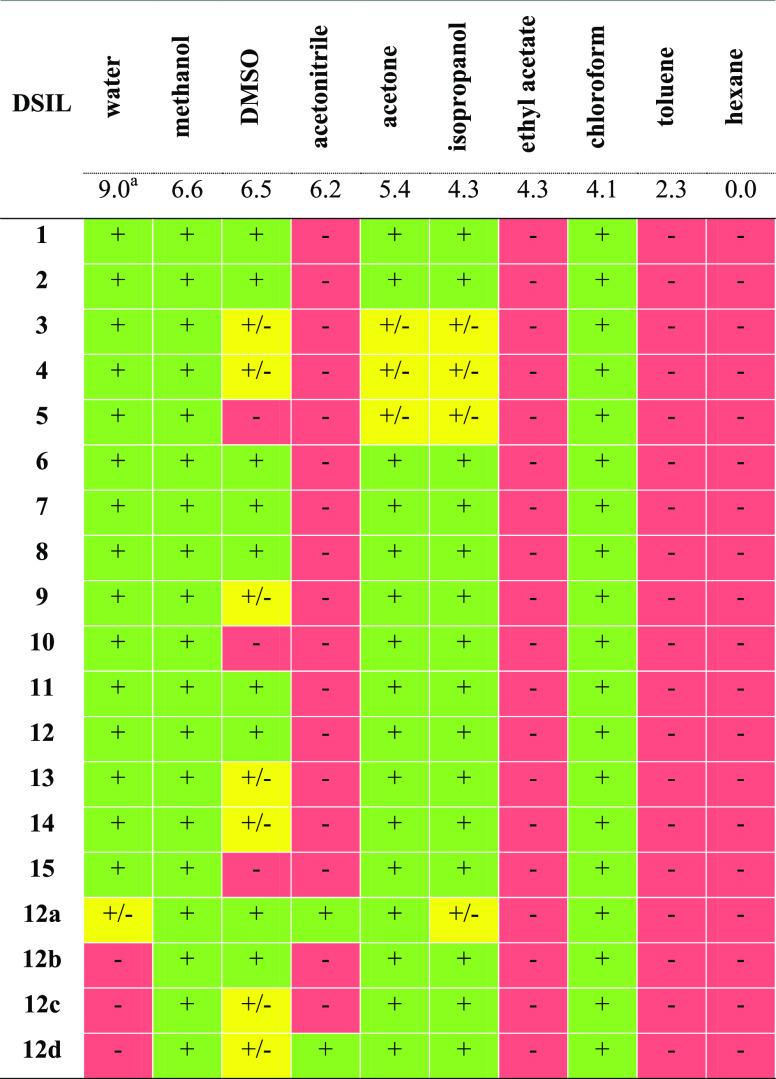
Solubility of the Synthesized DSILs at 25 °C^a^

a Snyder polarity index, “+” high solubility, “+/–” moderate solubility, “–” low solubility.

The tested DSILs **1**–**15** were insoluble in low-polar and apolar solvents such as ethyl acetate, toluene, or hexane. The exception was chloroform, in which all salts were soluble despite its low polarity and aprotic nature, which may have been related to its donor ability to form hydrogen bonds with the tested salts.^39,48^ The obtained DSILs **1**–**15** were soluble in polar solvents. The exception was dimethyl sulfoxide (DMSO), in which the solubility decreased with the elongation of the R substituent in the bisammonium cation. Comparison of the obtained results with our previous studies indicated that the introduction of the l-tryptophan anion increased the solubility of all DSILs in the tested solvents.^39^ The solubility of l-tryptophan and MCPA in water is limited, at 11 and 725 mg·L^–1^, respectively.^49,50^ The conversion and combination of l-tryptophan and MCPA into ionic forms resulted in an over 100-fold improvement of their solubility in water. In the case of DSILs containing the same two anions (**12a**–**12d**), a decrease in water solubility was observed compared to the analogue of DSIL **12**, which was caused by the higher molar fraction of the anion that was almost insoluble in water.

### 2.5 Surface Activity

**Figure 3:**
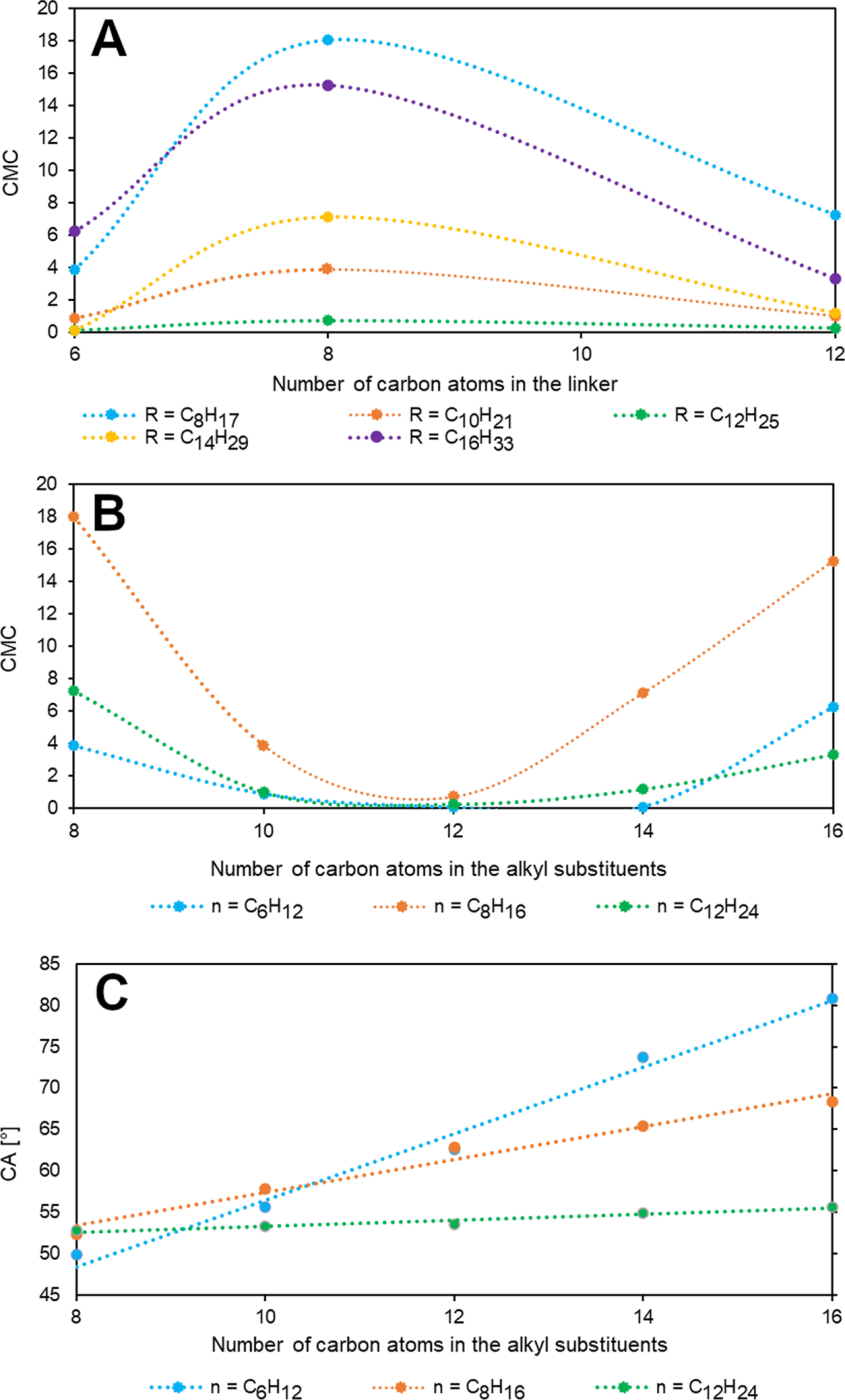
Impact of number of carbon atoms in the spacer on CMC (A) and the number of carbon atoms in the alkyl substituent on CMC (B) and CA (C) in DSILs **1**–**15** at 25 °C.

Surface activity was determined for aqueous solutions of DSILs **1–15** in the concentration range of 5 × 10^–2^ to 2 × 10^–6^ mol·L^–1^ at 25 °C. The following surface activity parameters were evaluated: the critical micelle concentration (CMC), surface tension at CMC (γ_CMC_), efficiency of surface adsorption at an air–water interface (pC_20_), and contact angle (CA). The results are included in the Supporting Information (Tables S2–S4 and Figure S77). The determined surface tension at the CMC point ranged between 33 and 43 mN·m^–1^. The lowest CMCs occurred for DSILs **3**, **8**, and **13** with dodecyl substituents. For all DSILs, the CMC parameter increased with elongation from the hexyl to octyl spacers and then decreased (Figure 3A). After analyzing the influence of the alkyl substituents, it was noted that the CMC parameter decreased with elongation from the octyl to dodecyl substituents and then increased from the dodecyl to hexadecyl substituents (Figure 3B). The CMC value ranged from 18 to 0.9 mmol·L^–1^. The relationship between the CA value and the length of the alkyl group is presented in Figure 3C. The value of CA increased linearly with the elongation of the alkyl substituent in the bisammonium cation, which resulted from higher hydrophobic interactions between the longer chains.^51,52^ The elongation of the alkyl substituent resulted in an increase in the contact angle from 50 to 81° for **1**–**5**, from 52 to 68° for **6**–**10**, and from 52 to 56° for **11**–**15**. DSILs with a 1,12-dodecylene spacer (**11**–**15**) exhibited the lowest contact angles. In the case of the spacer, it was observed that the wettability of the DSILs improved with elongation. The surface activity indicated an increase in the wettability of the paraffin surface that resembled a plant surface, which was associated with enhanced absorption of the active substance by plants.

### 2.6 Herbicidal Activity

**Figure 4:**
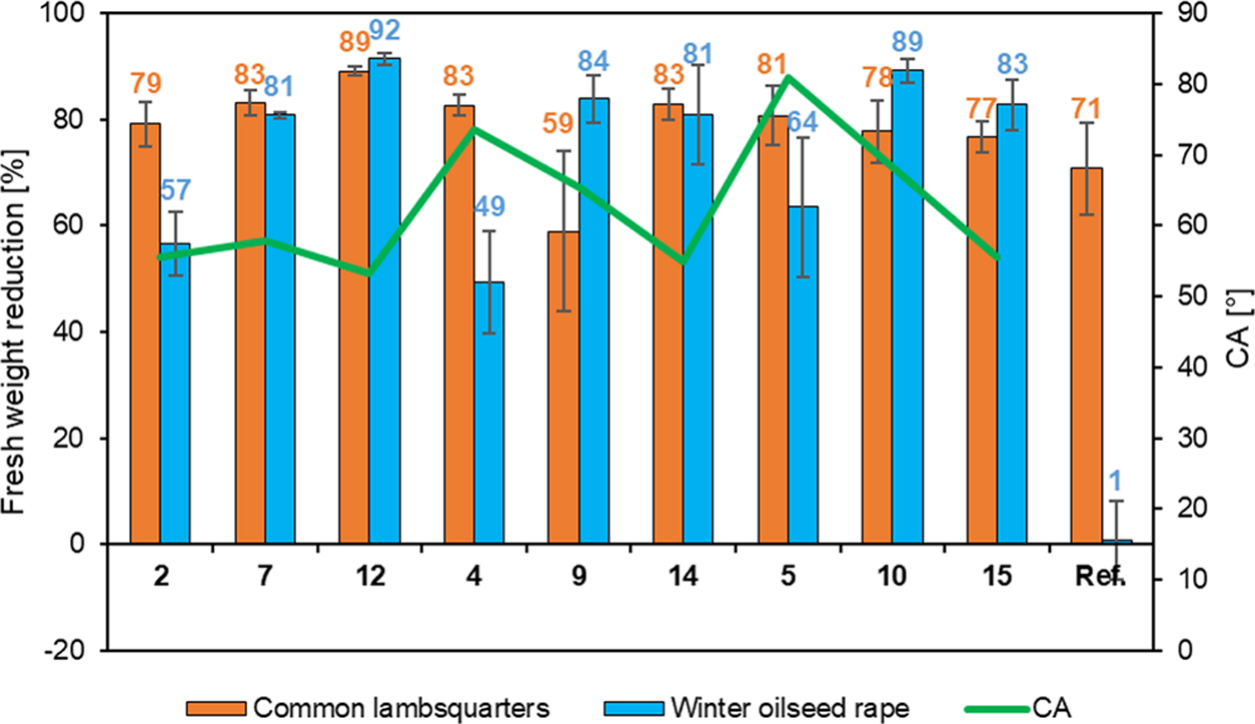
Impact of contact angles of tested aqueous DSHILs on herbicidal activity against common lambsquarters and winter oilseed rape.

**Figure 5:**
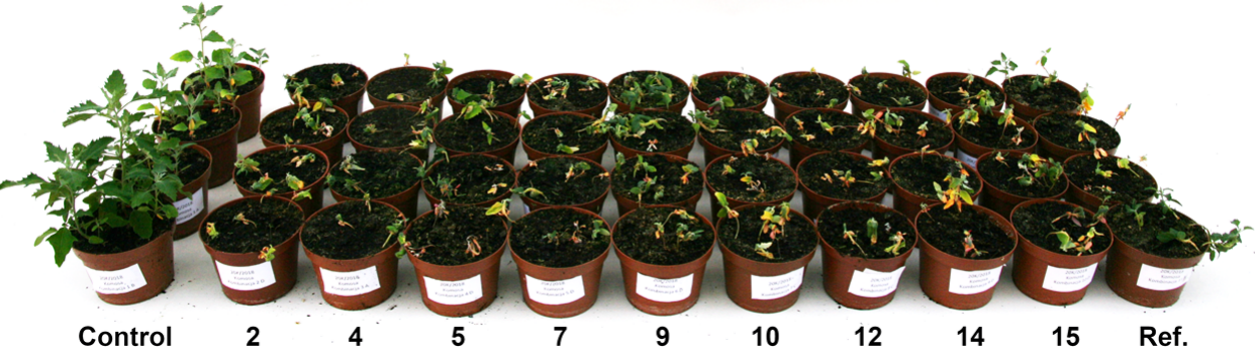
Effect of tested DSHILs on the condition of common lambsquarters.

The herbicidal activity of the obtained DSILs was determined on the basis of biological tests performed in a greenhouse using common lambsquarters (*Chenopodium album* L.) and winter oilseed rape (*Brassica napus* L.) as test plants. The test results for DSILs **2**, **4**, **5**, **7**, **9**, **10**, **12**, **14**, and **15** are presented in Figure 4. The greenhouse test confirmed that most of the synthesized DSILs showed higher herbicidal effectiveness than the commercial herbicide containing MCPA in the form of potassium and sodium salts. The observed symptoms of damage to plants sprayed with the tested DSILs were typical for herbicides from the phenoxyacid derivative group, i.e., deformation of leaves and shoots, chlorosis, and necrosis in the final stage, as shown in Figure 5.^53^ The largest difference was noted for winter oilseed rape, against which DSILs were more effective than commercial herbicides by over 49%. The use of two different anions in DSIL synthesis was the factor that contributed to the high biological activity since both the MCPA anion and the TRP anion inhibit the growth of plant cells at appropriate doses.^22,54^ The positive effect of the introduction of an anion of natural origin on herbicidal activity can be observed by comparing DSILs **1**–**15** with previously described DSHILs and HILs containing the MCPA anion.^39^ On the basis of the analysis, it was noted that the combination of a natural anion with a plant growth-promoting effect (TRP) with a synthetic auxin anion (MCPA) and an amphiphilic cation allowed an increase in herbicidal activity. In all cases, the DSIL dose resulted in an up to 6 times greater reduction in fresh oilseed rape weight compared to the reference substance. In the case of common lambsquarters, the effectiveness of all DSILs was 6–19% higher than that of the reference herbicide. The exception was DSIL **9**, which was 59% effective. The biological activity of chemical compounds is largely related to the surface activity and contact angle.^52^ The effect of the contact angle on the reduction in fresh weight of the tested plants is presented in Figure 4. It was observed that the wetting angle decreased and the herbicidal activity increased with the elongation of the spacer in the cation, which was also observed in our earlier studies.^39^ The obtained DSILs exhibited good wettability of the hydrophobic surface that imitated a leaf surface and high herbicidal activity against common weeds. An additional advantage of the synthesized DSILs is their application in lower doses than commercial agents while maintaining high efficiency. The modification of l-tryptophan and MCPA and their combination with an amphiphilic cation allowed us to obtain new effective double-salt herbicidal ionic liquids (DSHILs).

### 2.7 Antimicrobial Activity

**Figure 6:**
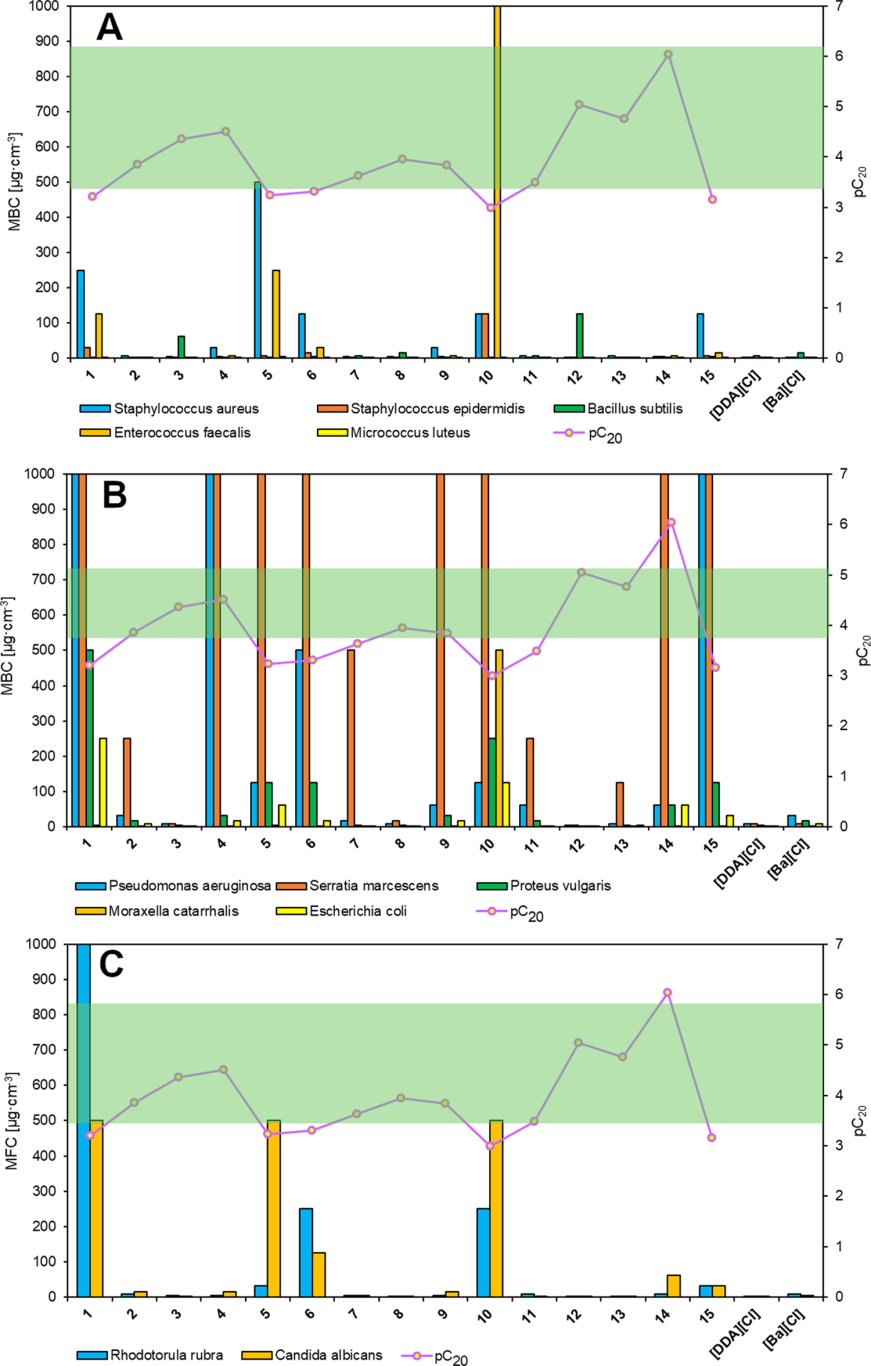
Influence of pC_20_ on MBC against Gram-positive bacteria (A), Gram-negative bacteria (B), and MFC against yeasts (C).

Microbial contamination is a common phenomenon that is harmful to animal and human health; hence, the antimicrobial properties of the studied DSILs were tested. The biological activity of DSILs **1**–**15** was evaluated using Gram-positive and Gram-negative bacteria as well as yeasts. The obtained results are presented in Figure 6A–C, along with the analysis of the influence of the pC_20_ parameter on the antimicrobial activity. The test was repeated after 4 weeks to determine the effect of chemical stability on the antimicrobial activity. The calculated mean values of the minimum inhibitory concentration (MIC), minimum bactericidal concentration (MBC), and minimum fungicidal concentration (MFC) are included in the Supporting Information (Tables S5–S8). All studied DSILs showed biological activity against all test microorganisms. The strongest inhibitory effects against the growth of the tested microorganisms were observed for DSILs **3**, **8**, **12**, and **12a**–**12d**, the biological activity of which was higher than or comparable to that of the didecyldimethylammonium chloride [DDA][Cl] and benzalkonium chloride [BA][Cl] used for comparative purposes. The DSILs exhibited strong antimicrobial effects toward Gram-positive and Gram-negative bacteria as well as yeasts. The antimicrobial activity of the tested DSILs remained at a comparable level after a month of storage, and a slight decrease in biological activity was observed only in a few cases. The literature states that the biological activity of DSILs increases with the elongation of the alkyl substituents, which was also observed in our study for compounds **1**–**15**.^36,55^ After analyzing the influence of the chemical structure of the bisammonium cation, it was noted that the antimicrobial activity increased with the elongation of the R substituent—the values of the MIC, MBC, and MFC parameters decreased. For the DSILs (**5**, **10**, and **15**) with the longest R substituents in the bisammonium cation (R = 16), an increase in antimicrobial activity was not observed. This phenomenon was described in the related literature as a “cut-off”, which applies to ILs and cationic surfactants.^56,57^ Comparing the results obtained for DSILs **12** and **12a**–**12d**, it was noted that the use of two anions with the same chemical structure increased the activity only against *Pseudomonas aeruginosa* and *Serratia marcescens*. For the remaining microorganisms, the antimicrobial and antifungal activities did not change, which may have been related to the progressive degradation of anions. The role of the bisammonium cation was thus dominant.^58^ The antimicrobial activity of the applied DSILs was greatly influenced by the surface activity, e.g., the parameter pC_20_.^56^ For the obtained DSILs, the highest antimicrobial efficacy was recorded in the optimal pC_20_ range. In the case of DSILs that exhibited lower or higher pC_20_ values than the optimum, lower antibacterial and antifungal efficacy was observed. In addition, a parabolic relationship was observed between the MIC or MFC and pC_20_, which has also been reported in the literature.^56,57^ After comparing the impact of the tested DSILs on the growth of individual groups of microorganisms, it can be concluded that Gram-positive bacteria and yeasts showed greater sensitivity to applied compounds than Gram-negative bacteria. *Moraxella catarrhalis* showed the highest sensitivity among Gram-negative bacteria, while *Micrococcus luteus* was the most sensitive among Gram-positive bacteria.

## 3 Conclusions

This study presents the synthesis and characterization of a homologous series of novel DSILs, which comprise a single cation and two different anions. The concept of combining bisammonium cations with different alkyl substituents with l-tryptophanate and MCPA as anions allowed us to obtain a group of compounds with a set of properties that perfectly fit the requirements of modern agrochemicals. A series of analyses, including NMR, FT-IR, and HPLC, confirmed that the employed synthesis method is feasible for obtaining the target structures with high yields (95–99%) and equimolar ratios of anions. Subsequent studies (i.e., elemental analysis, assessments of halide and water contents, and DSC/TG) allowed us to determine that the DSILs were characterized by high purity and thermal stability. It was observed that the length of the spacer and alkyl substituent in the cation notably influenced the studied physicochemical properties (thermal behavior, solubility in different solvents, and surface activity), which allowed us to design the structures of the obtained compounds in accordance with needs. The high water solubility resulting from the conversion of l-tryptophan and MCPA to anionic forms is of particular importance from the practical perspective of preparing spray solutions. The subsequent evaluation of biological activity indicated that all of the studied DSILs exhibited high herbicidal activity, which, in most cases, exceeded that of the reference commercial herbicide agent (even by ∼50%). Their high efficiency in eliminating weeds corresponds well with their high wettability of hydrophobic surfaces, i.e., high affinity to interact with plant leaves. Each of the studied compounds also exhibited excellent antimicrobial activity, which was comparable to or higher than that of didecyldimethylammonium chloride and benzalkonium chloride. The low MIC and MBC/MFC values may be attributed to the high surface activity of the cationic component and can be considered another merit, as the compounds may protect crops from pathogenic microorganisms. It should be noted that l-tryptophan as an anion is characterized by lower stability; hence, the compounds should be stored under appropriate conditions. However, its degradation occurs slowly and does not affect the functional properties in a significant manner. The presented results confirm the high application potential of the described DSILs and provide a new and promising path for obtaining new and efficient plant-protection agents.

## 4 Materials and Methods

### 4.1 Materials

The following chemicals were purchased from Sigma-Aldrich (Poznan, Poland): 1,6-dibromohexane (purity 96%), 1,8-dibromooctane (purity 98%), 1,12-dibromododecane (purity 98%), dimethyloctylamine (purity 97%), decyldimethylamine (purity 90%), dimethyldodecylamine (purity 97%), dimethyltetradecylamine (purity 95%), hexadecyldimethylamine (purity 95%), l-tryptophan (purity 98%), and Dowex-Monosphere 550A anion exchange resin. (4-Chloro-2-methylphenoxy)acetic acid [MCPA] (purity 97%) was obtained from CIECH Sarzyna (Nowa Sarzyna, Poland). All solvents (methanol, acetonitrile, acetone, hexane, toluene, chloroform, isopropanol, DMSO, and ethyl acetate) were from Avantor (Gliwice, Poland). Deionized water with conductivity <0.1 μS·cm^–1^, from demineralizer HLP Smart 1000 (Hydrolab, Poland), was used for solubility and surface activity measurement. All reagents and solvents were used without further purification. As a reference, Chwastox Extra 300 SL (300 g MCPA per 1 L, CIECH Sarzyna, Poland) was used as a commercial herbicide. Microbiological media used for the studies were purchased in BioMaxima (Poland).

### 4.2 Synthesis

The appropriate bisammonium dibromide (0.05 mol) obtained in our earlier studies^39^ was dissolved with 50 mL of anhydrous methanol in a 100 mL reaction glass equipped with a mechanical stirrer. The anionic resin Dowex-Monosphere 550A (40 mL) in the form of a methanolic suspension was added, and the mixture was stirred for 8 h at 25 °C. After the anion exchange reaction, the resin was filtered and rinsed three times using small amounts of methanol. Then, a stoichiometric amount of l-tryptophan and MCPA was added to the bisammonium hydroxide solution. All of the neutralization reactions were conducted at 25 °C in a Mettler Toledo semiautomated system reactor equipped with a glass electrode. The solvent was evaporated under vacuum in a rotary evaporator, and the product was dried under reduced pressure (5 mbar) at 40 °C for 48 h. The synthesized DSILs were stored in UV-blocking vacuum desiccators over P_4_O_10_ at 20 °C.

### 4.3 Analysis

The ^1^H and ^13^C NMR studies were performed using a Varian XL300 spectrophotometer. Deuterated methanol was used as the solvent, and tetramethylsilane was the reference. The elemental analyses (CHN) were performed using an Elementar Analyser Vario EL III at the Adam Mickiewicz University, Poznan (Poland).

FT-IR spectra were recorded using a semiautomated EasyMax 102 system connected with a ReactIR 15 spectrometer with a probe and MCT detector and a 9.5 mm AgX probe with a diamond tip (Mettler Toledo system). The data were collected from 640 to 3000 cm^–1^ with high resolution.

HPLC analyses were conducted using a Dionex UltiMate 3000 unit equipped with a UV-DAD detector operating at 280 nm wavelength. Thermo Scientific Hypersil Gold C18 150 mm/4.6mm with a stationary particle size of 5 μm was used as the column. The mobile phase was a gradient mixture of 0.1 M ammonium formate, 10% v/v formic acid, and acetonitrile in ratios of 99:1:0 at 0 min, 25:1:74 at 15 min, and 99:1:0 at 20 min under a 1 mL**·**min^–1^ flow rate. Time of analysis was 20 min, and injection volume was equal to 20 μL. Concentrations were determined on the basis of calibration curve in the range of 10–500 ppm. The water content was determined using an Aquastar volumetric Karl–Fischer titration with Composite 5 solution as the titrant and anhydrous methanol as a solvent.

### 4.4 Thermal Analysis

The TGA/DSC1 Mettler Toledo apparatus was used. The thermal analysis apparatus was calibrated by measuring the following standards: In (purity 99.999%, *T*_m_ = 156.49 °C, Δ*H* = 29.38 J·g^–1^), Pb (purity 99.99%, *T*_m_ = 327 °C, Δ*H* =22.25 J·g^–1^), Zn (purity 99.998%, *T*_m_ = 418.78 °C, Δ*H* = 106.53 J·g^–1^), Al (purity 99.99%, *T*_m_ = 660.03 °C, Δ*H* = 340.15 J·g^–1^). Thermogravimetric measurements were performed at 10 °C·min^–1^ from room temperature to 500 °C under dynamic nitrogen atmosphere (50 mL·min^–1^), using about 4–5 mg of sample in aluminum pans. Thermal transition temperatures were determined by DSC, with a Mettler Toledo Star DSC1, under dynamic nitrogen atmosphere (50 mL·min^–1^), using about 4–5 mg of sample in aluminum pans. Measurements were performed in the range −100 up to 105 °C.

### 4.5 Chemical Stability

To evaluate the chemical stability of a selected DSIL, 0.1 g was dissolved in 50 mL of water and heated at 80 °C for 2 days. The solvent was then evaporated using a rotary evaporator. The obtained compound was dried using a vacuum dryer at 60 °C for 24 h. Changes in the structure of the compound were determined on the basis of NMR spectra analysis.

### 4.6 Solubility

Water and nine popular organic solvents characterized by varying polarity were selected for the solubility test and arranged in order of decreasing Snyder’s polarity index: water, 9.0; methanol, 6.6; DMSO, 6.5; acetonitrile, 6.2; acetone, 5.1; isopropanol, 4.3; ethyl acetate, 4.3; chloroform, 4.1; toluene, 2.3; and hexane, 0.0. The solubility of the obtained salts in organic solvents was determined according to the methodology previously described in Vogel’s Textbook of Practical Organic Chemistry.^59^ The sample of DSILs (0.1000 ± 0.0001 g) was introduced into a specific volume of solvent. The analyses were performed at 25 °C. Depending on the volume of solvent used, three outcomes have been recorded: “high solubility” applies to salts that dissolved in 1 cm^3^ of the solvent, “limited solubility” applies to compounds that dissolved in 3 cm^3^ of the solvent, and “low solubility” applies to DSILs that did not dissolve in 3 cm^3^ of the solvent.

### 4.7 Surface Activity

Surface tension and contact angle values of DSILs were determined by the use of a DSA 100 analyzer (KRÜSS GmbH, Germany, accuracy ± 0.01 mN·m^–1^) at 25 °C. The surface tension was carried out using the shape drop method. Basically, the principle of this method is to form an axisymmetric drop at the tip of a needle of a syringe. The image of the drop (3 mL) is taken from a CCD camera and digitized. The surface tension (γ in mN·m^–1^) is calculated by analyzing the profile of the drop according to the Laplace equation. Temperature was controlled using a Fisherbrand FBH604 thermostatic bath (Fisher, Germany, accuracy ± 0.1C). The values of the critical micelle concentration (CMC) and the surface tension (γ_CMC_) at CMC were determined from the intersection of the two straight lines drawn in low- and high-concentration regions in surface tension curves (γ vs log *C* curves) using a linear regression analysis method. Surface active parameter such as the adsorption pC_20_ (C_20_ is the molality of compound which leads to a reduction of the surface tension of the solvent by 20 mN·m^–1^) is defined using the following equation
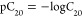
1The contact angle (CA) was determined by the sessile drop method, where drops of liquid above CMC were deposited on a solid paraffin surface (capable of imitating the hydrophobic surface of the leaf). After the actual drop shape and contact line are defined, the drop shape is adapted to fit a mathematical model used to calculate the contact angle. The most precise method to calculate this value is the Young–Laplace fitting (sessile drop fitting), in which the entire drop contour is evaluated. After successful fitting of the Young–Laplace equation, the CA is determined as the slope of the contour line at the three-phase contact point (solid–liquid and liquid–air).

### 4.8 Herbicidal Activity

The biological studies were performed in a greenhouse with controlled environmental conditions: a temperature of 20 (±2) °C, humidity of 60%, and a photoperiod of 16/8 h day/night using common lambsquarters (*C. album* L.) and winter oilseed rape (*B. napus* L.) as the test plants. The plants were grown in 0.5 L plastic pots containing commercial peat-based potting material (pH 6). After emergence, the plants were thinned to five in each pot. Treatments were applied at the 4–6 leaf growth stage. The commercial product Chwastox Extra 300 SL (300 g of sodium and potassium salts of MCPA in 1 L) was used as a reference. DSILs were applied at a dose corresponding to 300 g of active ingredient (MCPA) per 1 ha. Commercial herbicide was used at the same doses of active ingredient. All tested DSILs and reference compounds were dissolved in water. The applications were conducted using a moving sprayer (APORO, Poznan, Poland) with a flat-fan nozzle (TeeJet Technologies, Wheaton, IL) delivering 200 L·ha^–1^ of spray solution at an operating pressure of 0.2 MPa. The nozzle was moved above the plants at a 40 cm distance and at a constant speed of 3.1 m·s^–1^. After treatment, the plants were again placed in a greenhouse under the environmental conditions mentioned above. The fresh weight of the plants was measured 2 weeks after treatment using a technical balance with an accuracy of 0.01 g (Sartorius BP 2000 S, Sartorius Göttingen, Germany). The study was carried out in four replications in a randomized setup. The error margin range represents standard errors of the mean (SEM). The SEM values were calculated according to the following equation
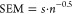
2where SEM is the standard error of the mean, *s* is the sample standard deviation, and *n* is the number of replications.

### 4.9 Antimicrobial Activity

Antimicrobial tests were conducted using Gram-positive bacteria *Staphylococcus aureus* ATCC 33862, *Staphylococcus epidermidis* ATCC 12228, *Enterococcus faecalis* ATCC 19433, *Bacillus subtilis* ATCC 11774, and *M. luteus* ATCC 4698; Gram-negative bacteria *Escherichia coli* ATCC 8739, *P. aeruginosa* ATCC 9027, *S. marcescens* ATCC 8100, *Proteus vulgaris* ATCC 49132, and *M. catarrhalis* ATCC 25238; and yeasts *Candida albicans* ATCC 10231 and *Rhodotorula rubra*. Before the experiments, the microorganisms were propagated on appropriate agar media for 24 h. The experiments were performed on the day of complete drying of the obtained DSILs (29/09/2020) and after a 30-day period of storage of the samples under 5 °C refrigeration conditions (28/10/2020). The samples were stored refrigerated and protected from light. In each case, the ionic liquids were dissolved in water, resulting in a starting concentration of 2000 μg·mL^–1^. The minimum inhibitory concentration (MIC) and the minimum bactericidal/fungicidal concentration (MBC/MFC) values were determined by the twofold dilution method in 96-microtiter plates. Twenty-four-hour cultures of microorganisms on slants were used to prepare a suspension in saline to achieve a 0.5 MacFarland density. Next, the solutions were diluted in Mueller–Hinton broth (for bacteria) or Sabouraud broth (for fungi) to obtain a density of 10^6^ CFU·mL^–1^. A series of twofold dilutions of the tested ILs were prepared on microplates in the concentration range from 1000 to 0.5 μg·mL^–1^. For this purpose, 100 μl of Mueller–Hinton or Sabouraud broth was introduced into the wells of sterile microplates, except for the first row, into which ionic liquid solutions with a concentration of 2000 μg·mL^–1^ were introduced. Subsequently, 100 μL of the bacterial suspension was introduced into all wells, obtaining a final inoculum density of 5 × 10^5^ CFU·mL^–1^. The negative control was the medium with the addition of ionic liquids, and the positive control was the culture of microorganisms without the addition of the inhibitory agent. The plates were incubated at the appropriate temperature for each microorganism (Table 3) for 24 h. The optical density of microbial growth was measured at a wavelength of 600 nm using a BioTek Epoch 2 microplate reader. The MIC value was defined as the concentration of the IL that inhibited the growth of the tested microorganism by at least 90%. The MBC/MFC value was determined by sport inoculation of 10 μL of microbial culture with the addition of ILs at a concentration equal to or higher than the determined MIC value (100% inhibition of bacterial growth based on spectrophotometric measurements).

**Table 3:** Conditions for the Cultivation of Microorganisms

microorganism	medium	temperature [°C]
*S. aureus* ATCC 33862	nutrient agar	37
*S. epidermidis* ATCC 12228	brain heart infusion agar	37
*E. faecalis* ATCC 19433	brain heart infusion agar	37
*B. subtilis* ATCC 11774	nutrient agar	37
*M. luteus* ATCC 4698	trypticase soy agar	30
*E. coli* ATCC 8739	nutrient agar	37
*P. aeruginosa* ATCC 9027	nutrient agar	37
*S. marcescens* ATCC 8100	trypticase soy agar	30
*P. vulgaris* ATCC 49132	brain heart infusion agar	37
*M. catarrhalis* ATCC 25238	trypticase soy agar	30
*C. albicans* ATCC 10231	Sabouraud agar with chloramphenicol	37
*R. rubra*	Sabouraud agar with chloramphenicol	30

## Supporting Information Available

^1^H NMR, ^13^C NMR, and FT-IR spectra of all compounds (Figures S1–S57), HPLC chromatograms for all compounds (Figures S58–S74), comparison of the NMR spectra of DSIL and its degradation products (Figures S75 and S76), surface tension isotherms for analyzed DSILs (Figure S77), elemental analysis of the compounds (Table S1), surface tension and contact angle of aqueous DSILs solutions (Tables S2–S4), and the calculated mean values of the MIC, MBC, and MFC (Tables S5–S8) (PDF)
