# Supplementary material for: Bifunctional Double-Salt Ionic Liquids Containing both 4-Chloro-2-Methylphenoxyacetate and l-Tryptophanate Anions with Herbicidal and Antimicrobial Activity
Source: ACS Omega. 2021 Dec 3;6(49):33779–91. doi: 10.1021/acsomega.1c05048 (PMC8679003; doi:10.1021/acsomega.1c05048)
Supplement: Supplementary file 1 — ao1c05048_si_001.pdf [file ao1c05048_si_001.pdf]

Supporting Information

Bifunctional double-salt ionic liquids containing both 4-chloro-2-methylphenoxyacetate and L-tryptophanate anions with herbicidal and antimicrobial activity

Daria Szymaniak[a], Kamil Ciarka[b], Katarzyna Marcinkowska[c],
Tadeusz Praczyk[c], Daniela Gwiazdowska[d], Katarzyna Marchwińska[d],
Filip Walkiewicz[a], *Juliusz Pernak[a]

[a] Faculty of Chemical Technology, Institute of Chemical Technology and Engineering, Poznan University of Technology, ul. Berdychowo 4, Poznań, 60-965, Poland

[b] PPC ADOB, ul. Kołodzieja 11, Poznań, 61-070, Poland

[c] Institute of Plant Protection, National Research Institute, ul. Węgorka 20, Poznań, 60-318, Poland

[d] Department of Natural Science and Quality Assurance, Institute of Quality Science, Poznan University of Economics and Business, al. Niepodległości 10, Poznań, 61-875, Poland

Table S1. Elemental analysis for the obtained DSILs

DSIL	Chemical formula	Molecular weight [g·mol^{-1}]	Calculated values [%]			Obtained values [%]		
			C	H	N	C	H	N
**1**	$C_{46}H_{77}ClN_4O_5$	801.60	68.93	9.68	6.99	68.55	9.37	6.67
**2**	$C_{50}H_{85}ClN_4O_5$	857.70	70.02	9.99	6.53	70.36	9.75	6.31
**3**	$C_{54}H_{93}ClN_4O_5$	913.81	70.98	10.26	6.13	70.62	10.56	6.23
**4**	$C_{58}H_{101}ClN_4O_5$	969.92	71.82	10.50	5.78	71.55	10.86	5.39
**5**	$C_{62}H_{109}ClN_4O_5$	1026.03	72.58	10.71	5.46	72.96	10.44	5.89
**6**	$C_{48}H_{81}ClN_4O_5$	829.65	69.49	9.84	6.75	69.12	10.15	7.09
**7**	$C_{52}H_{89}ClN_4O_5$	885.76	70.51	10.13	6.33	70.91	10.45	6.00
**8**	$C_{56}H_{97}ClN_4O_5$	941.87	71.41	10.38	5.95	71.85	10.06	5.61
**9**	$C_{60}H_{105}ClN_4O_5$	997.97	72.21	10.61	5.61	72.50	10.27	5.32
**10**	$C_{64}H_{113}ClN_4O_5$	1054.08	72.93	10.81	5.32	72.62	10.55	5.66
**11**	$C_{52}H_{89}ClN_4O_5$	885.76	70.51	10.13	6.33	70.85	10.35	6.68
**12**	$C_{56}H_{97}ClN_4O_5$	941.87	71.41	10.38	5.95	71.11	10.62	5.63
**13**	$C_{60}H_{105}ClN_4O_5$	997.97	72.21	10.61	5.61	72.52	10.25	5.97
**14**	$C_{64}H_{113}ClN_4O_5$	1054.08	72.93	10.81	5.32	72.62	10.45	5.68
**15**	$C_{68}H_{121}ClN_4O_5$	1110.19	73.57	10.99	5.05	73.96	10.64	5.37
**12a**	$C_{54}H_{94}Cl_2N_2O_6$	938.25	69.13	10.10	2.99	69.37	10.45	3.33
**12b**	$C_{58}H_{100}N_6O_4$	945.48	73.68	10.66	8.89	73.45	10.42	9.12
**12c**	$C_{56}H_{94}N_4O_4$	887.39	75.80	10.68	6.31	75.52	10.44	6.57
**12d**	$C_{60}H_{102}N_4O_4$	943.50	76.38	10.90	5.94	76.53	11.34	5.69

Table S2. Parameters of surface activity and contact angle of aqueous DSILs solutions at 25 °C

DSIL	CMC [mmol·L^{-1}]	γ_{cmc} [mN·m^{-1}]	pC_{20}	CA [°]
**1**	3.87	36.49	3.21	50
**2**	0.91	36.58	3.85	56
**3**	0.08	38.44	4.36	63
**4**	0.12	34.87	4.51	74
**5**	6.25	42.39	3.23	81
**6**	18.04	32.57	3.31	52
**7**	3.88	36.94	3.63	58
**8**	0.75	36.85	3.95	63
**9**	7.15	35.63	3.84	65
**10**	15.27	32.51	2.99	68
**11**	7.27	36.44	3.49	53
**12**	1.02	36.35	5.04	53
**13**	0.28	36.21	4.76	54
**14**	1.21	34.34	6.04	55
**15**	3.34	34.52	3.15	56

CMC – critical micelle concentration, γ_{CMC} – surface tension at CMC, pC_{20} – efficiency of surface adsorption at an air-water interface, CA – contact angle

Table S3. The surface tension of aqueous DSILs **1-10** solutions at 25 °C

DSIL	logC	γ [mN·m^{-1}]	DSIL	logC	γ [mN·m^{-1}]	DSIL	logC	γ [mN·m^{-1}]	DSIL	logC	γ [mN·m^{-1}]	DSIL	logC	γ [mN·m^{-1}]
**1**	-1.301029996	36.31	**2**	-1.301029996	36.70	**3**	-1.301029996	38.70	**4**	-1.301029996	35.70	**5**	-1.301029996	42.70
	-1.110106052	36.30		-1.092532043	36.60		-1.09242433	38.60		-1.128204178	35.60		-1.114281258	42.60
	-1.411136047	36.28		-1.393562039	36.68		-1.393454325	38.68		-1.429234174	35.68		-1.415311254	42.68
	-1.712166043	36.33		-1.694592034	36.73		-1.694484321	38.73		-1.730264169	35.73		-1.71634125	42.53
	-2.013196039	36.33		-1.99562203	36.85		-1.995514317	38.37		-2.031294165	35.03		-2.017371245	42.41
	-2.314226034	36.56		-2.296652026	36.80		-2.296544312	38.95		-2.332324161	35.00		-2.318401241	45.00
	-2.61525603	39.76		-2.597682021	36.75		-2.597574308	38.61		-2.633354156	35.06		-2.619431237	46.18
	-2.916286026	45.92		-2.898712017	36.28		-2.898604304	38.39		-2.934384152	35.00		-2.920461232	49.00
	-3.217316021	53.60		-3.199742013	39.16		-3.199634299	38.23		-3.235414148	34.58		-3.221491228	51.00
	-3.518346017	58.32		-3.500772008	45.75		-3.500664295	38.67		-3.536444143	35.00		-3.522521224	54.00
	-3.819376013	64.02		-3.801802004	49.77		-3.801694291	38.50		-3.837474139	35.53		-3.823551219	56.00
	-4.120406008	69.00		-4.102832	59.13		-4.102724286	40.17		-4.138504135	40.46		-4.124581215	60.00
	-4.421436004	70.40		-4.403861995	62.07		-4.403754282	51.86		-4.43953413	51.25		-4.425611211	64.00
	-4.722466	70.30		-4.704891991	67.66		-4.704784278	70.35		-4.740564126	58.18		-4.726641206	67.00
**6**	-1.301029996	32.70	**7**	-1.301029996	36.51	**8**	-1.301029996	36.56	**9**	-1.301029996	35.56	**10**	-1.301029996	32.56
	-1.088337144	32.60		-1.096947095	36.50		-1.093374618	36.60		-1.13985013	35.60		-1.079501907	32.60
	-1.38936714	32.68		-1.397977091	36.48		-1.394404614	36.62		-1.440880126	35.62		-1.380531903	32.59
	-1.690397136	32.53		-1.699007087	36.53		-1.69543461	36.53		-1.741910122	35.53		-1.681561899	32.53
	-1.991427131	32.52		-2.000037082	36.70		-1.996464605	36.17		-2.042940117	35.00		-1.982591894	32.48
	-2.292457127	38.34		-2.301067078	37.13		-2.297494601	36.49		-2.343970113	35.88		-2.28362189	39.00
	-2.593487122	43.02		-2.602097074	37.00		-2.598524597	37.09		-2.645000109	35.90		-2.584651886	45.21
	-2.894517118	48.55		-2.903127069	37.10		-2.899554592	36.88		-2.946030104	43.00		-2.885681881	52.00
	-3.195547114	50.72		-3.204157065	45.57		-3.200584588	39.58		-3.2470601	47.00		-3.186711877	56.00
	-3.496577109	54.12		-3.505187061	50.01		-3.501614584	42.42		-3.548090096	49.00		-3.487741873	60.00
	-3.797607105	57.36		-3.806217056	55.40		-3.802644579	48.22		-3.849120091	52.00		-3.788771868	65.00
	-4.098637101	67.91		-4.107247052	59.00		-4.103674575	55.10		-4.150150087	55.00		-4.089801864	69.86
	-4.399667096	67.81		-4.408277048	62.00		-4.404704571	61.93		-4.451180083	58.00		-4.39083186	71.00
	-4.700697092	68.00		-4.709307043	71.03		-4.705734566	65.40		-4.752210078	66.16		-4.691861855	70.90

γ – surface tension

Table S4. The surface tension of aqueous DSILs **11-15** solutions at 25 °C

DSIL	logC	γ [mN·m^{-1}]	DSIL	logC	γ [mN·m^{-1}]	DSIL	logC	γ [mN·m^{-1}]	DSIL	logC	γ [mN·m^{-1}]	DSIL	logC	γ [mN·m^{-1}]
**11**	-1.301029996	36.31	**12**	-1.301029996	36.31	**13**	-1.301029996	36.31	**14**	-1.301029996	34.56	**15**	-1.301029996	34.51
	-1.11490935	36.30		-1.10995017	36.30		-1.07633412	36.30		-1.103920363	34.60		-1.097144788	34.50
	-1.415939346	36.28		-1.410980166	36.28		-1.377364116	36.28		-1.404950359	34.59		-1.398174784	34.48
	-1.716969342	36.33		-1.712010162	36.33		-1.678394111	36.33		-1.705980354	34.53		-1.699204779	34.53
	-2.017999337	36.33		-2.013040157	36.43		-1.979424107	36.23		-2.00701035	34.30		-2.000234775	34.58
	-2.319029333	36.56		-2.314070153	36.54		-2.280454103	36.12		-2.308040346	34.40		-2.301264771	34.45
	-2.620059329	41.00		-2.615100149	36.26		-2.581484098	36.10		-2.609070341	34.44		-2.602294766	34.53
	-2.921089324	45.63		-2.916130144	36.25		-2.882514094	36.38		-2.910100337	34.38		-2.903324762	46.23
	-3.22211932	50.00		-3.21716014	38.40		-3.18354409	36.25		-3.211130333	36.00		-3.204354758	52.48
	-3.523149316	53.00		-3.518190136	40.00		-3.484574085	36.22		-3.512160328	38.00		-3.505384753	60.64
	-3.824179311	55.68		-3.819220131	42.51		-3.785604081	39.29		-3.813190324	39.00		-3.806414749	69.41
	-4.125209307	58.65		-4.120250127	45.23		-4.086634077	43.00		-4.11422032	41.00		-4.107444745	69.33
	-4.426239303	66.25		-4.421280123	49.77		-4.387664072	47.22		-4.415250315	43.00		-4.40847474	70.33
	-4.727269298	70.00		-4.722310118	58.90		-4.688694068	71.03		-4.716280311	48.18		-4.709504736	72.76

γ – surface tension

Table S5. MIC (μg·mL^{-1}) and MBC or MFC (μg·mL^{-1}) values recorded for DSILs **1-5**

Microorganism	MIC/ MBC /MFC	1		2		3		4		5	
		29.09.20	28.10.20	29.09.20	28.10.20	29.09.20	28.10.20	29.09.20	28.10.20	29.09.20	28.10.20
**Gram-positive bacteria**											
Staphylococcus aureus ATCC 33862	MIC	125	125	4	4	4	4	31	31	250	500
	MBC	250	250	8	8	4	8	31	31	500	1000
Staphylococcus epidermidis ATCC 12228	MIC	31	31	1	< 0,5	< 0,5	< 0,5	4	4	8	8
	MBC	31	31	1	< 0,5	< 0,5	< 0,5	4	4	8	8
Bacillus subtilis ATCC 11774	MIC	62	1000	1	62	1	4	4	31	8	62
	MBC	62	1000	1	62	2	4	4	31	8	62
Enterococcus faecalis ATCC 19433	MIC	125	250	2	1	0,5	< 0,5	8	4	250	250
	MBC	125	250	2	1	1	< 0,5	8	4	250	500
Micrococcus luteus ATCC 4698	MIC	0,5	2	< 0,5	< 0,5	< 0,5	< 0,5	< 0,5	1	2	4
	MBC	1	4	< 0,5	< 0,5	< 0,5	< 0,5	< 0,5	1	4	4
**Gram-negative bacteria**											
Pseudomonas aeruginosa ATCC 9027	MIC	1000	1000	31	31	8	8	> 1000	> 1000	125	125
	MBC	1000	1000	31	31	8	8	> 1000	> 1000	125	125
Serratia marcescens ATCC 8100	MIC	> 1000	> 1000	250	> 1000	4	32	> 1000	> 1000	> 1000	> 1000
	MBC	> 1000	> 1000	250	> 1000	8	32	> 1000	> 1000	> 1000	> 1000
Proteus vulgaris ATCC 49132	MIC	500	1000	16	16	4	4	31	31	125	125
	MBC	500	1000	16	16	4	4	31	31	125	125
Moraxella catarrhalis ATCC 25238	MIC	4	4	< 0,5	< 0,5	< 0,5	< 0,5	< 0,5	< 0,5	4	4
	MBC	4	4	< 0,5	< 0,5	< 0,5	< 0,5	< 0,5	< 0,5	4	4
Escherichia coli ATCC 8739	MIC	250	250	4	2	2	4	16	16	62	62
	MBC	250	250	8	2	2	4	16	16	62	62
**Yeasts**											
Rhodotorula rubra	MIC	1000	500	8	8	2	< 0,5	4	2	16	16
	MFC	> 1000	500	8	8	4	< 0,5	4	2	31	16
Candida albicans ATCC 10231	MIC	500	1000	16	31	2	2	16	16	500	500
	MFC	500	1000	16	31	2	2	16	16	500	500

MIC – minimum inhibitory concentration, MBC –minimum bactericidal concentration, MFC – minimum fungicidal concentration

Table S6. MIC (µg·mL^{-1}) and MBC or MFC (µg·mL^{-1}) values recorded for DSILs **6-10**

Microorganism	MIC/ MBC /MFC	6		7		8		9		10	
		29.09.20	28.10.20	29.09.20	28.10.20	29.09.20	28.10.20	29.09.20	28.10.20	29.09.20	28.10.20
**Gram-positive bacteria**											
Staphylococcus aureus ATCC 33862	MIC	62	125	4	4	4	4	31	31	125	250
	MBC	125	125	4	4	4	4	31	31	125	250
Staphylococcus epidermidis ATCC 12228	MIC	8	8	< 0,5	< 0,5	< 0,5	0,5	4	2	125	125
	MBC	16	8	< 0,5	< 0,5	< 0,5	1	4	2	125	125
Bacillus subtilis ATCC 11774	MIC	16	1000	1	31	1	8	4	31	125	125
	MBC	16	1000	1	31	2	8	8	31	125	125
Enterococcus faecalis ATCC 19433	MIC	31	62	1	1	2	2	8	8	500	500
	MBC	31	62	1	1	2	2	8	8	1000	500
Micrococcus luteus ATCC 4698	MIC	< 0,5	2	< 0,5	< 0,5	< 0,5	< 0,5	0,5	1	2	2
	MBC	< 0,5	4	< 0,5	< 0,5	< 0,5	< 0,5	1	1	2	8
**Gram-negative bacteria**											
Pseudomonas aeruginosa ATCC 9027	MIC	500	500	16	16	8	4	62	1000	125	125
	MBC	500	500	16	16	8	4	62	>1000	125	250
Serratia marcescens ATCC 8100	MIC	1000	> 1000	250	> 1000	16	62	> 1000	> 1000	1000	500
	MBC	> 1000	> 1000	500	> 1000	16	62	> 1000	> 1000	1000	1000
Proteus vulgaris ATCC 49132	MIC	125	500	4	8	4	4	32	16	125	250
	MBC	125	500	4	8	4	4	32	16	250	250
Moraxella catarrhalis ATCC 25238	MIC	1	2	< 0,5	< 0,5	< 0,5	< 0,5	< 0,5	< 0,5	500	500
	MBC	1	2	< 0,5	< 0,5	< 0,5	< 0,5	< 0,5	< 0,5	500	500
Escherichia coli ATCC 8739	MIC	16	16	1	< 0,5	2	2	16	16	125	125
	MBC	16	16	1	< 0,5	2	2	16	16	125	250
**Yeasts**											
Rhodotorula rubra	MIC	250	250	4	2	2	1	4	4	250	250
	MFC	250	250	4	2	2	1	4	4	250	250
Candida albicans ATCC 10231	MIC	125	250	4	8	2	2	16	16	500	500
	MFC	125	250	4	8	2	2	16	16	500	500

MIC – minimum inhibitory concentration, MBC –minimum bactericidal concentration, MFC – minimum fungicidal concentration

Table S7. MIC (µg·mL^{-1}) and MBC or MFC (µg·mL^{-1}) values recorded for DSILs **11-15**

Microorganism	MIC/ MBC /MFC	11		12		13		14		15	
		29.09.20	28.10.20	29.09.20	28.10.20	29.09.20	28.10.20	29.09.20	28.10.20	29.09.20	28.10.20
**Gram-positive bacteria**											
Staphylococcus aureus ATCC 33862	MIC	8	8	1	1	4	4	4	4	62	>1000
	MBC	8	8	1	1	8	8	4	4	125	>1000
Staphylococcus epidermidis ATCC 12228	MIC	< 0,5	< 0,5	< 0,5	< 0,5	1	2	4	4	8	8
	MBC	< 0,5	< 0,5	< 0,5	< 0,5	2	2	4	4	8	8
Bacillus subtilis ATCC 11774	MIC	0,5	32	0,5	1	4	8	8	16	8	>1000
	MBC	1	32	1	1	4	8	8	16	16	>1000
Enterococcus faecalis ATCC 19433	MIC	1	1	0,5	< 0,5	2	1	8	4	8	> 1000
	MBC	1	1	1	< 0,5	2	1	8	4	16	> 1000
Micrococcus luteus ATCC 4698	MIC	< 0,5	< 0,5	< 0,5	< 0,5	< 0,5	< 0,5	1	1	2	4
	MBC	< 0,5	< 0,5	< 0,5	< 0,5	< 0,5	< 0,5	1	1	2	4
**Gram-negative bacteria**											
Pseudomonas aeruginosa ATCC 9027	MIC	62	62	4	4	8	8	62	62	> 1000	> 1000
	MBC	62	62	4	4	8	16	62	62	> 1000	> 1000
Serratia marcescens ATCC 8100	MIC	250	500	4	16	125	125	> 1000	> 1000	> 1000	> 1000
	MBC	250	500	4	16	125	125	> 1000	> 1000	> 1000	> 1000
Proteus vulgaris ATCC 49132	MIC	16	31	1	2	4	4	62	62	125	> 1000
	MBC	16	31	1	2	4	4	62	62	125	> 1000
Moraxella catarrhalis ATCC 25238	MIC	< 0,5	< 0,5	< 0,5	< 0,5	< 0,5	< 0,5	1	< 0,5	1	1
	MBC	< 0,5	< 0,5	< 0,5	< 0,5	< 0,5	< 0,5	1	< 0,5	1	1
Escherichia coli ATCC 8739	MIC	< 0,5	1	1	1	4	4	31	31	31	> 1000
	MBC	< 0,5	1	1	2	4	4	62	31	31	> 1000
**Yeasts**											
Rhodotorula rubra	MIC	8	4	< 0,5	< 0,5	1	< 0,5	8	4	31	8
	MFC	8	4	< 0,5	< 0,5	2	< 0,5	8	4	31	8
Candida albicans ATCC 10231	MIC	2	4	< 0,5	< 0,5	2	2	62	62	16	31
	MFC	2	4	< 0,5	< 0,5	2	2	62	62	31	31

MIC – minimum inhibitory concentration, MBC –minimum bactericidal concentration, MFC – minimum fungicidal concentration

Table S8. MIC ($\mu g\cdot mL^{-1}$) and MBC or MFC ($\mu g\cdot mL^{-1}$) values recorded for DSILs **12a**-**12d**

Microorganism	MIC/ MBC /MFC	12a		12b		12c		12d		[DDA][Cl]	[Ba][Cl]
		29.09.20	28.10.20	29.09.20	28.10.20	29.09.20	28.10.20	29.09.20	28.10.20		
**Gram-positive bacteria**											
Staphylococcus aureus ATCC 33862	MIC	1	1	1	1	1	1	1	1	< 0,5	< 0,5
	MBC	1	1	1	1	1	1	2	1	0,5	1
Staphylococcus epidermidis ATCC 12228	MIC	< 0,5	< 0,5	< 0,5	< 0,5	< 0,5	< 0,5	< 0,5	< 0,5	< 0,5	< 0,5
	MBC	< 0,5	< 0,5	< 0,5	< 0,5	< 0,5	< 0,5	< 0,5	< 0,5	< 0,5	< 0,5
Bacillus subtilis ATCC 11774	MIC	< 0,5	1	< 0,5	1	< 0,5	1	0,5	2	0,5	0,5
	MBC	< 0,5	1	< 0,5	1	< 0,5	1	1	2	1	1
Enterococcus faecalis ATCC 19433	MIC	< 0,5	< 0,5	< 0,5	< 0,5	< 0,5	< 0,5	< 0,5	< 0,5	< 0,5	0,5
	MBC	< 0,5	< 0,5	< 0,5	< 0,5	< 0,5	< 0,5	< 0,5	< 0,5	< 0,5	1
Micrococcus luteus ATCC 4698	MIC	< 0,5	< 0,5	< 0,5	< 0,5	< 0,5	< 0,5	< 0,5	< 0,5	< 0,5	< 0,5
	MBC	< 0,5	< 0,5	< 0,5	< 0,5	< 0,5	< 0,5	< 0,5	< 0,5	< 0,5	< 0,5
**Gram-negative bacteria**											
Pseudomonas aeruginosa ATCC 9027	MIC	31	2	31	2	31	2	31	4	8	16
	MBC	31	2	31	2	31	2	31	4	8	31
Serratia marcescens ATCC 8100	MIC	8	4	8	4	16	4	8	8	4	4
	MBC	8	4	16	4	16	4	16	8	8	8
Proteus vulgaris ATCC 49132	MIC	2	2	1	2	1	2	2	2	4	8
	MBC	2	2	1	2	1	2	2	2	4	16
Moraxella catarrhalis ATCC 25238	MIC	< 0,5	< 0,5	< 0,5	< 0,5	< 0,5	< 0,5	< 0,5	< 0,5	< 0,5	0,5
	MBC	< 0,5	< 0,5	< 0,5	< 0,5	< 0,5	< 0,5	< 0,5	< 0,5	< 0,5	1
Escherichia coli ATCC 8739	MIC	< 0,5	< 0,5	< 0,5	< 0,5	< 0,5	< 0,5	< 0,5	1	1	4
	MBC	< 0,5	< 0,5	< 0,5	< 0,5	< 0,5	< 0,5	< 0,5	1	1	8
**Yeasts**											
Rhodotorula rubra	MIC	< 0,5	< 0,5	< 0,5	< 0,5	< 0,5	< 0,5	< 0,5	< 0,5	1	4
	MFC	< 0,5	< 0,5	< 0,5	< 0,5	< 0,5	< 0,5	< 0,5	< 0,5	2	8
Candida albicans ATCC 10231	MIC	< 0,5	< 0,5	< 0,5	< 0,5	< 0,5	< 0,5	< 0,5	< 0,5	2	4
	MFC	< 0,5	< 0,5	< 0,5	< 0,5	< 0,5	< 0,5	< 0,5	< 0,5	2	4

MIC – minimum inhibitory concentration, MBC –minimum bactericidal concentration, MFC – minimum fungicidal concentration

File Name						Frequency (MHz)	399.91
Nucleus	1H	Number of Transients	64	Original Points Count	24814	Points Count	32768
Pulse Sequence	s2pul	Receiver Gain	12.00	Solvent	METHANOL-d4	Spectrum Offset (Hz)	1231.8997
Spectrum Type	STANDARD	Sweep Width (Hz)	3544.84	Temperature (degree C)	AMBIENT TEMPERATURE		

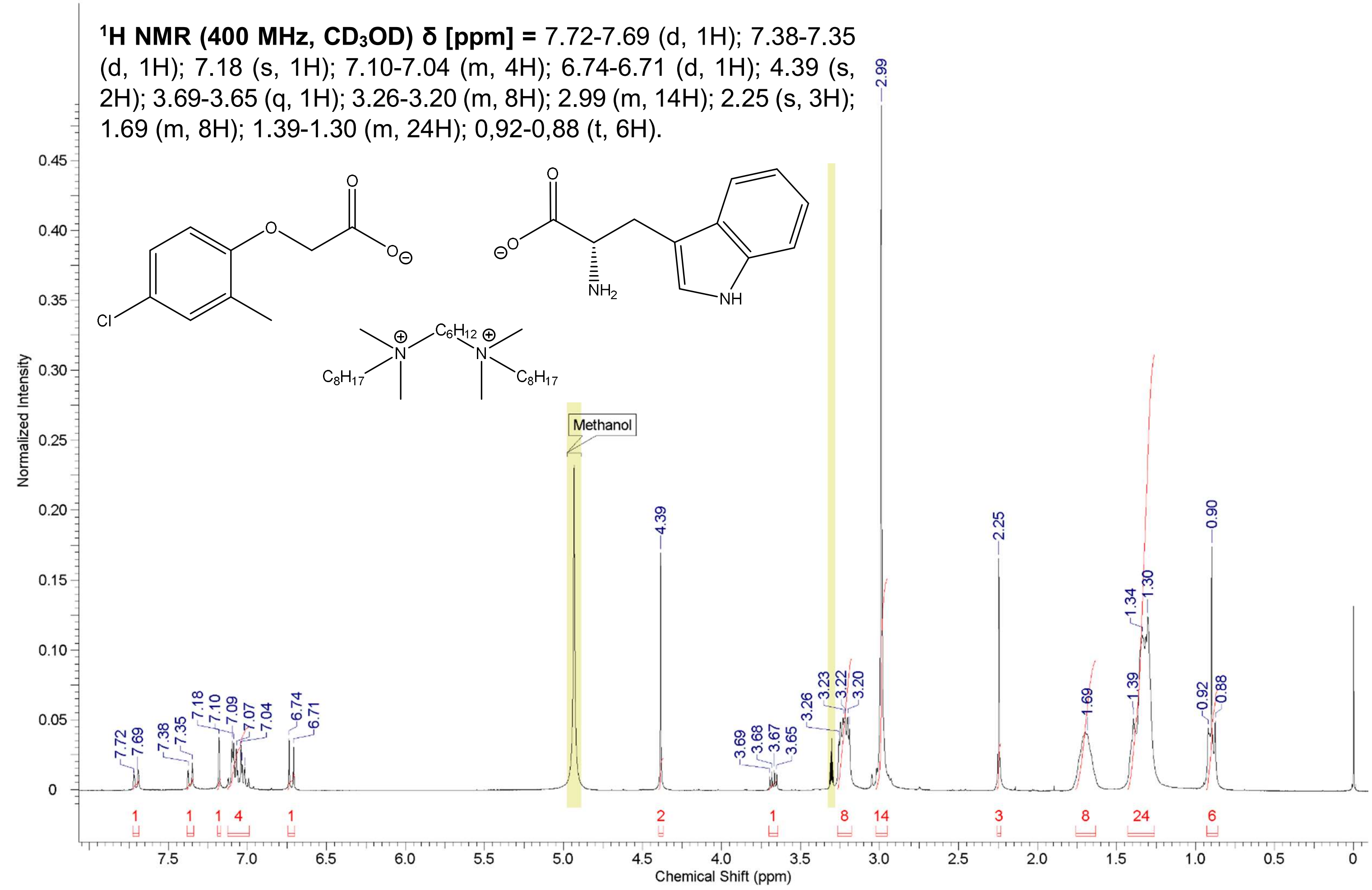

Figure S1. ^{1}H NMR spectrum of L-tryptophanate 4-chloro-2-methylphenoxyacetate hexamethylene-1,6-bis(octadimethylammonium) (**1**)

File Name						Frequency (MHz)	100.57
Nucleus	13C	Number of Transients	1932	Original Points Count	27451	Points Count	32768
Pulse Sequence	s2pul	Receiver Gain	32.00	Solvent	METHANOL-d4	Spectrum Offset (Hz)	8408.4453
Spectrum Type	STANDARD	Sweep Width (Hz)	19607.84	Temperature (degree C)	AMBIENT TEMPERATURE		

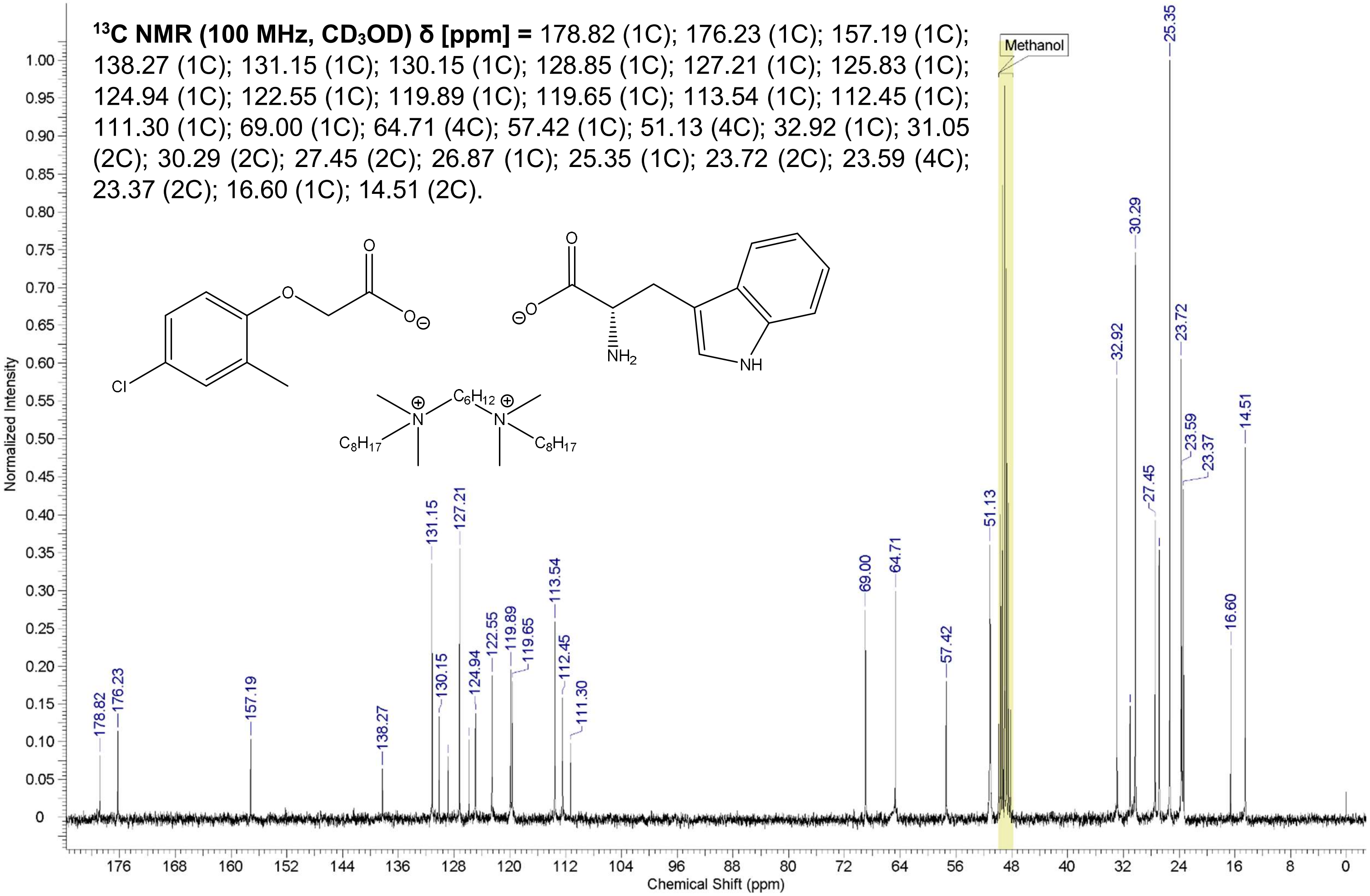

Figure S2. ^{13}C NMR spectrum of L-tryptophanate 4-chloro-2-methylphenoxyacetate hexamethylene-1,6-bis(octadimethylammonium) (**1**)

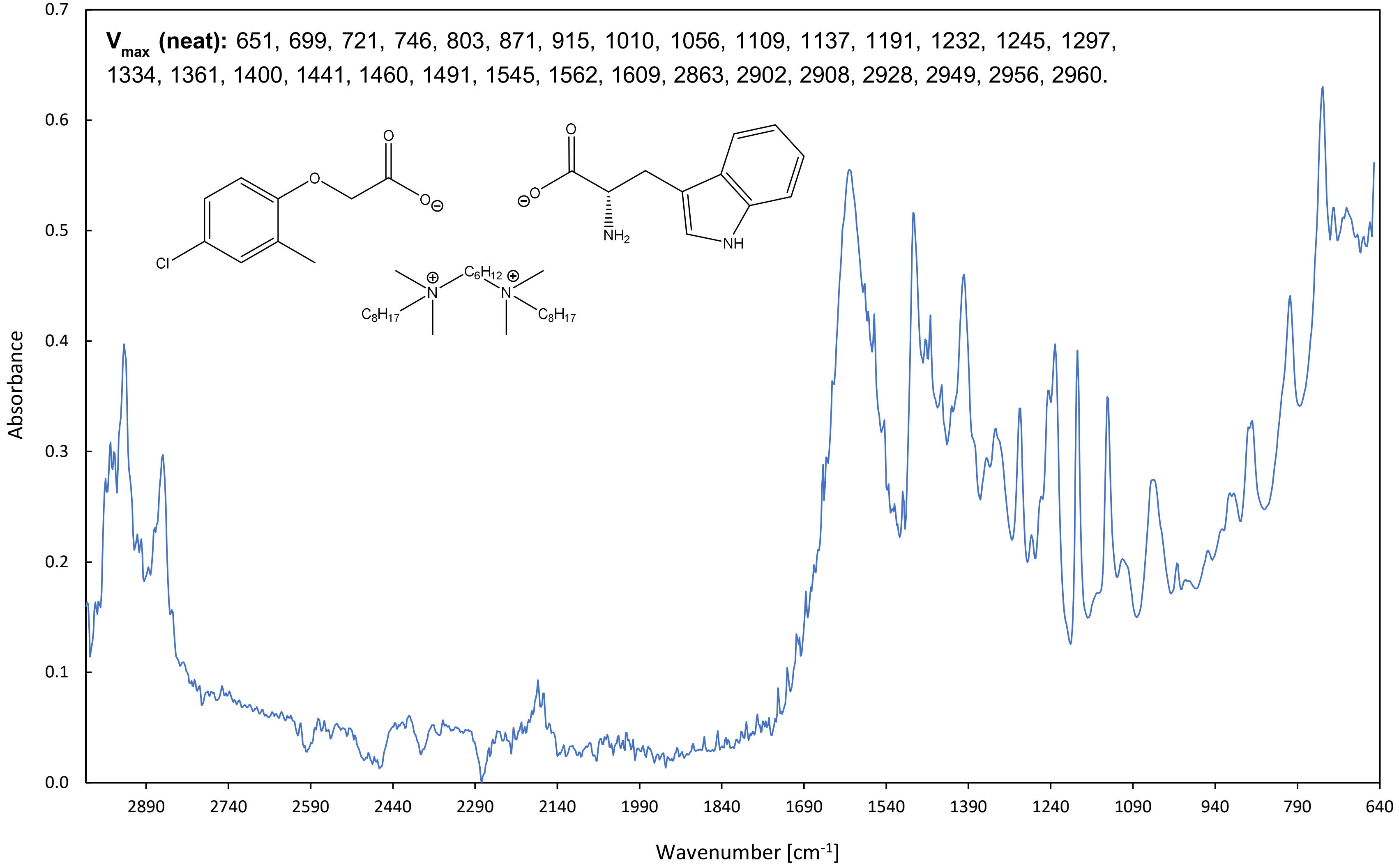

Figure S3. FT-IR spectrum of L-tryptophanate 4-chloro-2-methylphenoxyacetate hexamethylene-1,6-bis(octadimethylammonium) (**1**)

File Name						***Frequency (MHz)***	399.91
Nucleus	1H	***Number of Transients***	44	***Original Points Count***	29761	***Points Count***	32768
Pulse Sequence	s2pul	***Receiver Gain***	18.00	***Solvent***	METHANOL-d4	***Spectrum Offset (Hz)***	2620.3955
Spectrum Type	STANDARD	***Sweep Width (Hz)***	5952.38	***Temperature (degree C)***	AMBIENT TEMPERATURE		

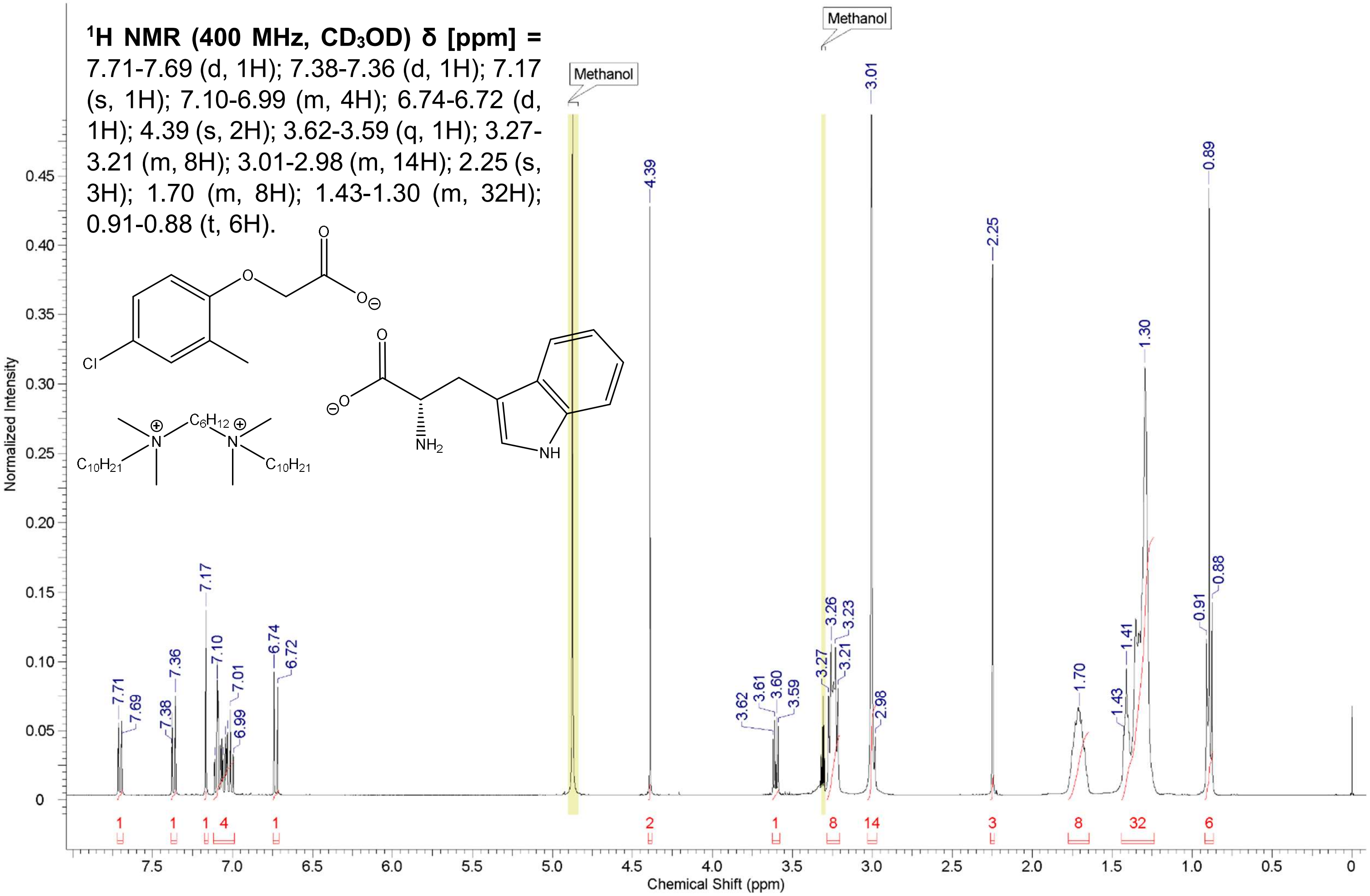

Figure S4. ^{1}H NMR spectrum of L-tryptophanate 4-chloro-2-methylphenoxyacetate hexamethylene-1,6-bis(decyldimethylammonium) (**2**)

File Name						Frequency (MHz)	100.57
Nucleus	13C	Number of Transients	1108	Original Points Count	32768	Points Count	32768
Pulse Sequence	s2pul	Receiver Gain	52.00	Solvent	METHANOL-d4	Spectrum Offset (Hz)	10180.9648
Spectrum Type	STANDARD	Sweep Width (Hz)	22321.43	Temperature (degree C)	AMBIENT TEMPERATURE		

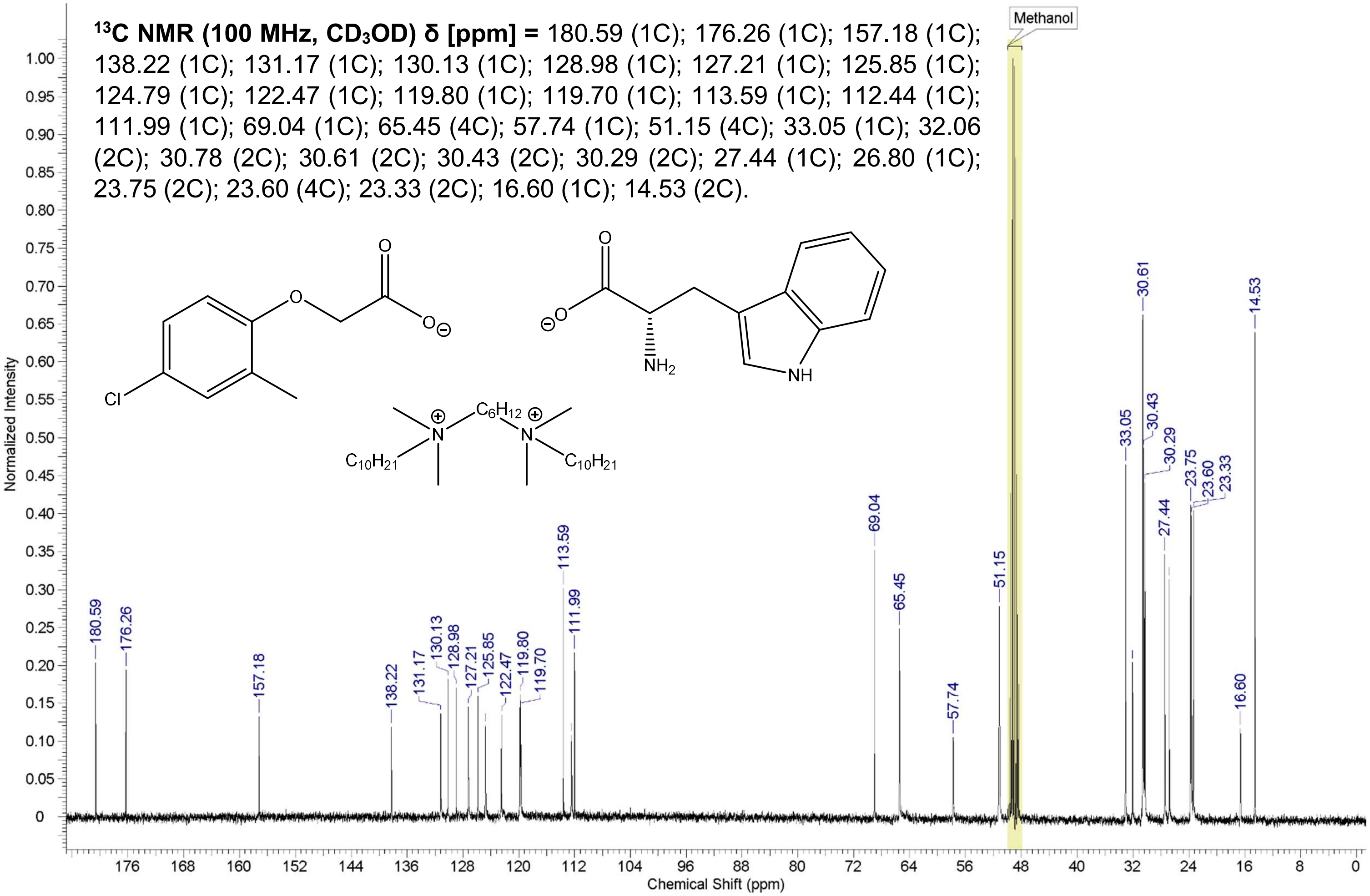

Figure S5. ^{13}C NMR spectrum of L-tryptophanate 4-chloro-2-methylphenoxyacetate hexamethylene-1,6-bis(decyldimethylammonium) (**2**)

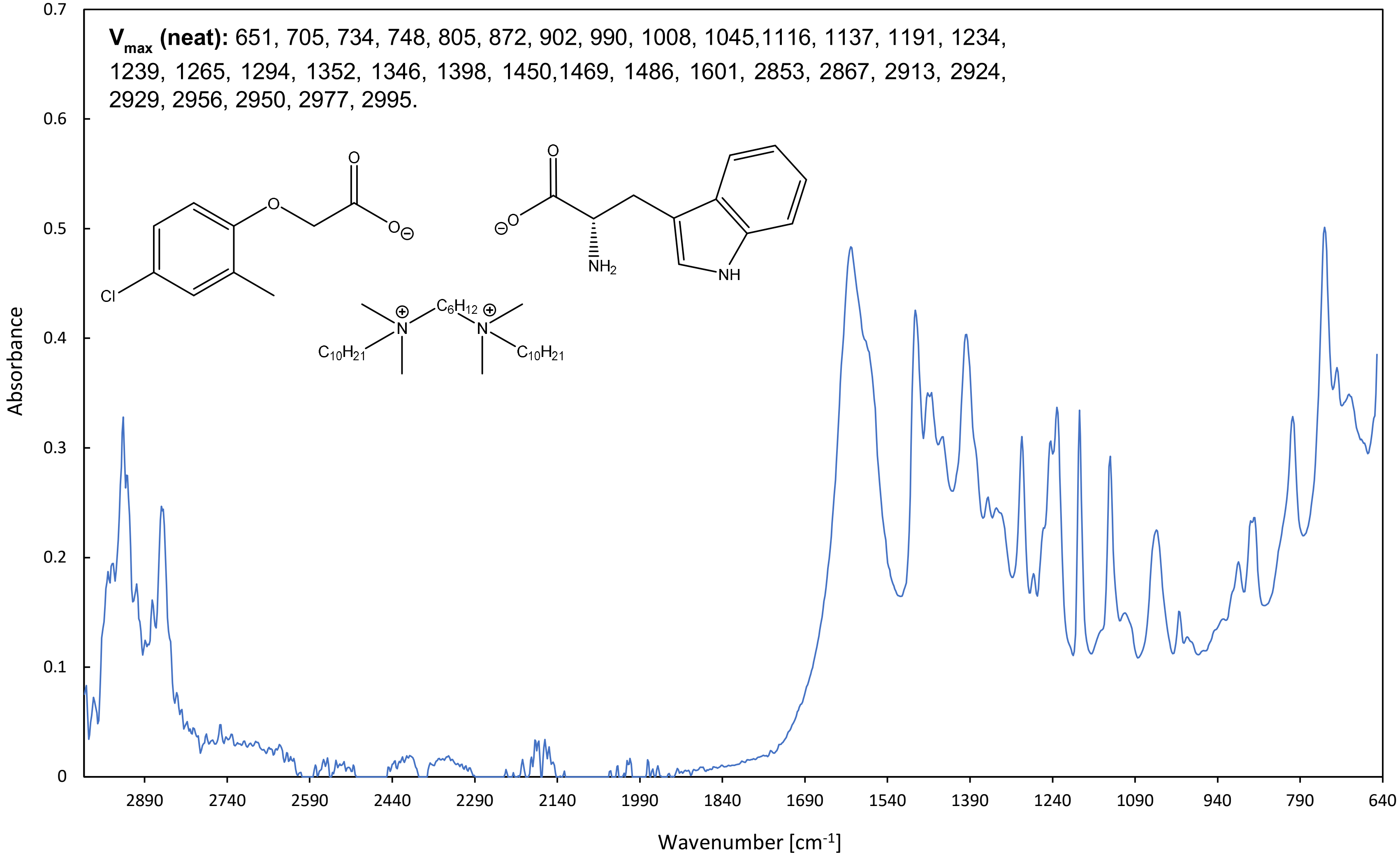

Figure S6. FT-IR spectrum of L-tryptophanate 4-chloro-2-methylphenoxyacetate hexamethylene-1,6-bis(decyldimethylammonium) (**2**)

File Name						Frequency (MHz)	300.41
Nucleus	1H	Number of Transients	64	Original Points Count	24814	Points Count	32768
Pulse Sequence	s2pul	Receiver Gain	12.00	Solvent	METHANOL-d4	Spectrum Offset (Hz)	1231.6826
Spectrum Type	STANDARD	Sweep Width (Hz)	3544.84	Temperature (degree C)	AMBIENT TEMPERATURE		

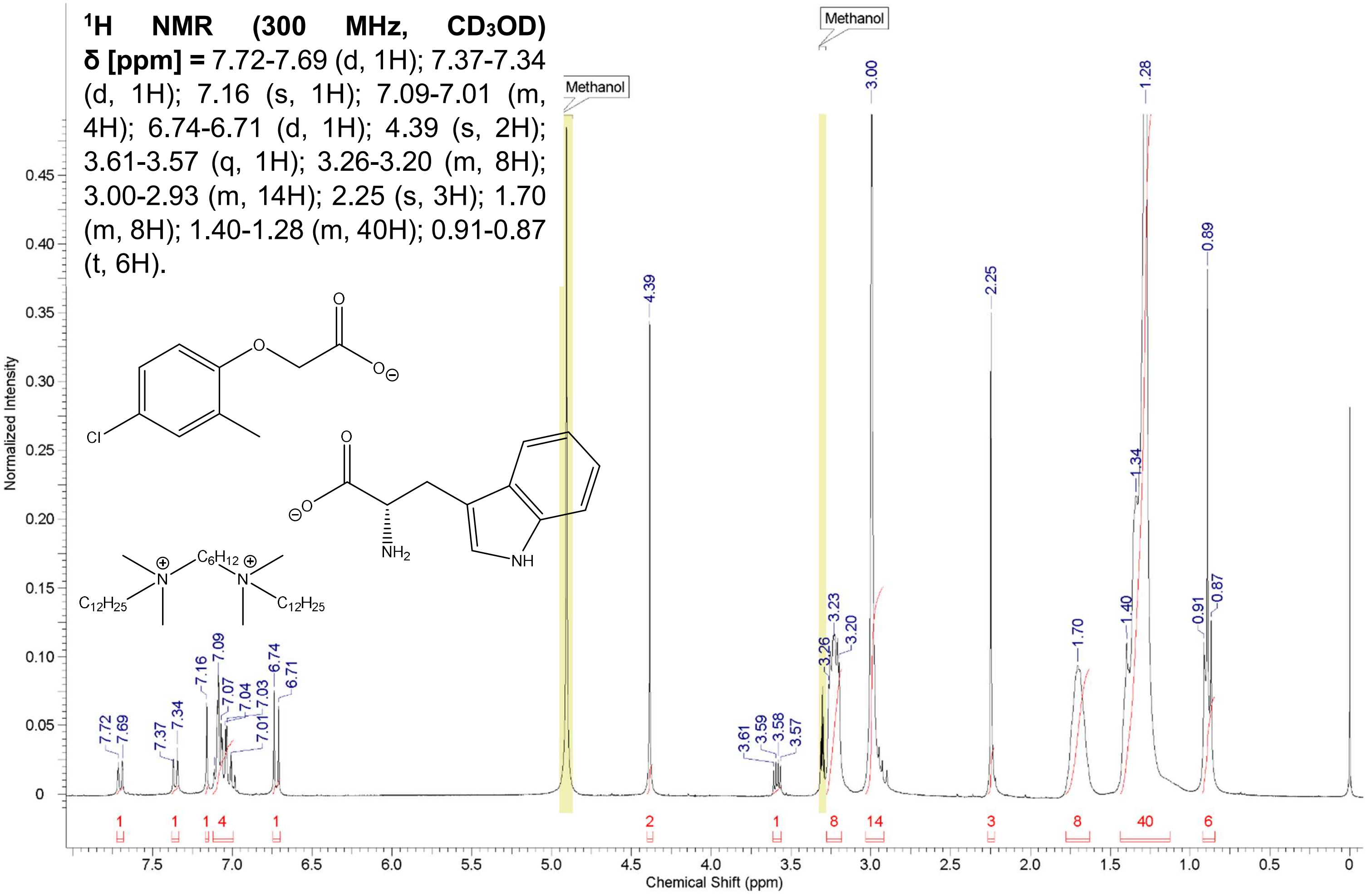

Figure S7. ^{1}H NMR spectrum of L-tryptophanate 4-chloro-2-methylphenoxyacetate hexamethylene-1,6-bis(dodecyldimethylammonium) (**3**)

File Name						***Frequency (MHz)***	75.55
Nucleus	13C	***Number of Transients***	1664	***Original Points Count***	27451	***Points Count***	32768
Pulse Sequence	s2pul	***Receiver Gain***	32.00	***Solvent***	METHANOL-d4	***Spectrum Offset (Hz)***	8408.4463
Spectrum Type	STANDARD	***Sweep Width (Hz)***	19607.84	***Temperature (degree C)***	AMBIENT TEMPERATURE		

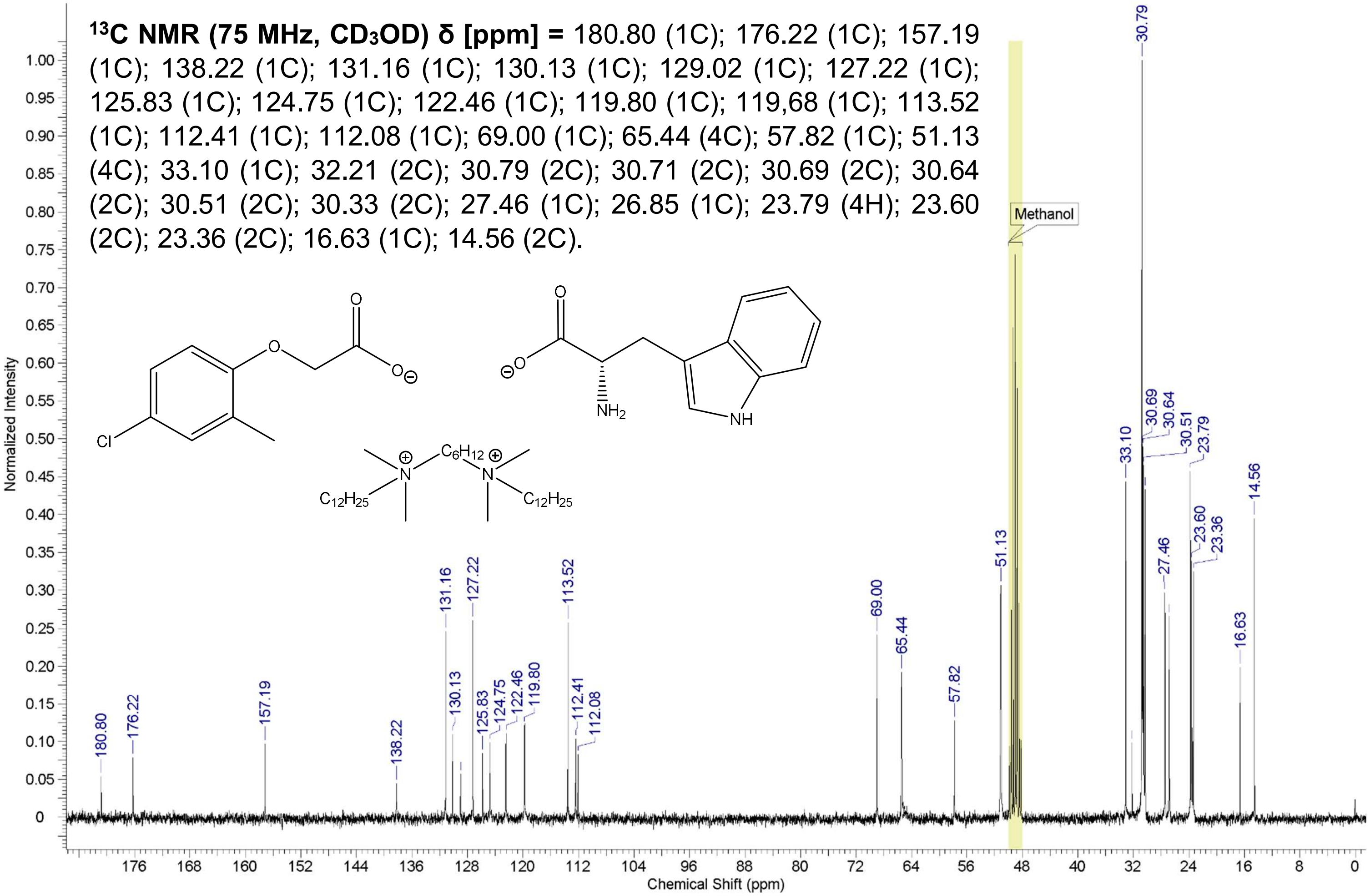

Figure S8. ^{13}C NMR spectrum of L-tryptophanate 4-chloro-2-methylphenoxyacetate hexamethylene-1,6-bis(dodecyldimethylammonium) (**3**)

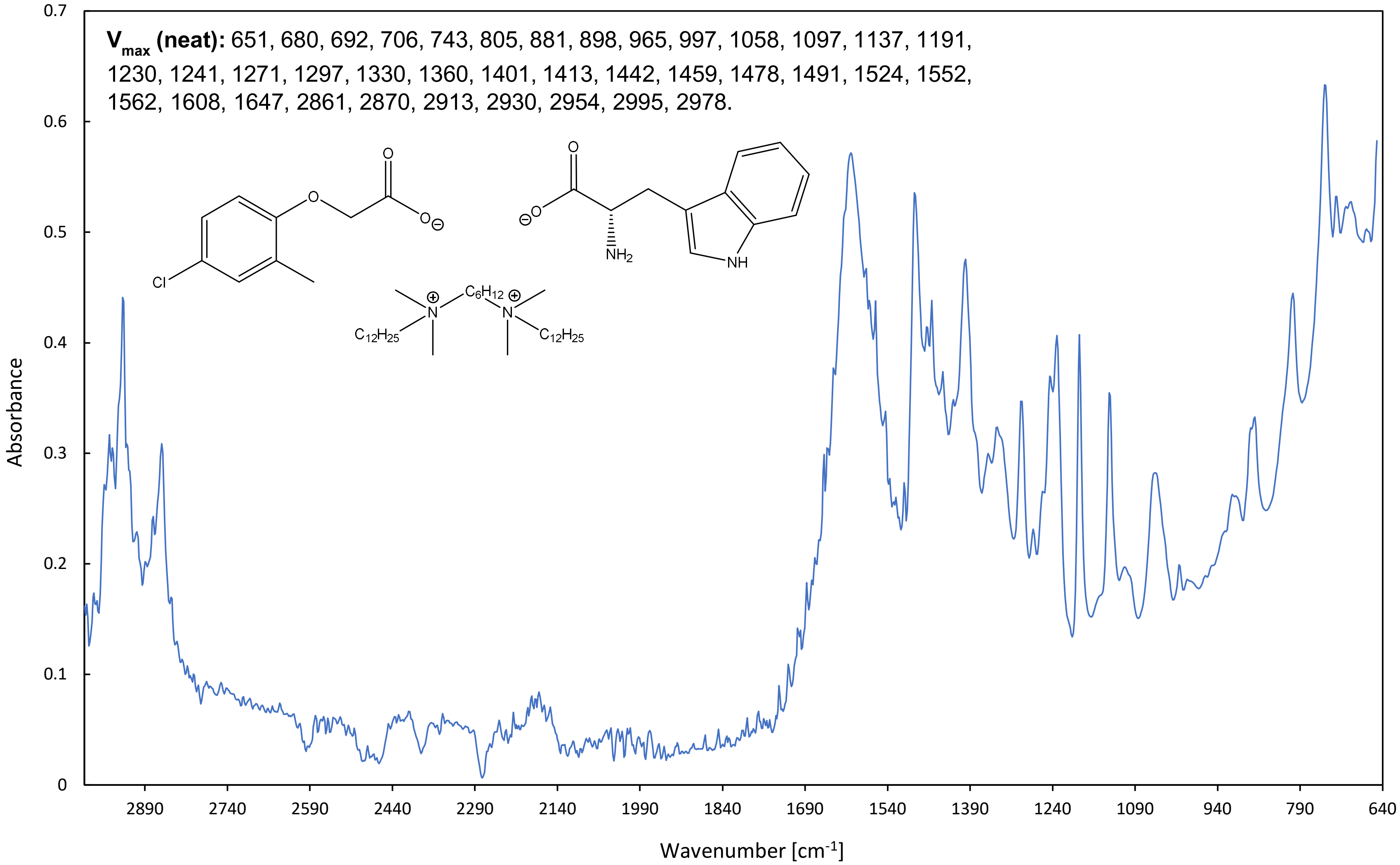

Figure S9. FT-IR spectrum of L-tryptophanate 4-chloro-2-methylphenoxyacetate hexamethylene-1,6-bis(dodecyldimethylammonium) (**3**)

File Name						Frequency (MHz)	399.91
Nucleus	1H	Number of Transients	40	Original Points Count	29761	Points Count	32768
Pulse Sequence	s2pul	Receiver Gain	52.00	Solvent	METHANOL-d4	Spectrum Offset (Hz)	2619.9705
Spectrum Type	STANDARD	Sweep Width (Hz)	5952.38	Temperature (degree C)	AMBIENT TEMPERATURE		

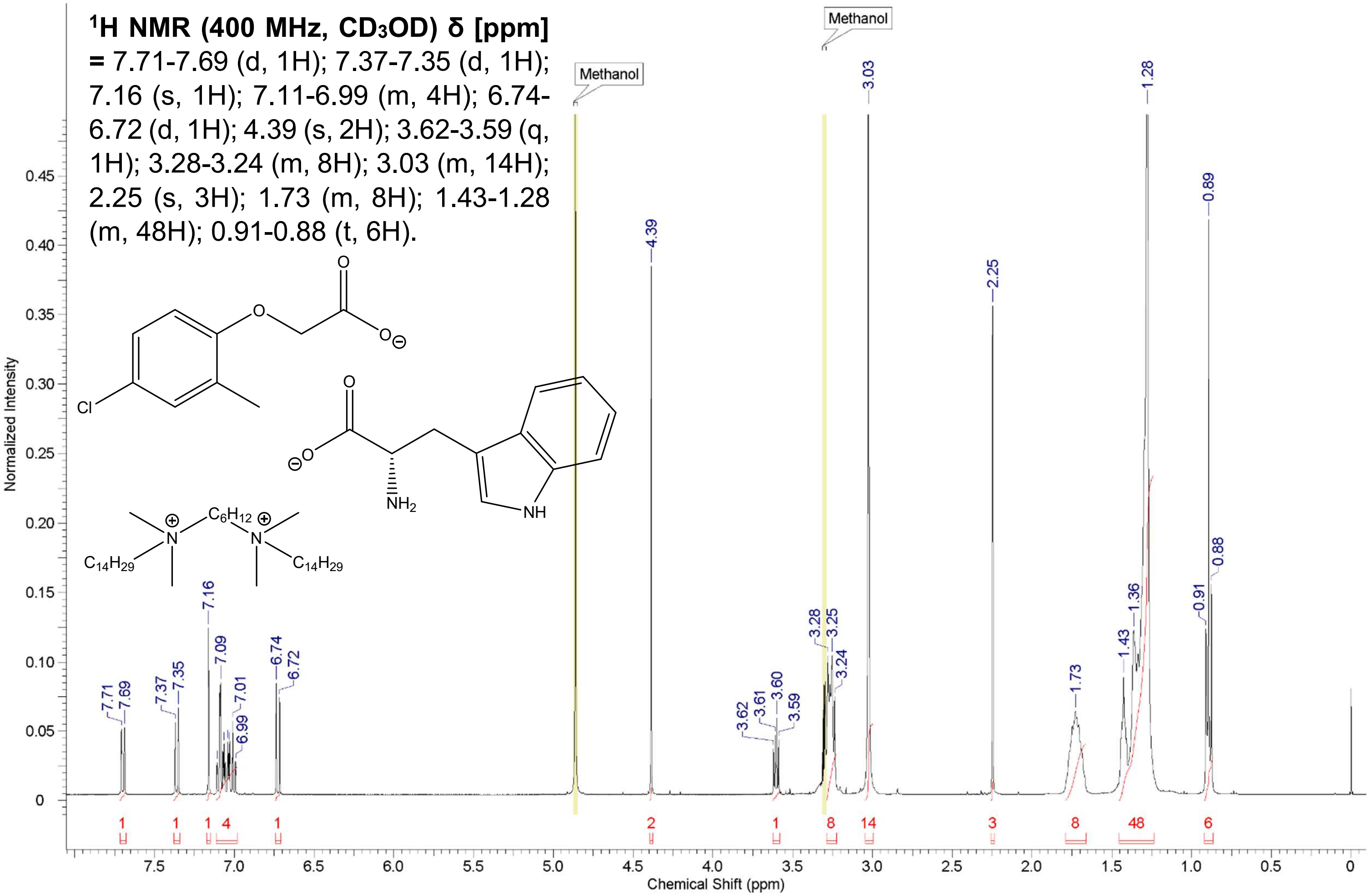

Figure S10. ¹H NMR spectrum of L-tryptophanate 4-chloro-2-methylphenoxyacetate hexamethylene-1,6-bis(tetradecyldimethylammonium) (**4**)

File Name						***Frequency (MHz)***	100.57
Nucleus	13C	***Number of Transients***	1144	***Original Points Count***	32768	***Points Count***	32768
Pulse Sequence	s2pul	***Receiver Gain***	52.00	***Solvent***	METHANOL-d4	***Spectrum Offset (Hz)***	10181.6465
Spectrum Type	STANDARD	***Sweep Width (Hz)***	22321.43	***Temperature (degree C)***	AMBIENT TEMPERATURE		

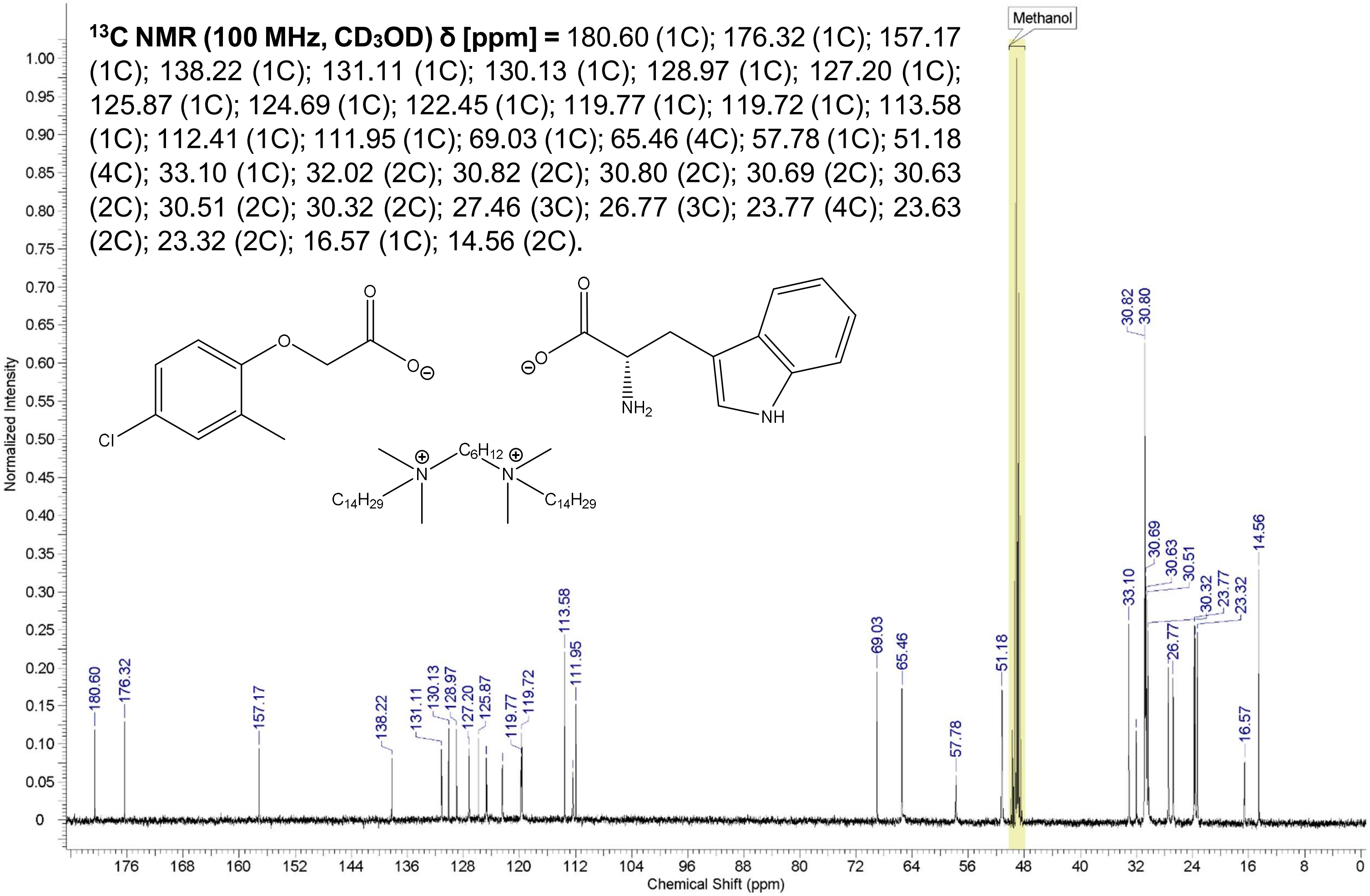

Figure S11. ^{13}C NMR spectrum of L-tryptophanate 4-chloro-2-methylphenoxyacetate hexamethylene-1,6-bis(tetradecyldimethylammonium) (**4**)

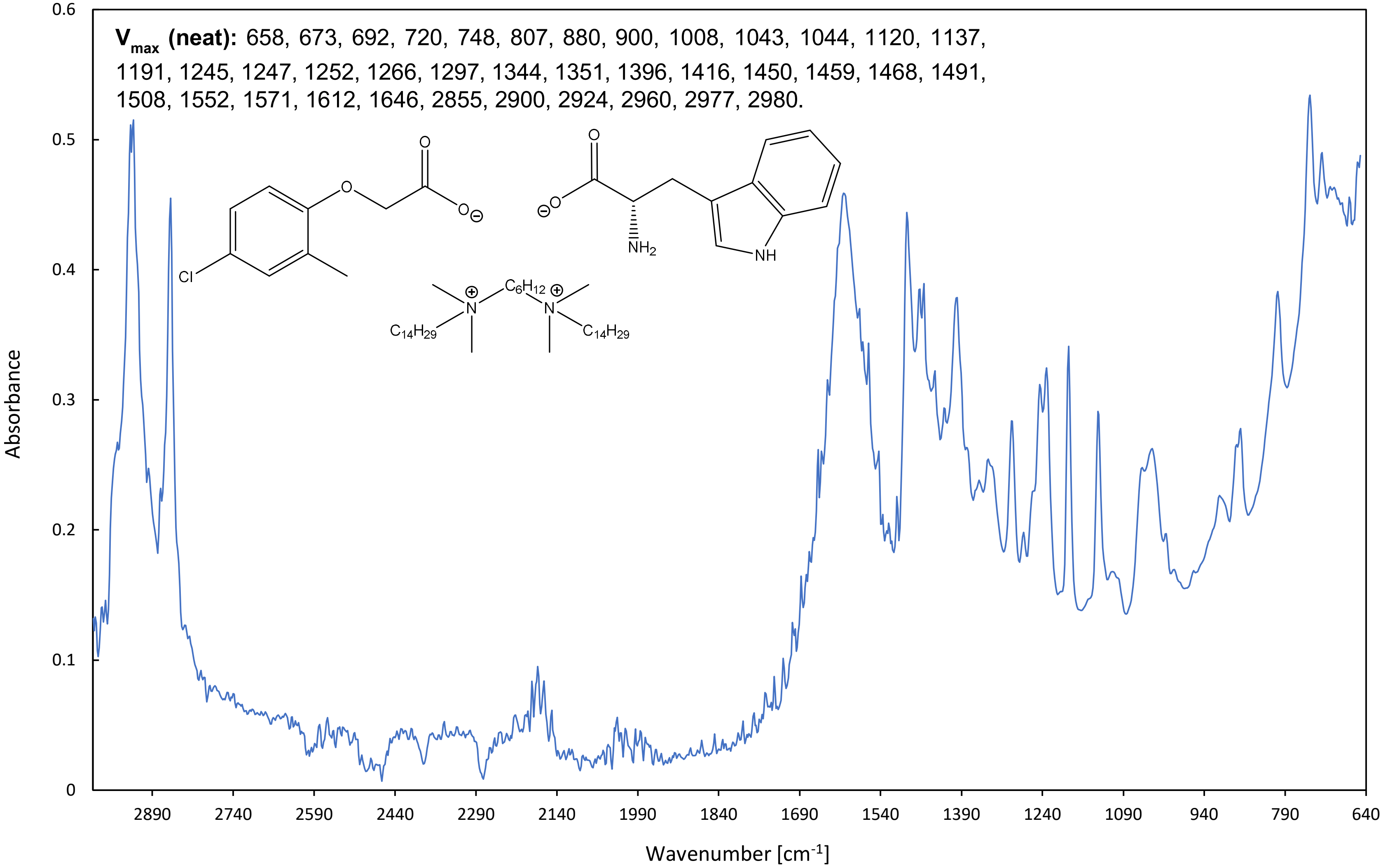

Figure S12. FT-IR spectrum of L-tryptophanate 4-chloro-2-methylphenoxyacetate hexamethylene-1,6-bis(tetradecyldimethylammonium) (**4**)

File Name						Frequency (MHz)	399.91
Nucleus	1H	Number of Transients	64	Original Points Count	30049	Points Count	32768
Pulse Sequence	s2pul	Receiver Gain	20.00	Solvent	METHANOL-d4	Spectrum Offset (Hz)	2616.3687
Spectrum Type	STANDARD	Sweep Width (Hz)	6009.62	Temperature (degree C)	25.000		

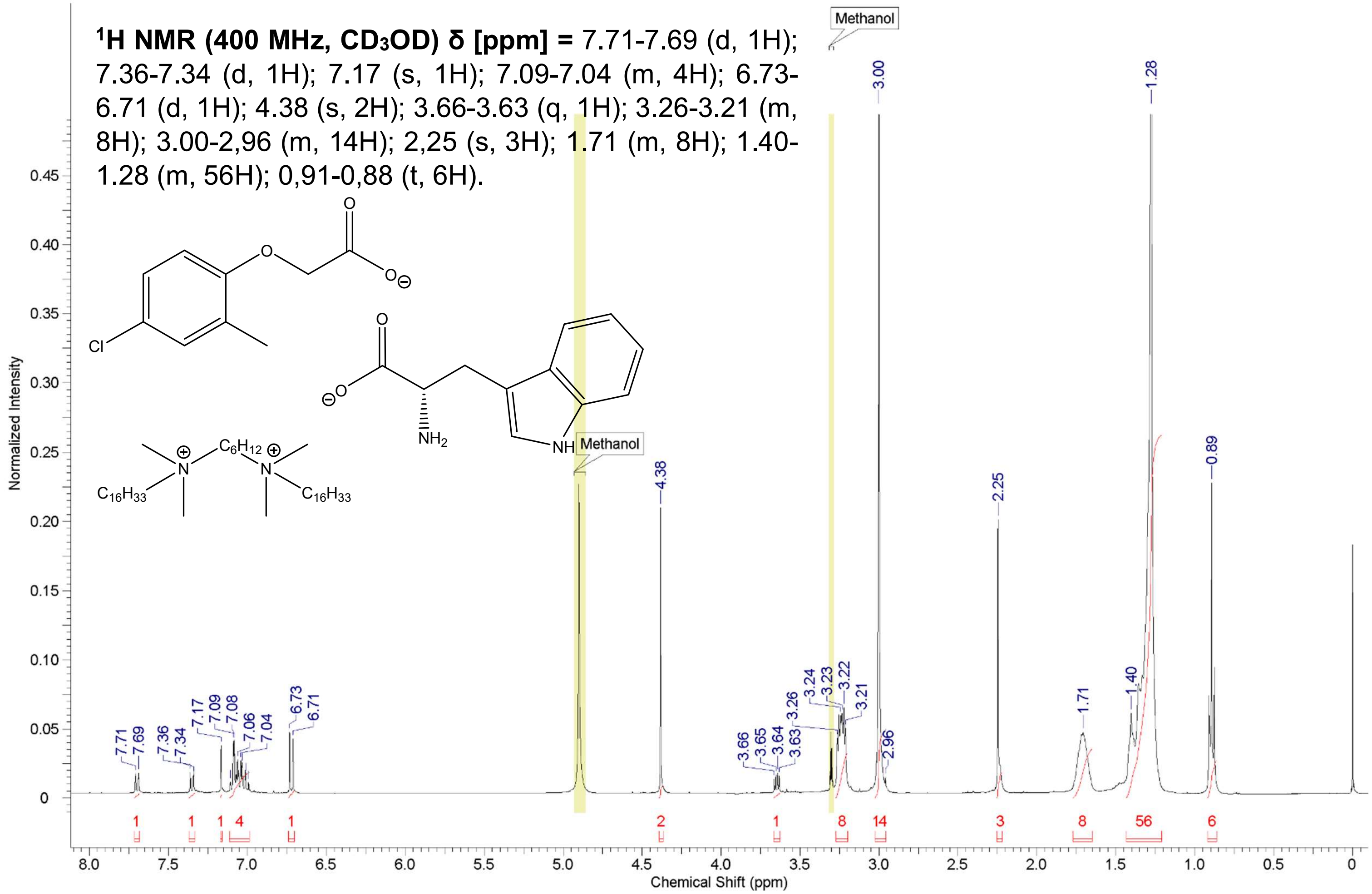

Figure S13. ^{1}H NMR spectrum of L-tryptophanate 4-chloro-2-methylphenoxyacetate hexamethylene-1,6-bis(hexadecyldimethylammonium) (**5**)

File Name						Frequency (MHz)	100.57
Nucleus	13C	Number of Transients	2800	Original Points Count	26316	Points Count	32768
Pulse Sequence	s2pul	Receiver Gain	58.00	Solvent	METHANOL-d4	Spectrum Offset (Hz)	9909.0947
Spectrum Type	STANDARD	Sweep Width (Hz)	21929.82	Temperature (degree C)	25.000		

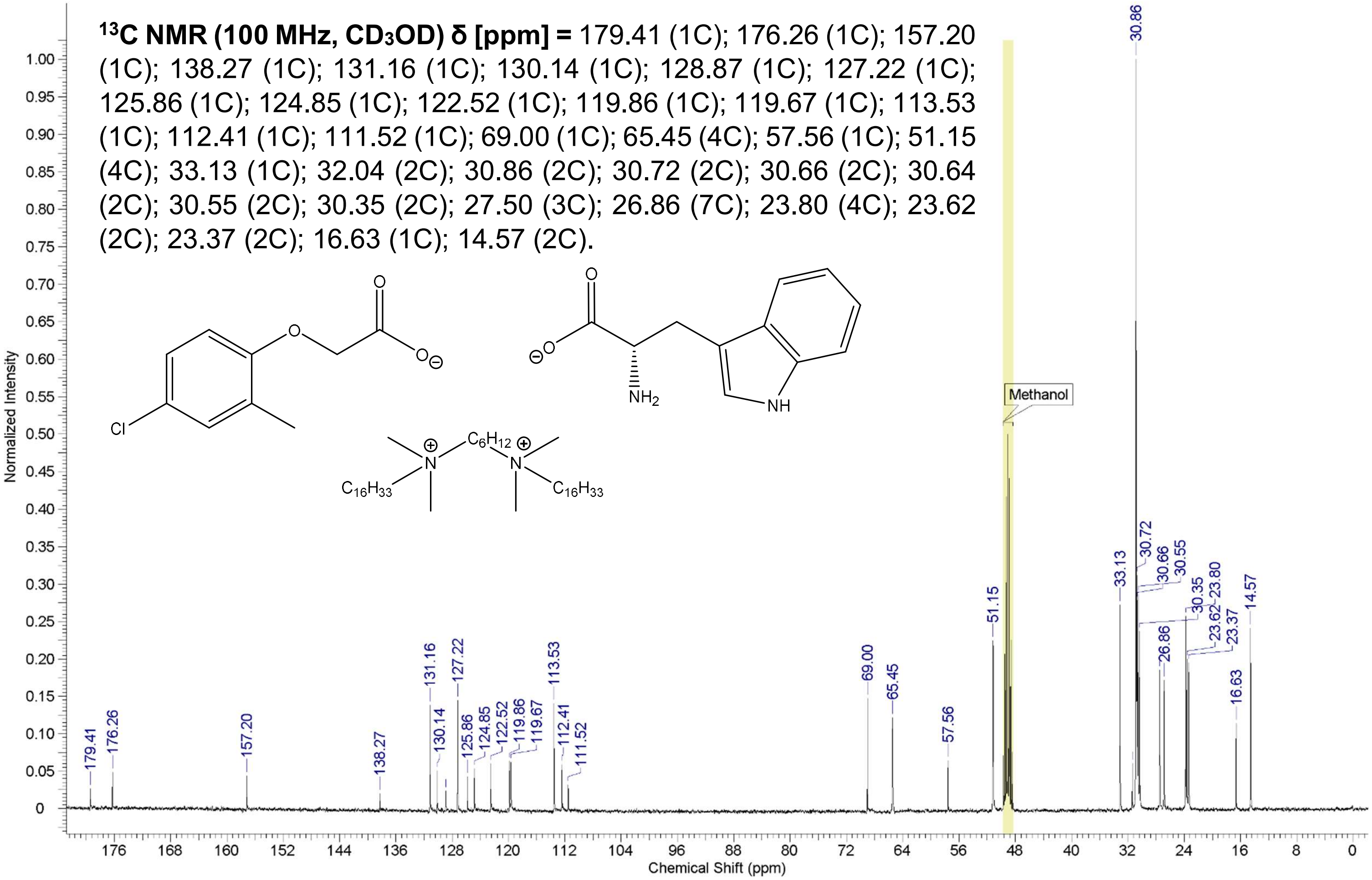

Figure S14. ^{13}C NMR spectrum of L-tryptophanate 4-chloro-2-methylphenoxyacetate hexamethylene-1,6-bis(hexadecyldimethylammonium) (**5**)

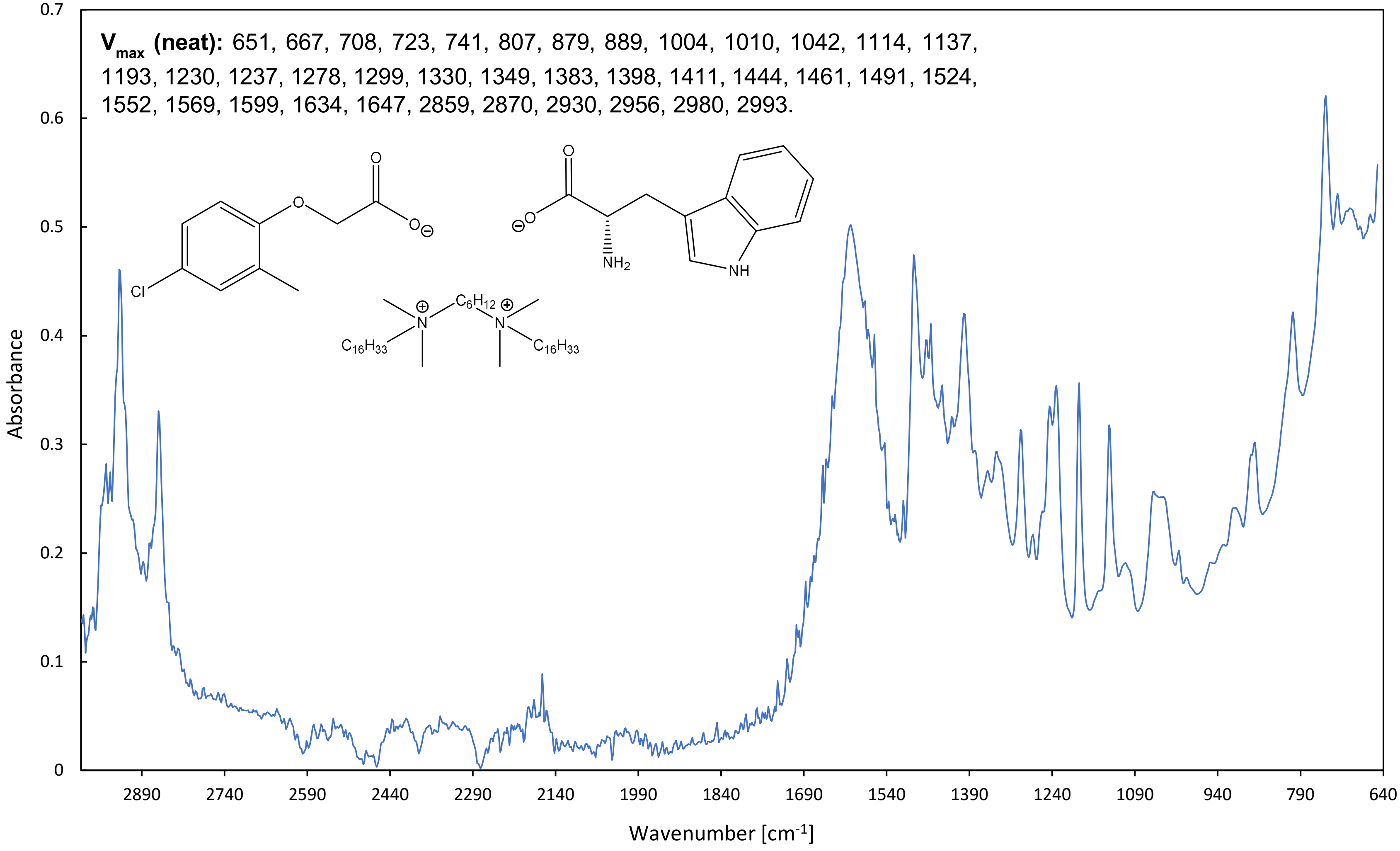

Figure S15. FT-IT NMR spectrum of L-tryptophanate 4-chloro-2-methylphenoxyacetate hexamethylene-1,6-bis(hexadecyldimethylammonium) (**5**)

File Name						Frequency (MHz)	399.91
Nucleus	1H	Number of Transients	40	Original Points Count	39773	Points Count	65536
Pulse Sequence	s2pul	Receiver Gain	22.00	Solvent	METHANOL-d4		
Spectrum Offset (Hz)	2438.2305	Spectrum Type	STANDARD	Sweep Width (Hz)	5681.82	Temperature (degree C)	25.000

^{1}H NMR (400 MHz, CD_3OD) δ [ppm] = 7.71-7.69 (d, 1H); 7.36-7.34 (d, 1H); 7,17 (s, 1H); 7.09-7.01 (m, 4H); 6.73-6.71 (d, 1H); 4.38 (s, 2H); 3.67-3.63 (q, 1H); 3.24-3.20 (m, 8H); 3.00-2.99 (m, 14H); 2,25 (s, 3H); 1.69 (m, 8H); 1.38-1.33 (m, 28H); 0.92-0.88 (t, 6H).

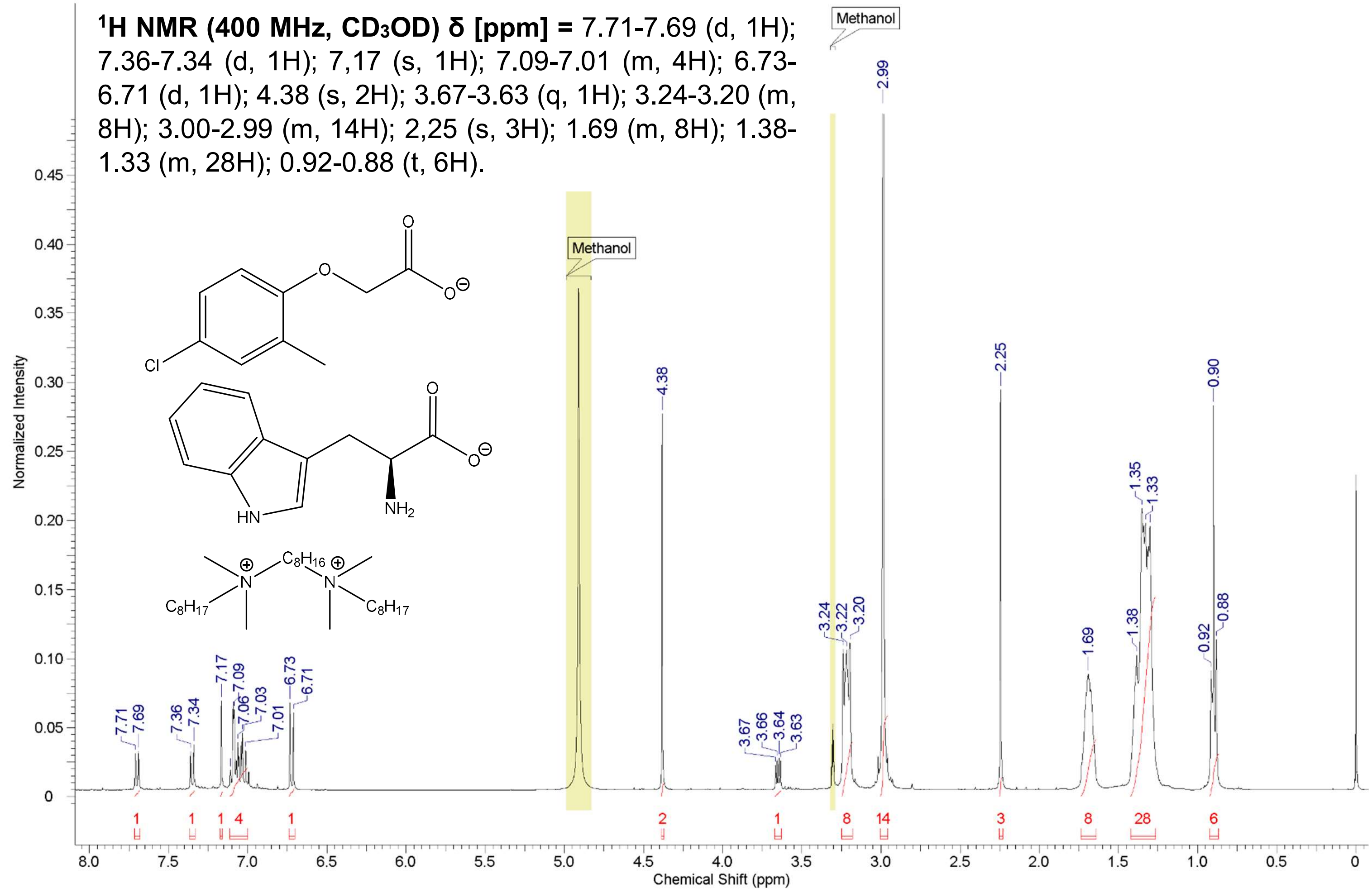

Figure S16. ^{1}H NMR spectrum of L-tryptophanate 4-chloro-2-methylphenoxyacetate octamethylene-1,8-bis(octadimethylammonium) (**6**)

File Name						Frequency (MHz)	100.57
Nucleus	13C	Number of Transients	1160	Original Points Count	26316	Points Count	32768
Pulse Sequence	s2pul	Receiver Gain	58.00	Solvent	METHANOL-d4		
Spectrum Offset (Hz)	9909.9844	Spectrum Type	STANDARD	Sweep Width (Hz)	21929.82	Temperature (degree C)	25.000

^{13}C NMR (100 MHz, CD_3OD) δ [ppm] = 179.40 (1C); 176.28 (1C); 157.22 (1C); 138.28 (1C); 131.15 (1C); 130.18 (1C); 128.97 (1C); 127.21 (1C); 125.83 (1C); 124.87 (1C); 122.52 (1C); 119.87 (1C); 119.68 (1C); 113.54 (1C); 112.41 (1C); 111.52 (1C); 69.00 (1C); 65.35 (4C); 57.56 (1C); 51.14 (4C); 32.92 (1C); 32.08 (2C); 30.28 (4C); 30.09 (2C); 27.44 (4C); 25.34 (4C); 23.56 (2C); 16.60 (1C); 14.51 (2C).

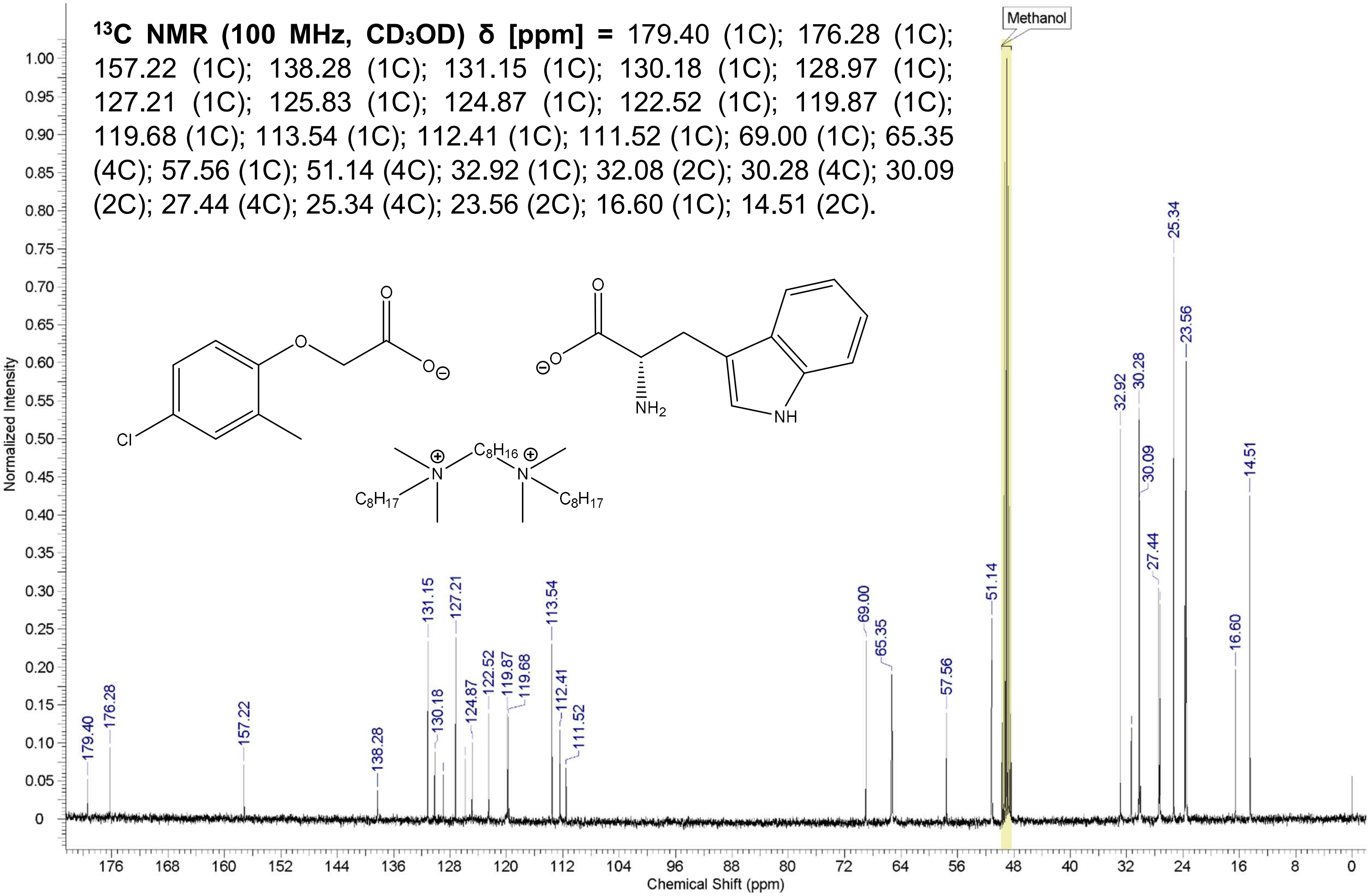

Figure S17. ^{13}C NMR spectrum of L-tryptophanate 4-chloro-2-methylphenoxyacetate octamethylene-1,8-bis(octadimethylammonium) (**6**)

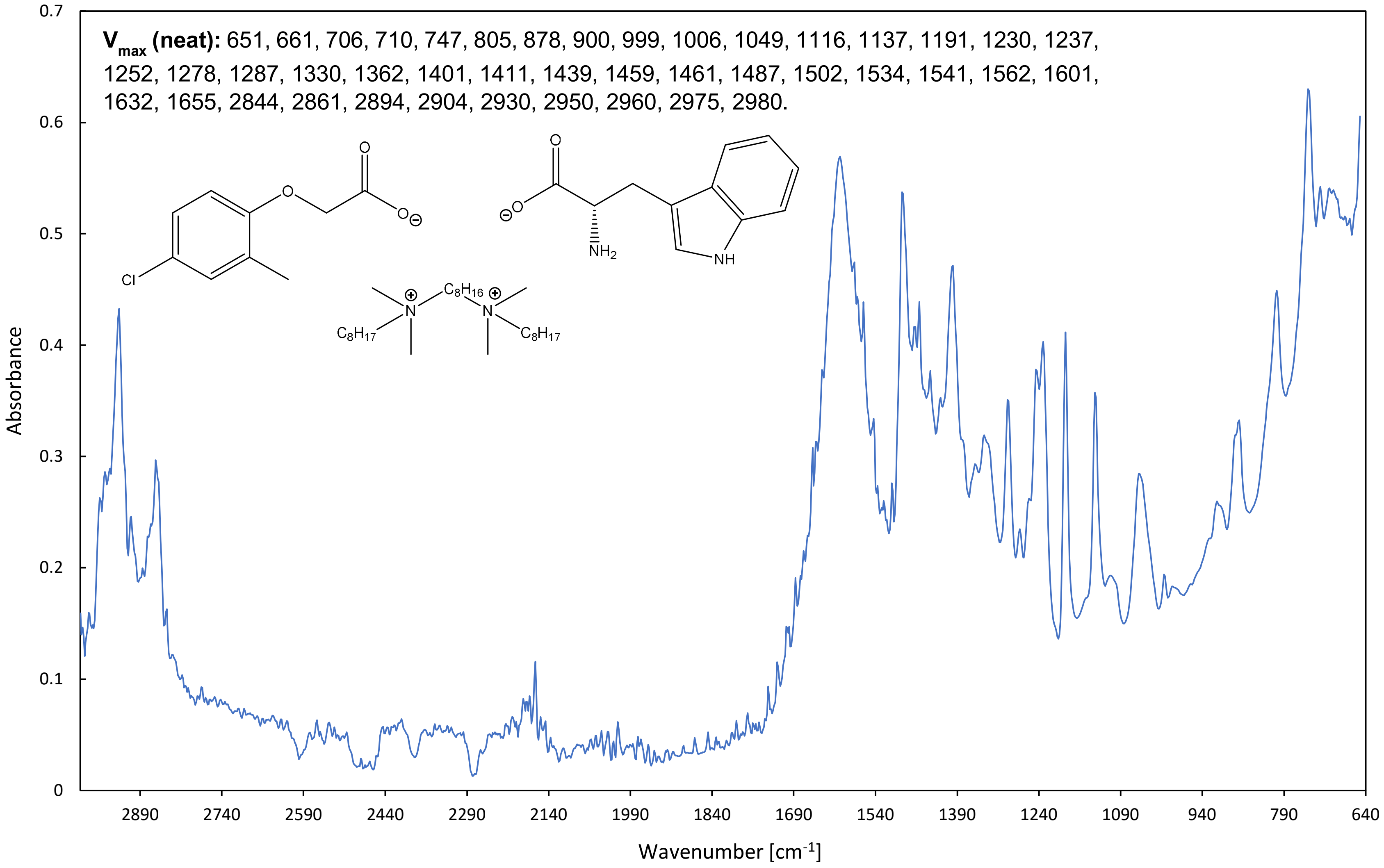

Figure S18. FT-IR spectrum of L-tryptophanate 4-chloro-2-methylphenoxyacetate octamethylene-1,8-bis(octadimethylammonium) (**6**)

File Name						Frequency (MHz)	399.91
Nucleus	1H	Number of Transients	64	Original Points Count	31566	Points Count	32768
Pulse Sequence	s2pul	Receiver Gain	20.00	Solvent	METHANOL-d4	Spectrum Offset (Hz)	2759.6580
Spectrum Type	STANDARD	Sweep Width (Hz)	6313.13	Temperature (degree C)	25.000		

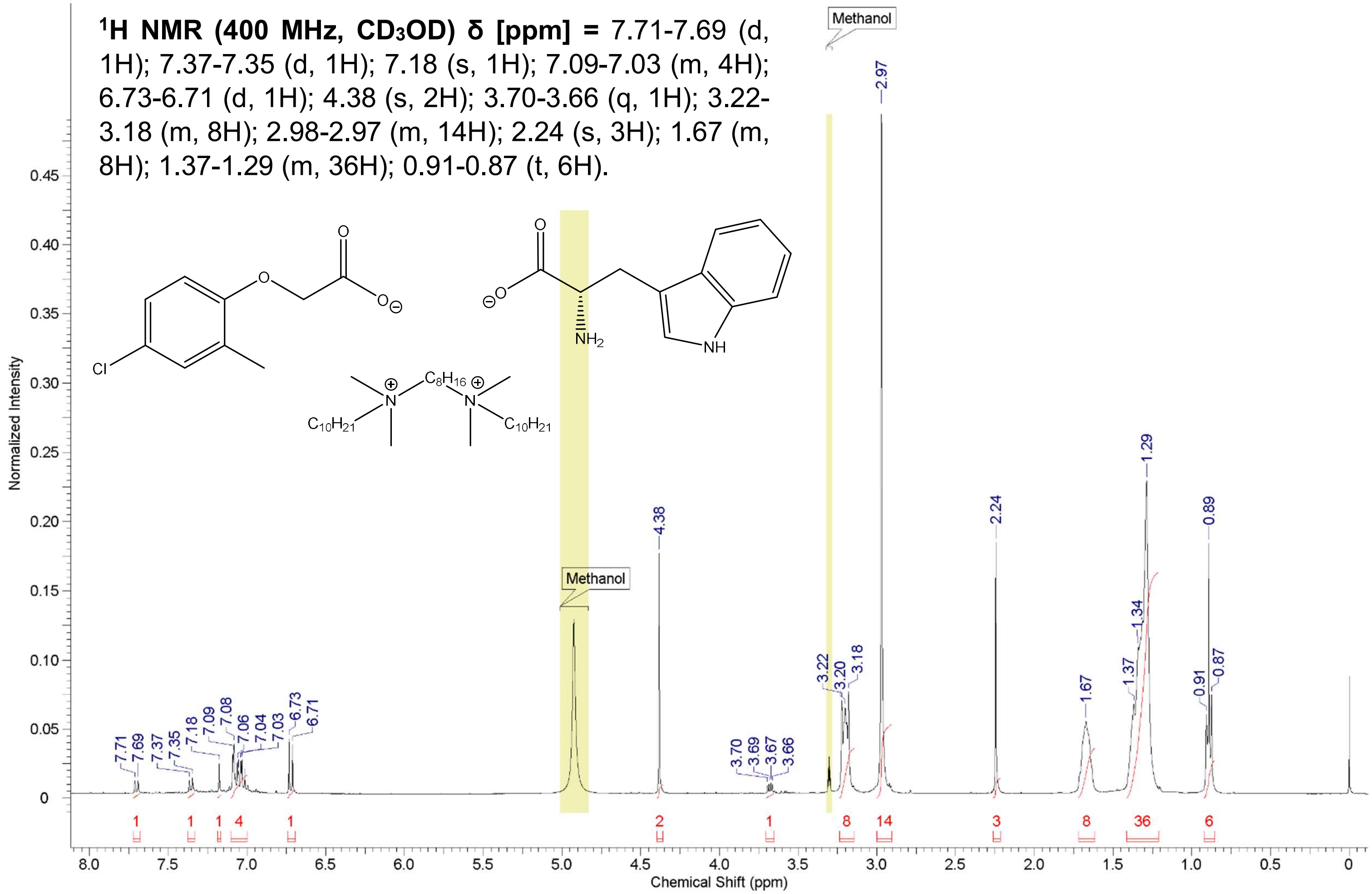

Figure S19. ^{1}H NMR spectrum of L-tryptophanate 4-chloro-2-methylphenoxyacetate octamethylene-1,8-bis(decyldimethylammonium) (**7**)

File Name						***Frequency (MHz)***	100.57
Nucleus	13C	***Number of Transients***	1552	***Original Points Count***	26316	***Points Count***	32768
Pulse Sequence	s2pul	***Receiver Gain***	58.00	***Solvent***	METHANOL-d4	***Spectrum Offset (Hz)***	9908.0918
Spectrum Type	STANDARD	***Sweep Width (Hz)***	21929.82	***Temperature (degree C)***	25.000		

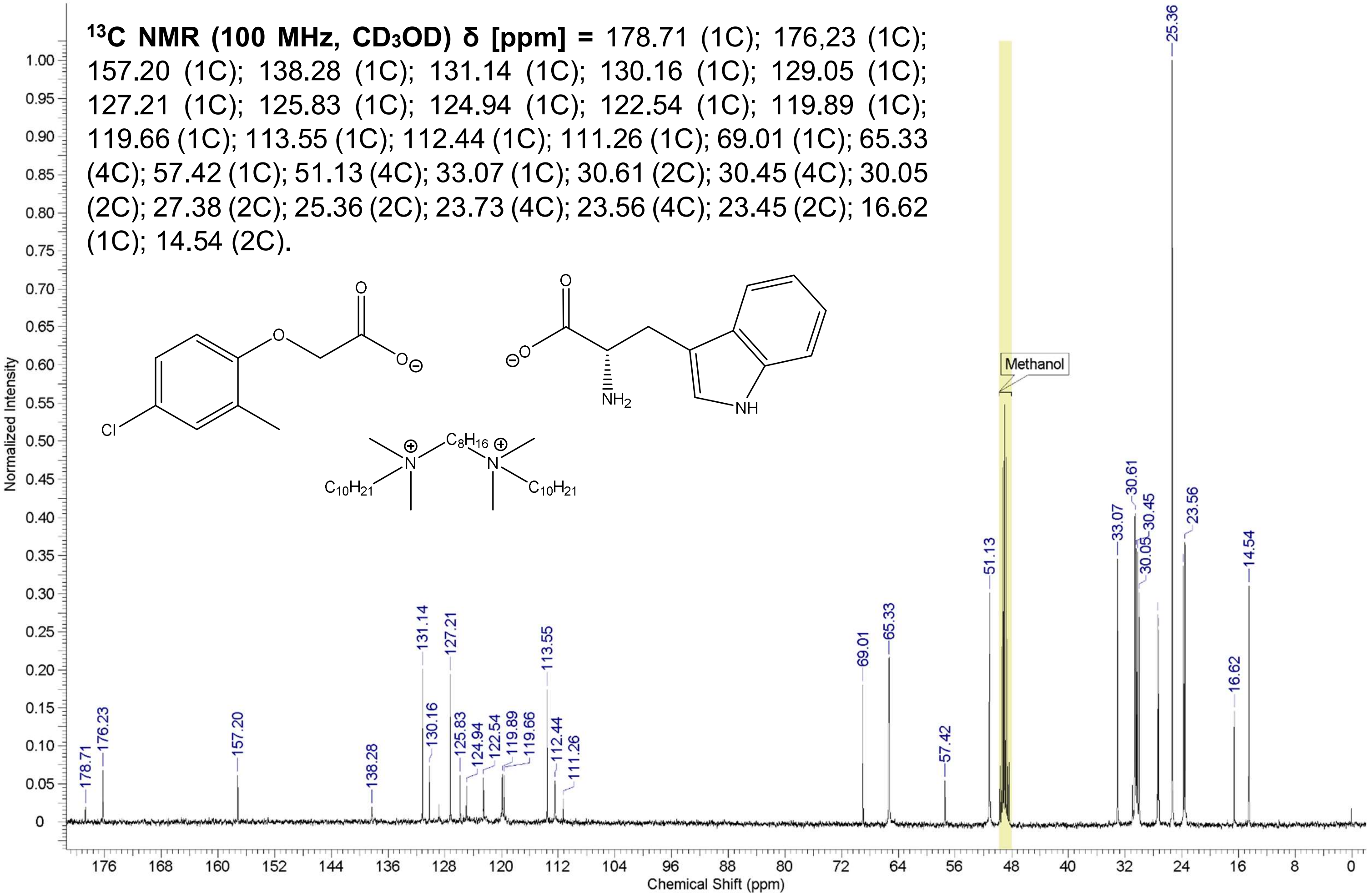

Figure S20. ^{13}C NMR spectrum of L-tryptophanate 4-chloro-2-methylphenoxyacetate octamethylene-1,8-bis(decyldimethylammonium) (**7**)

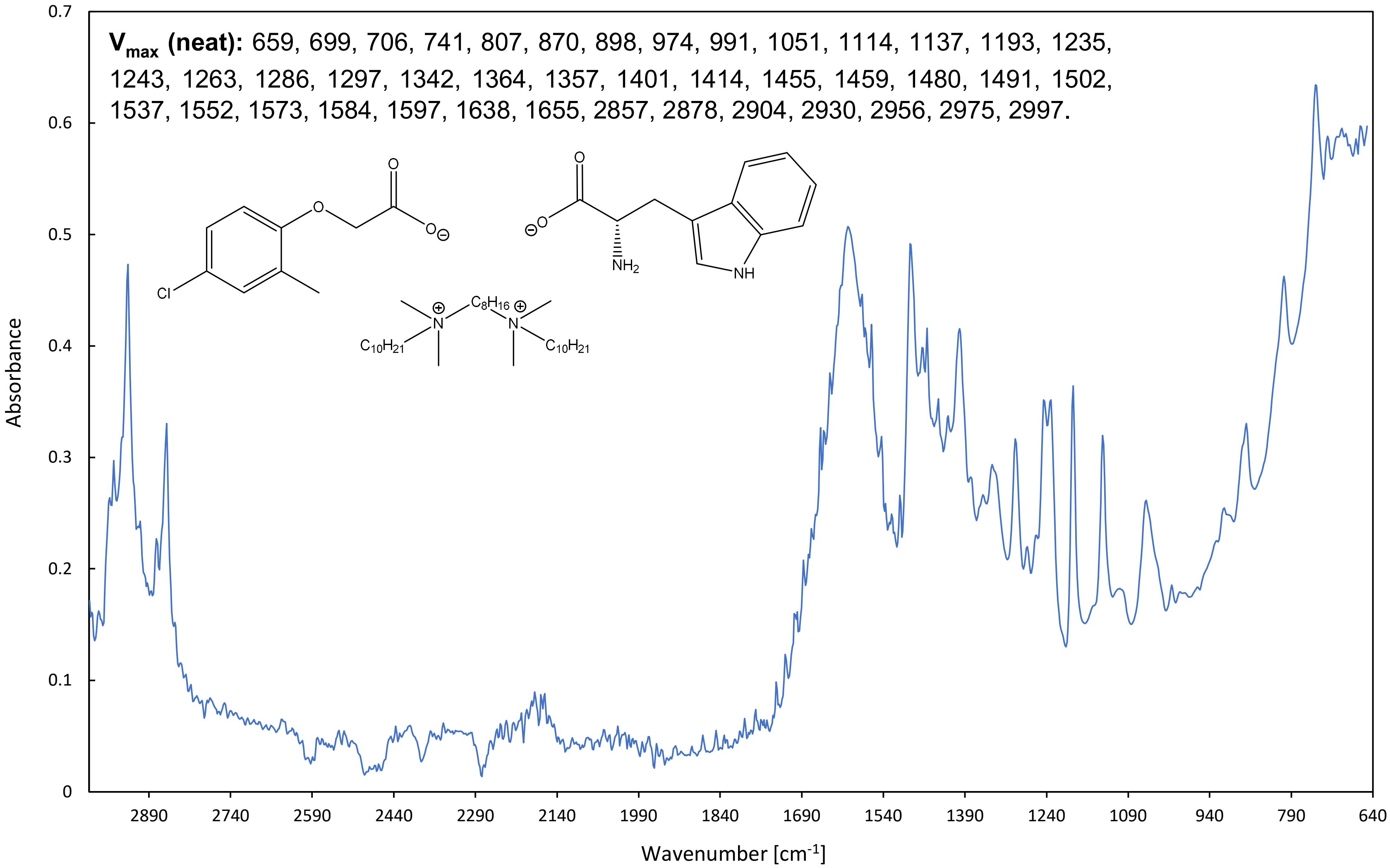

Figure S21. FT-IR spe ctrum of L-tryptophanate 4-chloro-2-methylphenoxyacetate octamethylene-1,8-bis(decyldimethylammonium) (**7**)

**File Name**						**Frequency (MHz)**	399.91
**Nucleus**	1H	**Number of Transients**	1	**Original Points Count**	29206	**Points Count**	32768
**Pulse Sequence**	s2pul	**Receiver Gain**	20.00	**Solvent**	METHANOL-d4		
**Spectrum Offset (Hz)**	2544.1714	**Spectrum Type**	STANDARD	**Sweep Width (Hz)**	5841.12	**Temperature (degree C)**	25.000

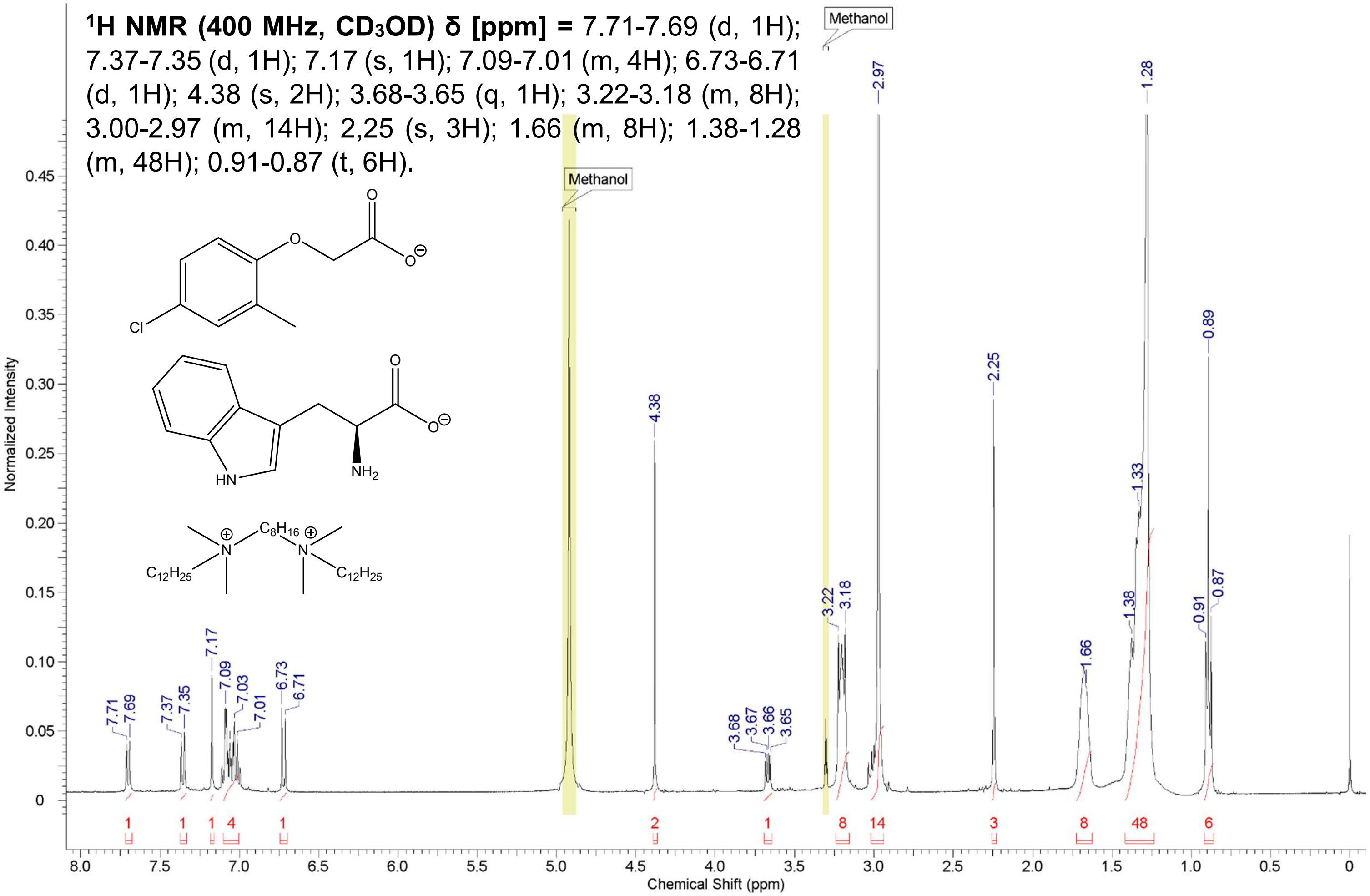

Figure S22. ^{1}H NMR spectrum of L-tryptophanate 4-chloro-2-methylphenoxyacetate octamethylene-1,8-bis(dodecyldimethylammonium) (**8**)

File Name						***Frequency (MHz)***	100.57
Nucleus	13C	***Number of Transients***	804	***Original Points Count***	26316	***Points Count***	32768
Pulse Sequence	s2pul	***Receiver Gain***	58.00	***Solvent***	METHANOL-d4	***Spectrum Offset (Hz)***	9907.7568
Spectrum Type	STANDARD	***Sweep Width (Hz)***	21929.82	***Temperature (degree C)***	25.000		

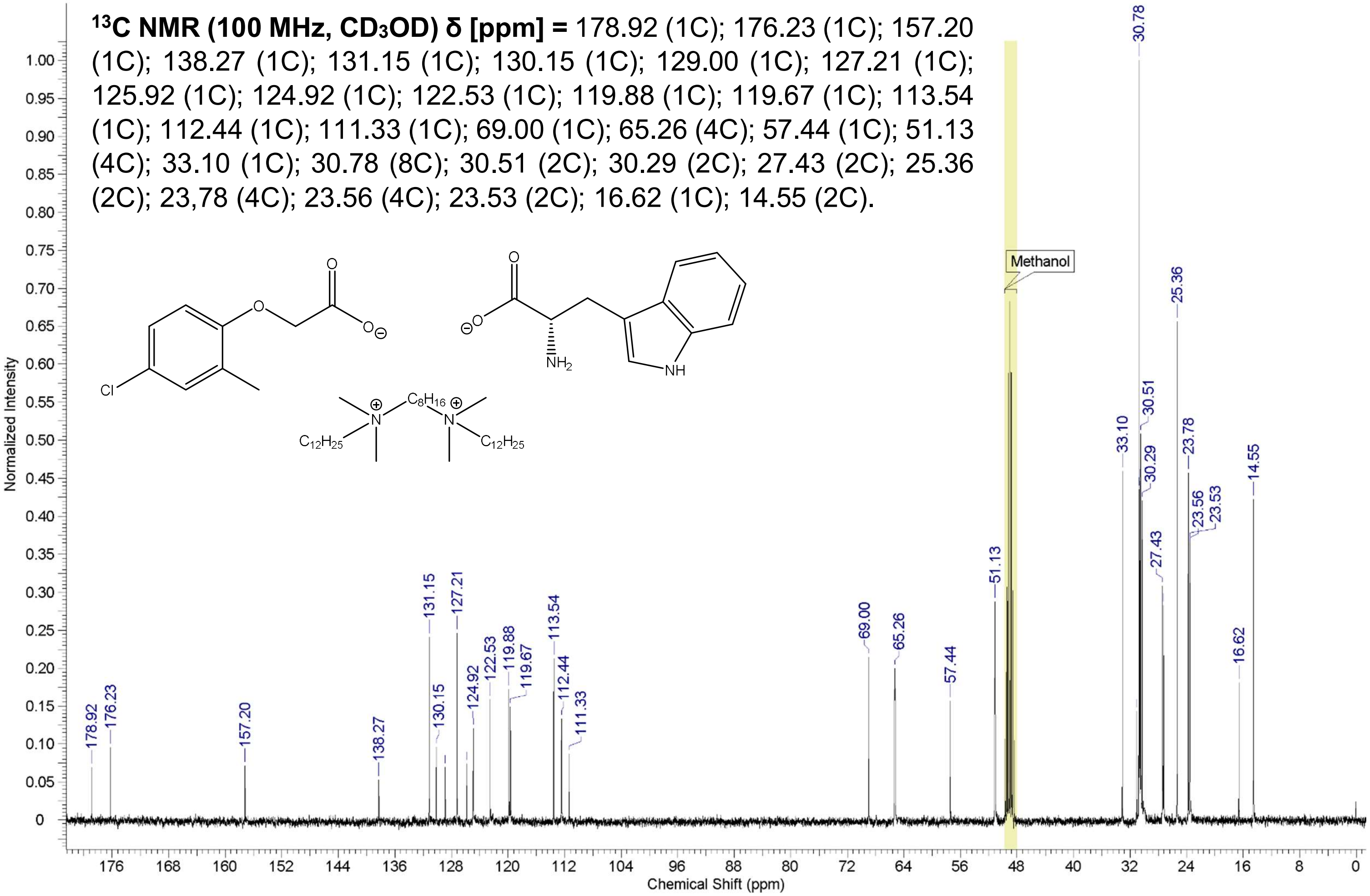

Figure S23. ^{13}C NMR spectrum of L-tryptophanate 4-chloro-2-methylphenoxyacetate octamethylene-1,8-bis(dodecyldimethylammonium) (**8**)

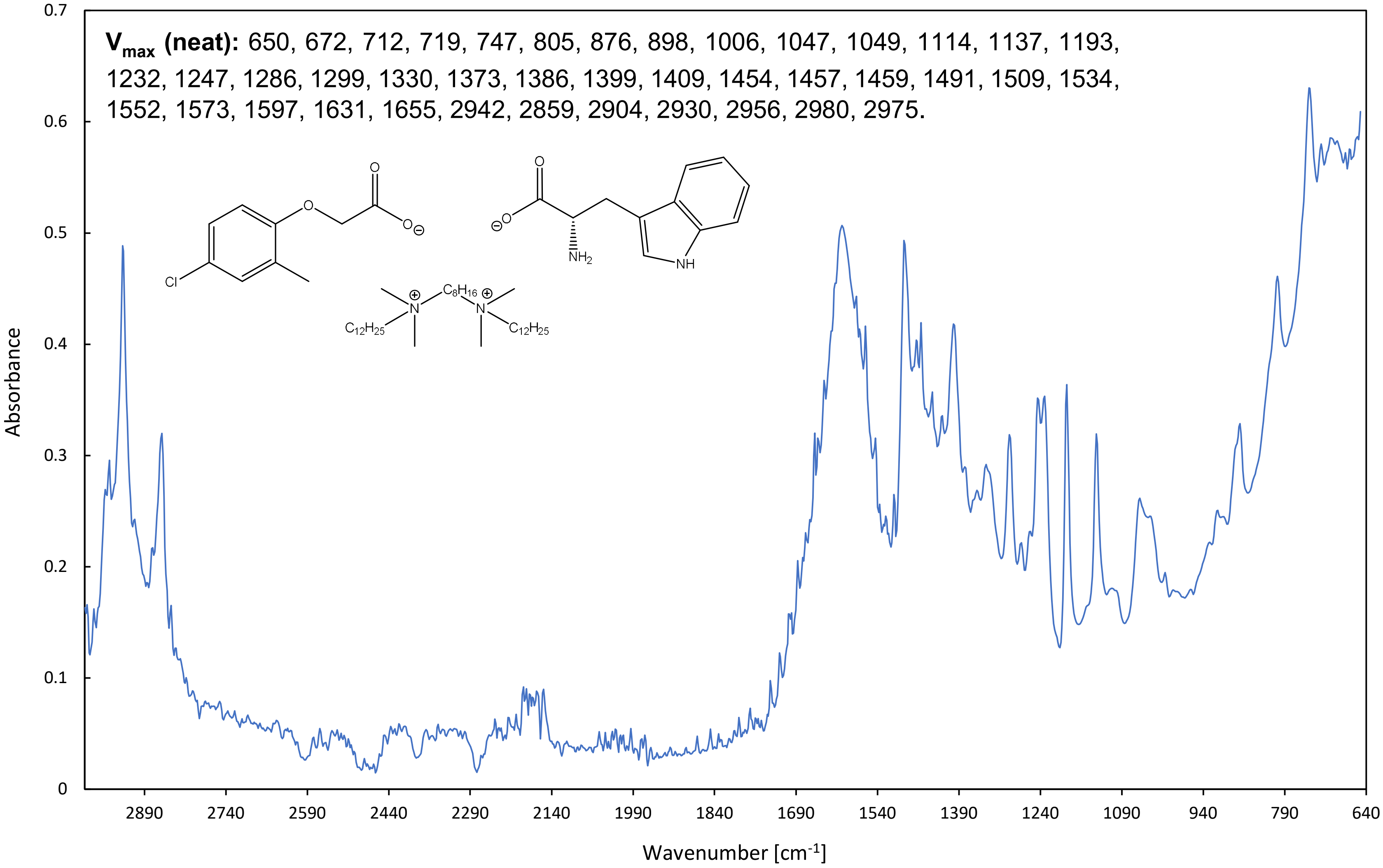

Figure S24. FT-IR spectrum of L-tryptophanate 4-chloro-2-methylphenoxyacetate octamethylene-1,8-bis(dodecyldimethylammonium) (**8**)

File Name						Frequency (MHz)	399.91
Nucleus	1H	Number of Transients	40	Original Points Count	50448	Points Count	65536
Pulse Sequence	s2pul	Receiver Gain	20.00	Solvent	METHANOL-d4	Spectrum Offset (Hz)	2413.9507
Spectrum Type	STANDARD	Sweep Width (Hz)	5605.38	Temperature (degree C)	AMBIENT TEMPERATURE		

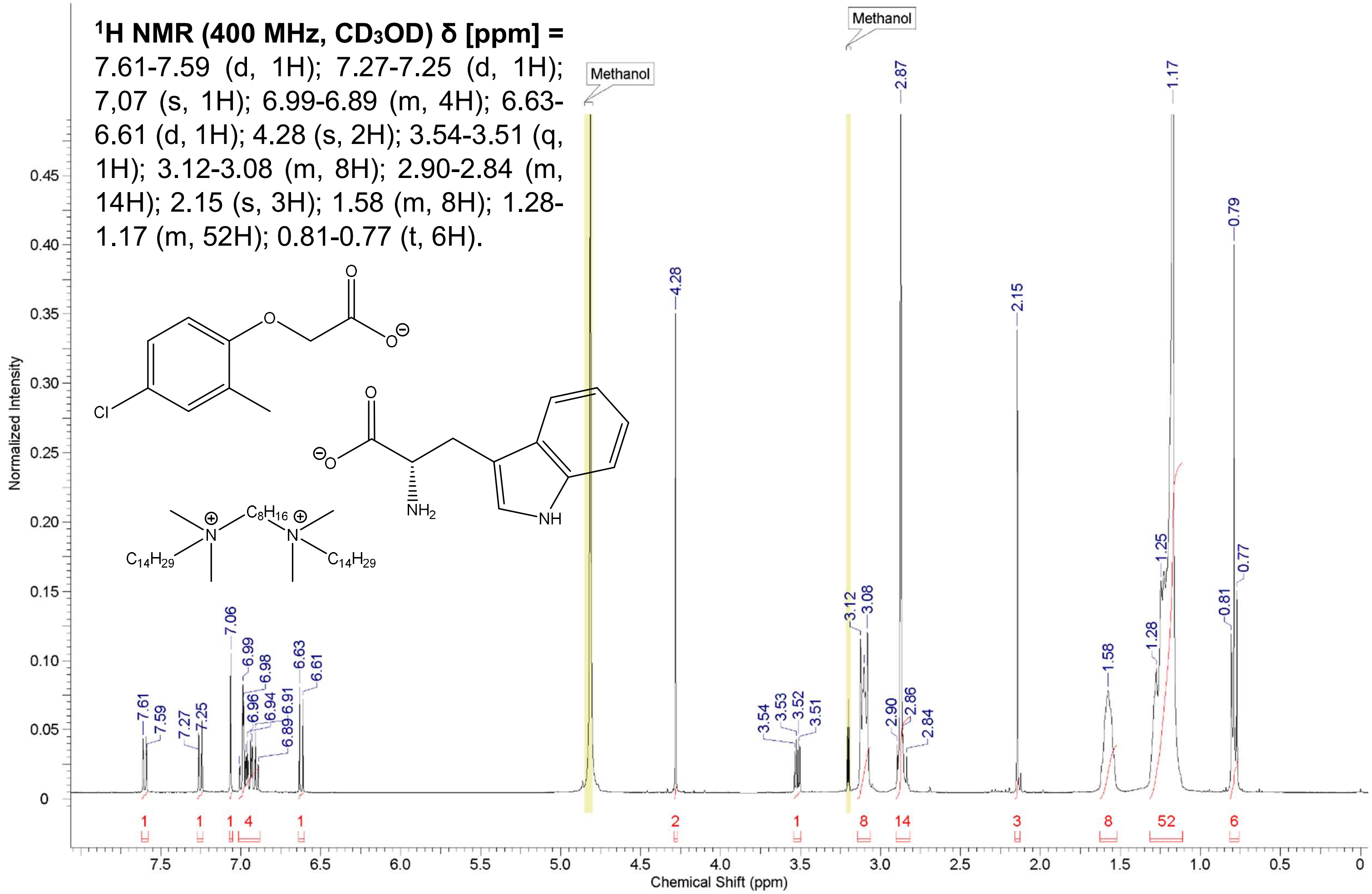

Figure S25. ¹H NMR spectrum of L-tryptophanate 4-chloro-2-methylphenoxyacetate octamethylene-1,8-bis(tetradecyldimethylammonium) (**9**)

File Name						***Frequency (MHz)***	100.57
Nucleus	13C	***Number of Transients***	536	***Original Points Count***	32328	***Points Count***	32768
Pulse Sequence	s2pul	***Receiver Gain***	54.00	***Solvent***	METHANOL-d4	***Spectrum Offset (Hz)***	9711.2461
Spectrum Type	STANDARD	***Sweep Width (Hz)***	21551.72	***Temperature (degree C)***	AMBIENT TEMPERATURE		

^{13}C NMR (100 MHz, CD_3OD) δ [ppm] = 179.97 (1C); 176.21 (1C); 157.19 (1C); 138,24 (1C); 131.14 (1C); 130.14 (1C); 128.95 (1C); 127.22 (1C); 125.82 (1C); 124.59 (1C); 122.47 (1C); 119.83 (1C); 119.71 (1C); 113.52 (1C); 112.41 (1C); 111.74 (1C); 68.98 (1C); 65.25 (4C); 57.65 (1C); 51.12 (4C); 33.11 (1C); 30.82 (12C); 30.69 (2C); 30.61 (2C); 30.29 (2C); 27.27 (4C); 23.79 (4C); 23.55 (4C); 23.53 (2C); 16.63 (1C); 14.57 (2C).

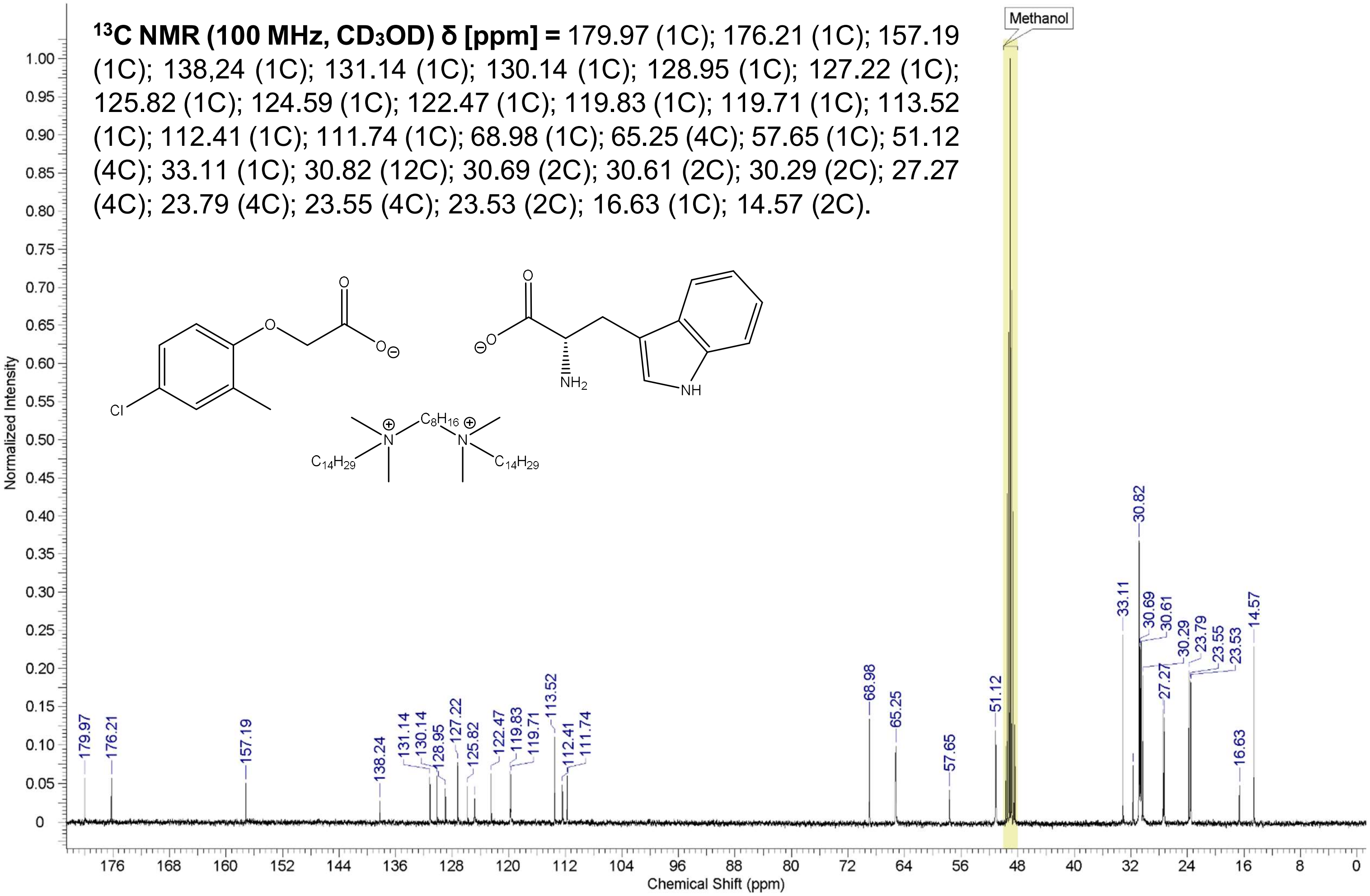

Figure S26. ^{13}C NMR spectrum of L-tryptophanate 4-chloro-2-methylphenoxyacetate octamethylene-1,8-bis(tetradecyldimethylammonium) (**9**)

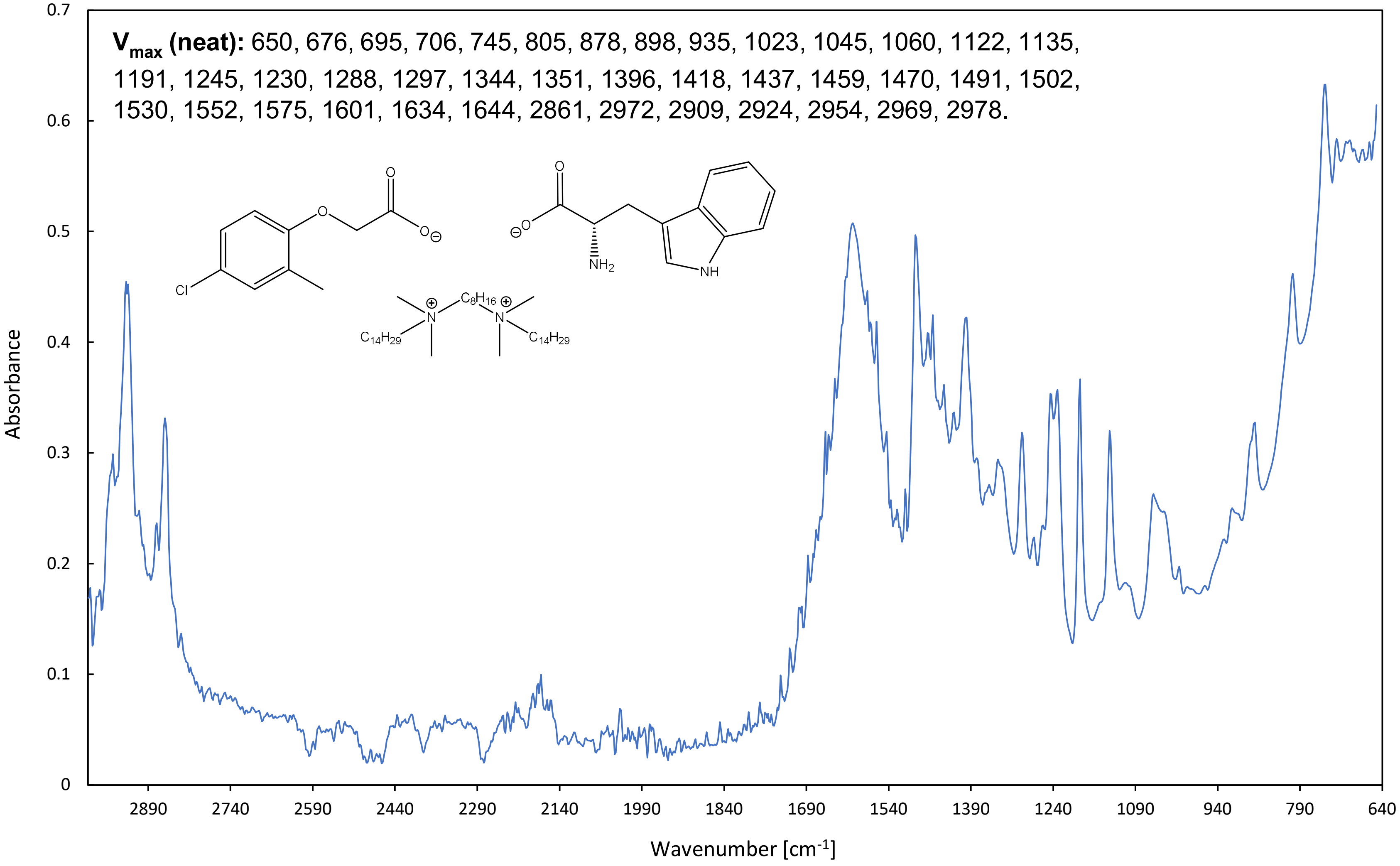

Figure S27. FT-IR spectrum of L-tryptophanate 4-chloro-2-methylphenoxyacetate octamethylene-1,8-bis(tetradecyldimethylammonium) (**9**)

File Name						Frequency (MHz)	399.91
Nucleus	1H	Number of Transients	40	Original Points Count	44843	Points Count	65536
Pulse Sequence	s2pul	Receiver Gain	20.00	Solvent	METHANOL-d4	Spectrum Offset (Hz)	2414.1440
Spectrum Type	STANDARD	Sweep Width (Hz)	5605.38	Temperature (degree C)	AMBIENT TEMPERATURE		

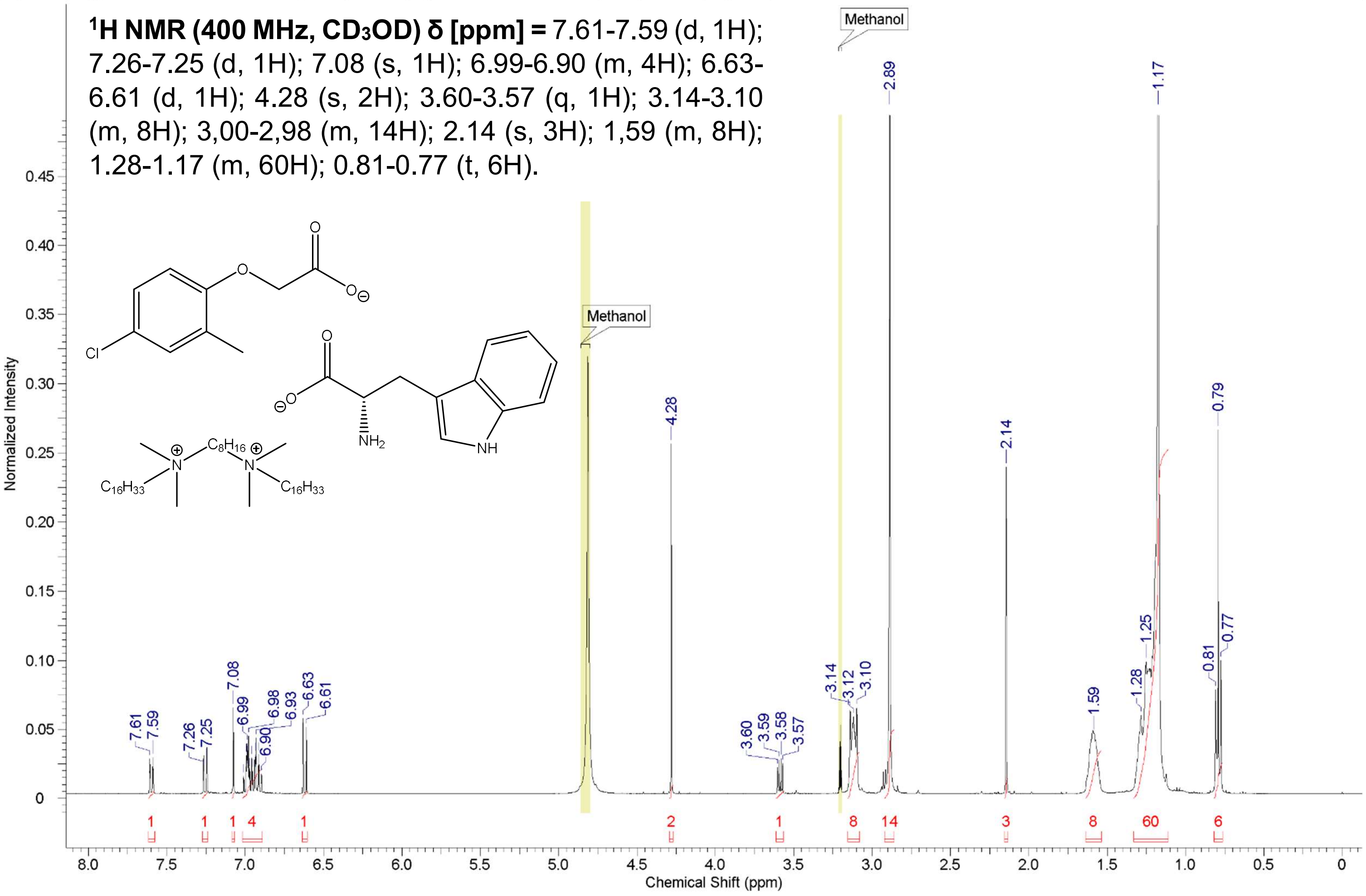

Figure S28. ^{1}H NMR spectrum of L-tryptophanate 4-chloro-2-methylphenoxyacetate octamethylene-1,8-bis(hexadecyldimethylammonium) (**10**)

File Name						Frequency (MHz)	100.57
Nucleus	13C	Number of Transients	660	Original Points Count	32328	Points Count	32768
Pulse Sequence	s2pul	Receiver Gain	54.00	Solvent	METHANOL-d4	Spectrum Offset (Hz)	9711.9023
Spectrum Type	STANDARD	Sweep Width (Hz)	21551.72	Temperature (degree C)	AMBIENT TEMPERATURE		

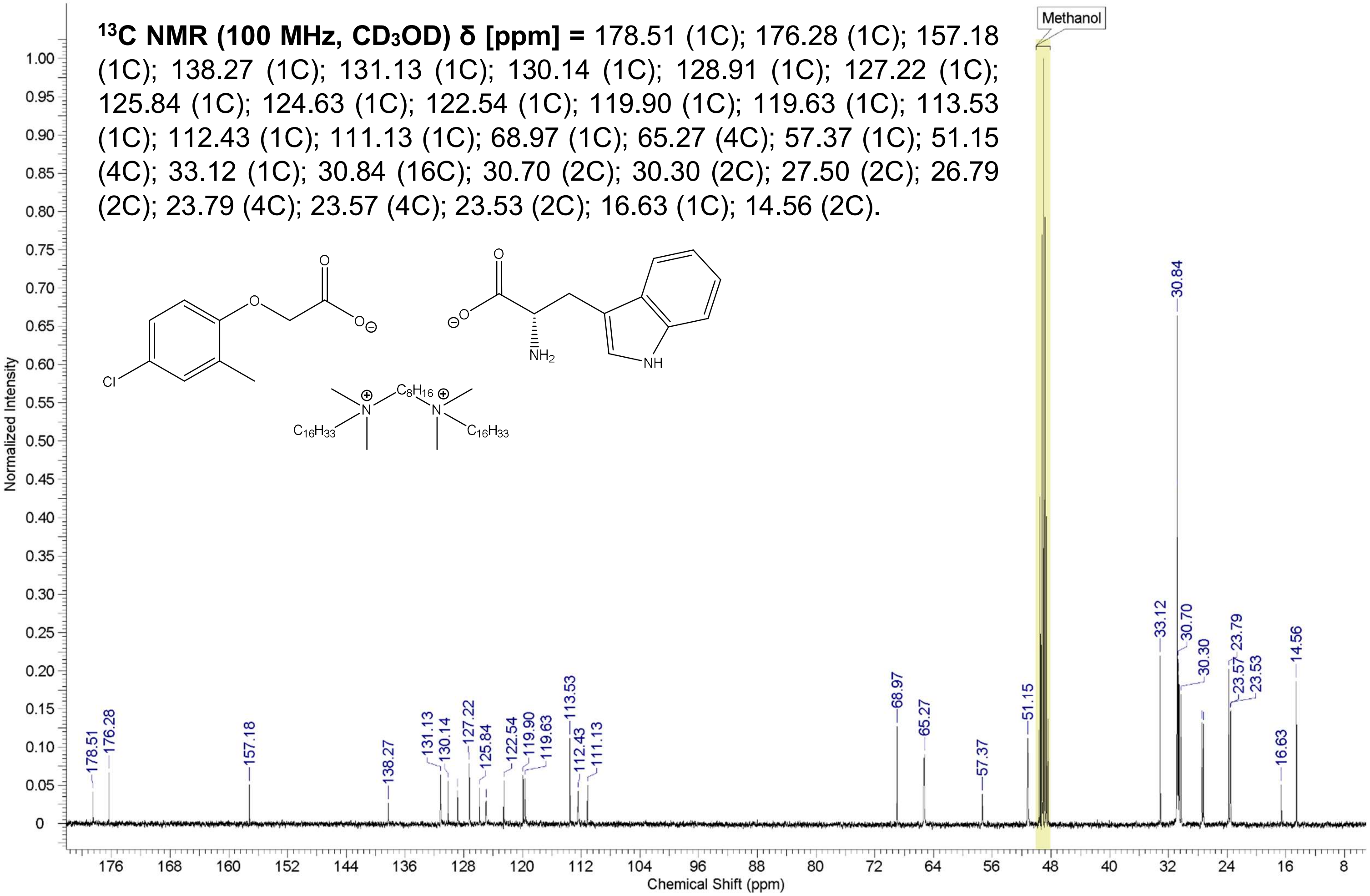

Figure S29. ^{13}C NMR spectrum of L-tryptophanate 4-chloro-2-methylphenoxyacetate octamethylene-1,8-bis(hexadecyldimethylammonium) (**10**)

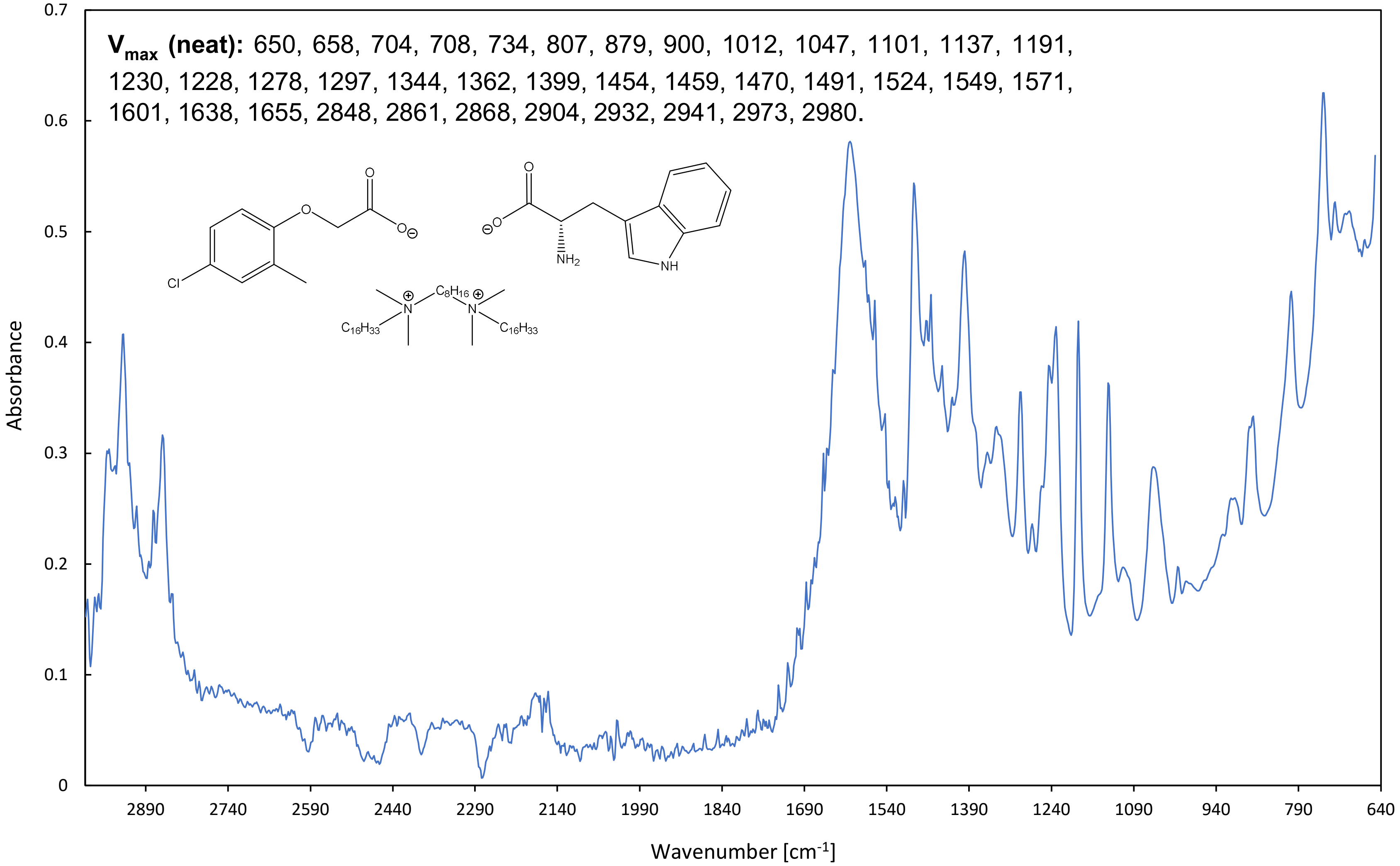

Figure S30. FT-IR spectrum of L-tryptophanate 4-chloro-2-methylphenoxyacetate octamethylene-1,8-bis(hexadecyldimethylammonium) (**10**)

File Name						Frequency (MHz)	401.13
Nucleus	1H	Number of Transients	64	Original Points Count	34423	Points Count	65536
Pulse Sequence	s2pul	Receiver Gain	10.00	Solvent	METHANOL-d4	Spectrum Offset (Hz)	2146.3857
Spectrum Type	STANDARD	Sweep Width (Hz)	5737.23	Temperature (degree C)	AMBIENT TEMPERATURE		

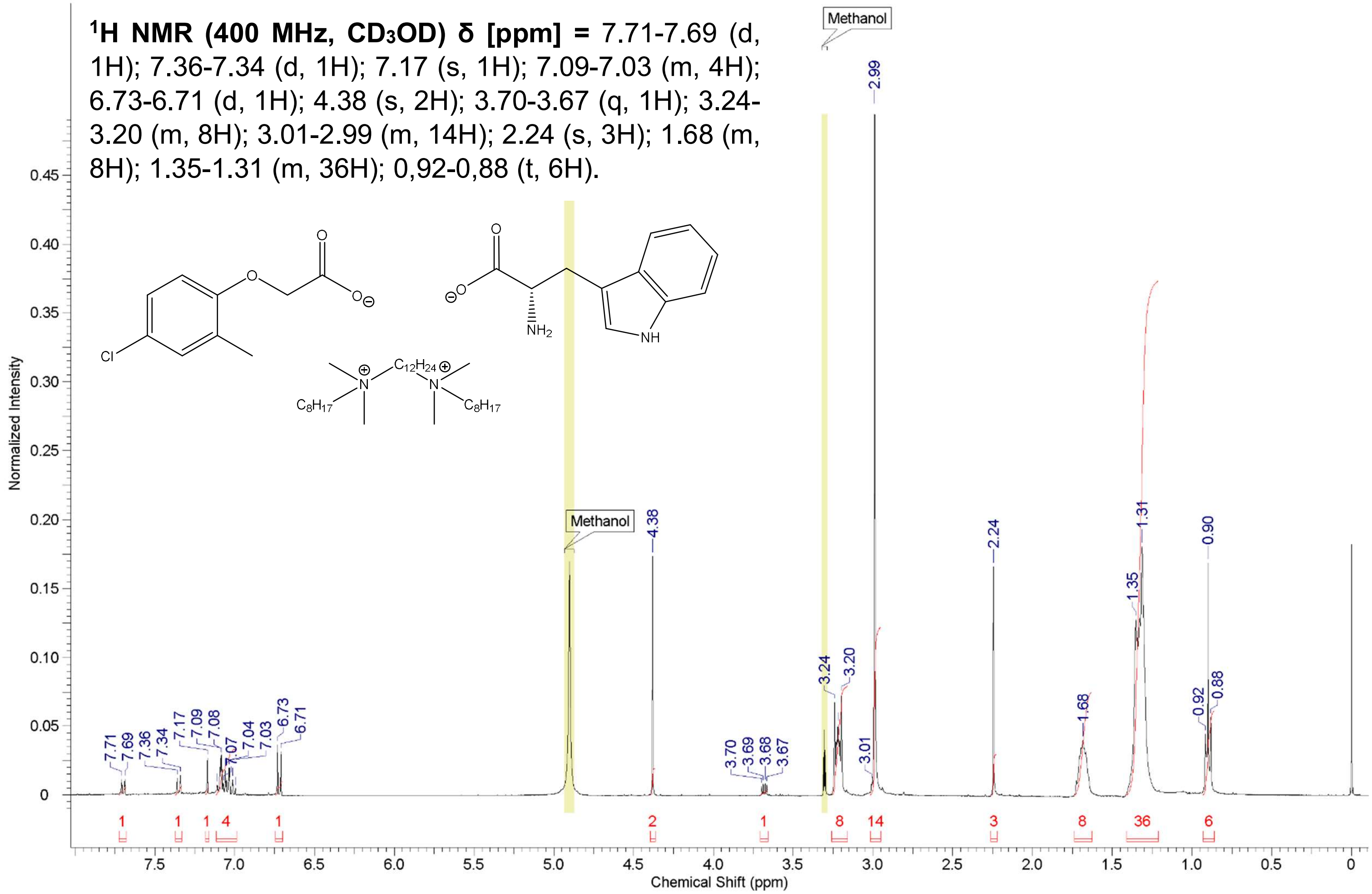

Figure S31. ^{1}H NMR spectrum of L-tryptophanate 4-chloro-2-methylphenoxyacetate dodecylmethylene-1,12-bis(octadimethylammonium) (**11**)

File Name						Frequency (MHz)	100.87
Nucleus	13C	Number of Transients	4688	Original Points Count	25199	Points Count	32768
Pulse Sequence	s2pul	Receiver Gain	24.00	Solvent	METHANOL-d4	Spectrum Offset (Hz)	10967.4814
Spectrum Type	STANDARD	Sweep Width (Hz)	26525.20	Temperature (degree C)	AMBIENT TEMPERATURE		

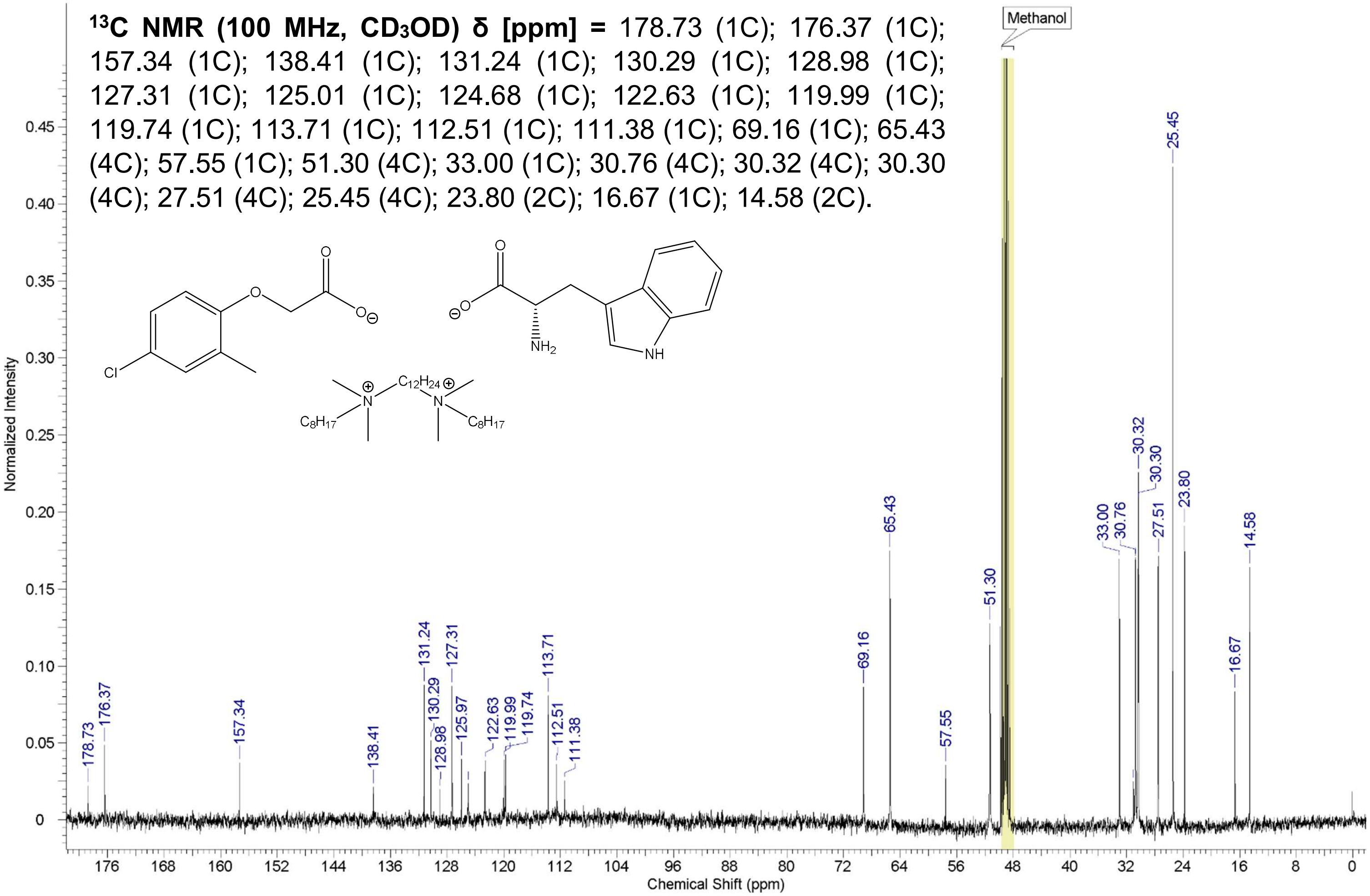

Figure S32. ^{13}C NMR spectrum of L-tryptophanate 4-chloro-2-methylphenoxyacetate dodecylmethylene-1,12-bis(octadimethylammonium) (**11**)

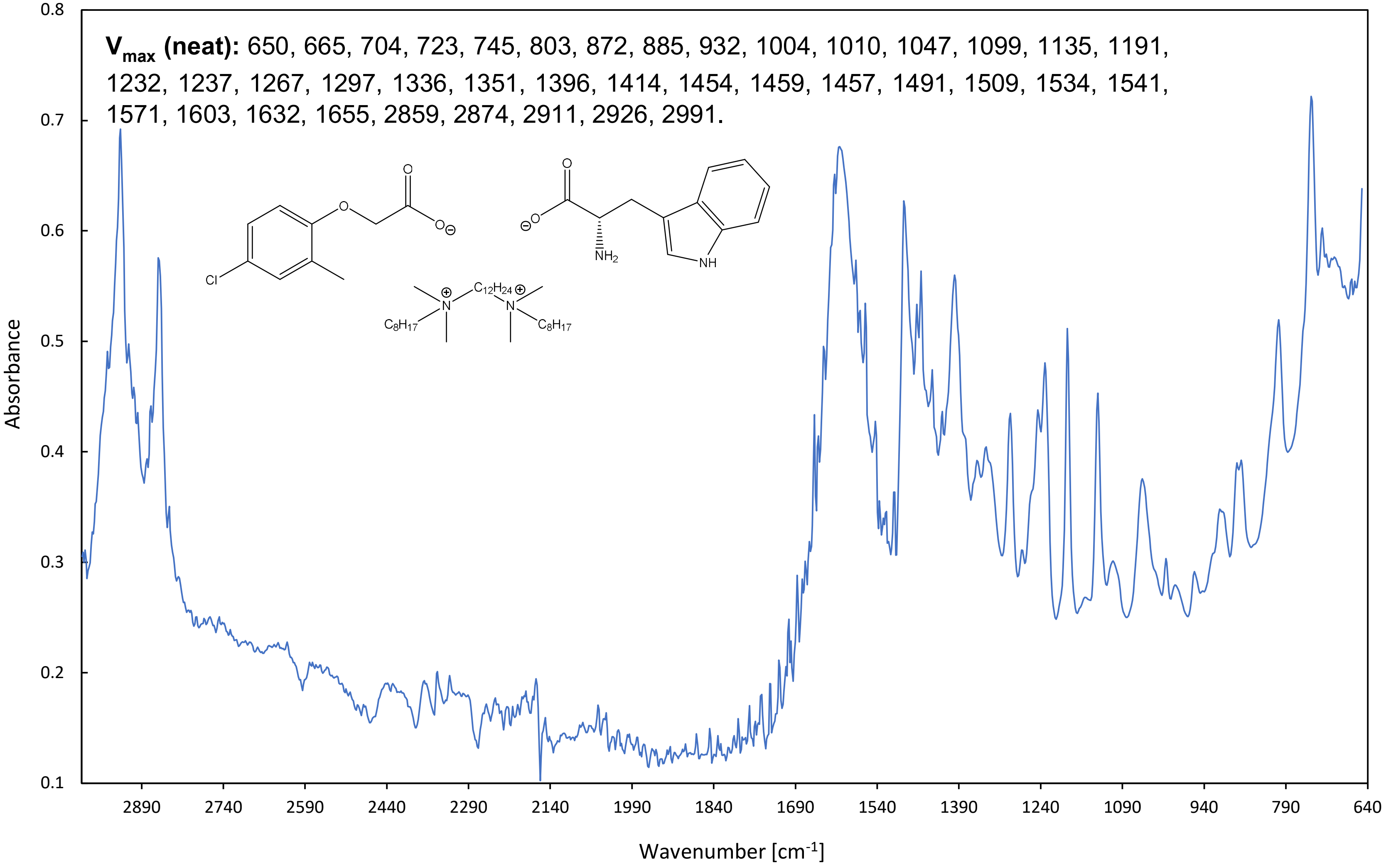

Figure S33. FT-IR spectrum of L-tryptophanate 4-chloro-2-methylphenoxyacetate dodecylmethylene-1,12-bis(octadimethylammonium) (**11**)

File Name						***Frequency (MHz)***	399.91
Nucleus	1H	***Number of Transients***	64	***Original Points Count***	38889	***Points Count***	65536
Pulse Sequence	s2pul	***Receiver Gain***	22.00	***Solvent***	METHANOL-d4	***Spectrum Offset (Hz)***	2462.7456
Spectrum Type	STANDARD	***Sweep Width (Hz)***	5555.56	***Temperature (degree C)***	AMBIENT TEMPERATURE		

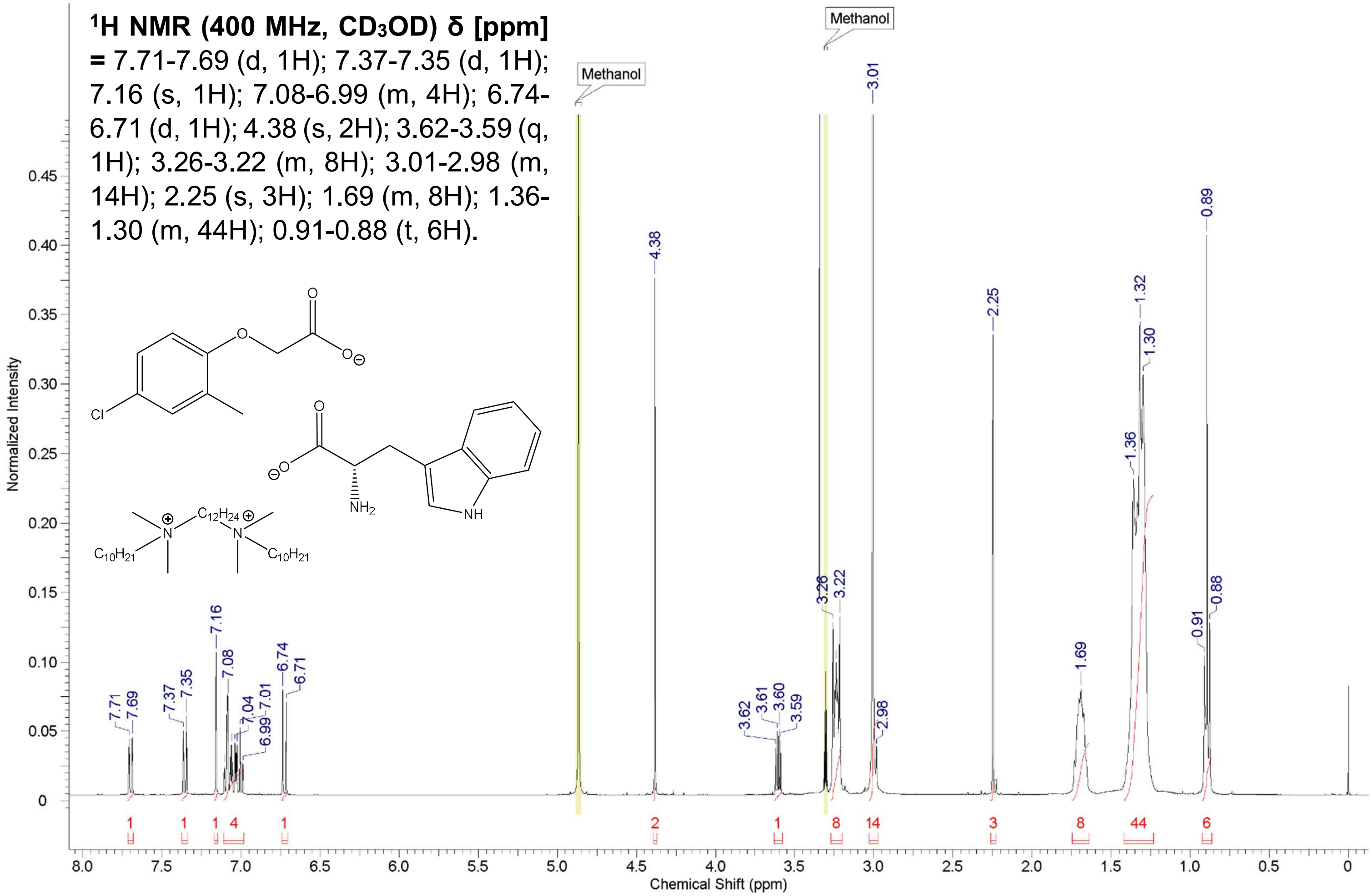

Figure S34. ^{1}H NMR spectrum of L-tryptophanate 4-chloro-2-methylphenoxyacetate dodecylmethylene-1,12-bis(decyldimethylammonium) (**12**)

File Name						Frequency (MHz)	100.57
Nucleus	13C	Number of Transients	1724	Original Points Count	33263	Points Count	65536
Pulse Sequence	s2pul	Receiver Gain	54.00	Solvent	METHANOL-d4	Spectrum Offset (Hz)	9749.8506
Spectrum Type	STANDARD	Sweep Width (Hz)	21186.44	Temperature (degree C)	AMBIENT TEMPERATURE		

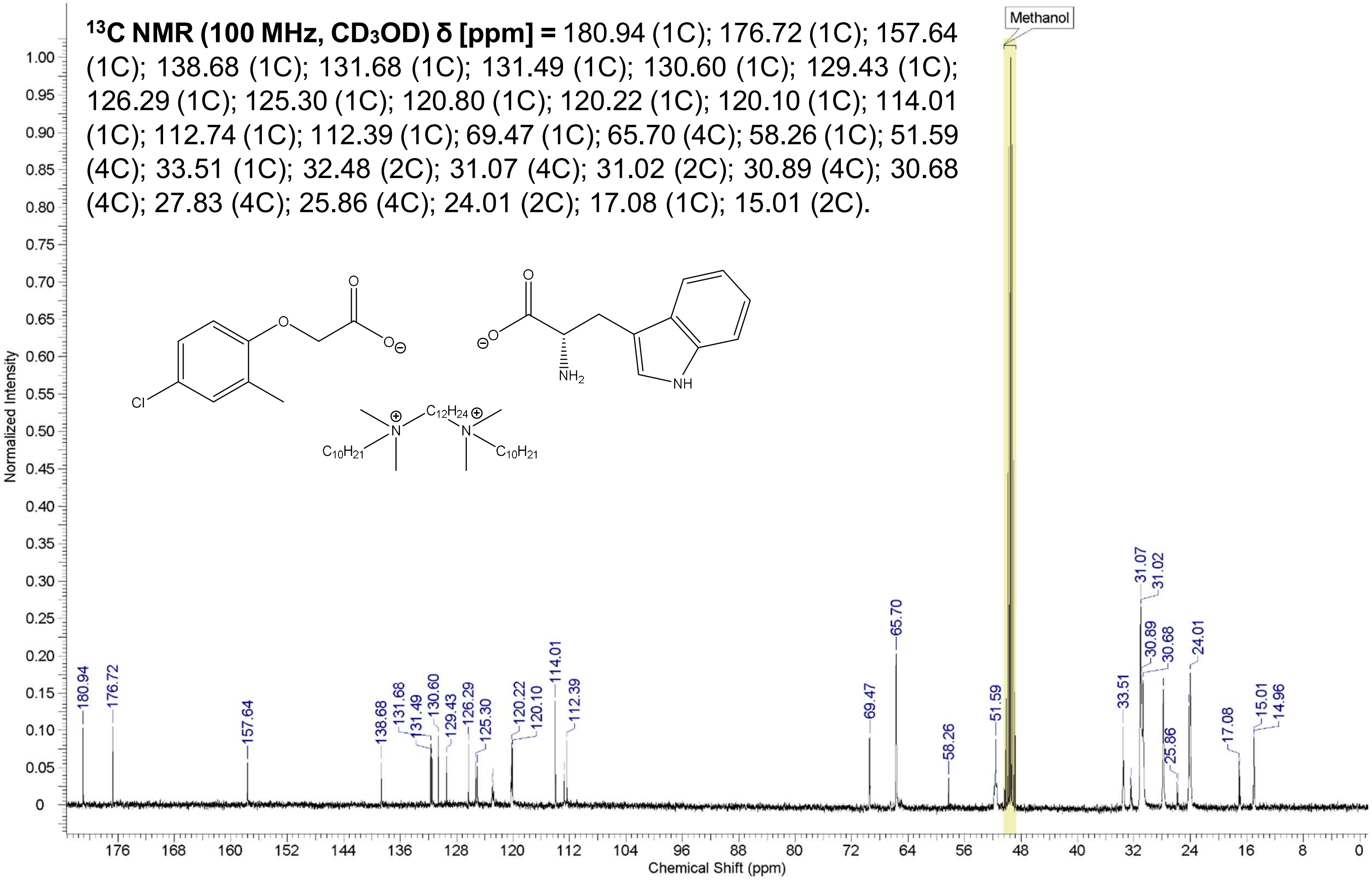

Figure S35. ^{13}C NMR spectrum of L-try ptophanate 4-chloro-2-methylphenoxyacetate dodecylmethylene-1,12-bis(decyldimethylammonium) (**12**)

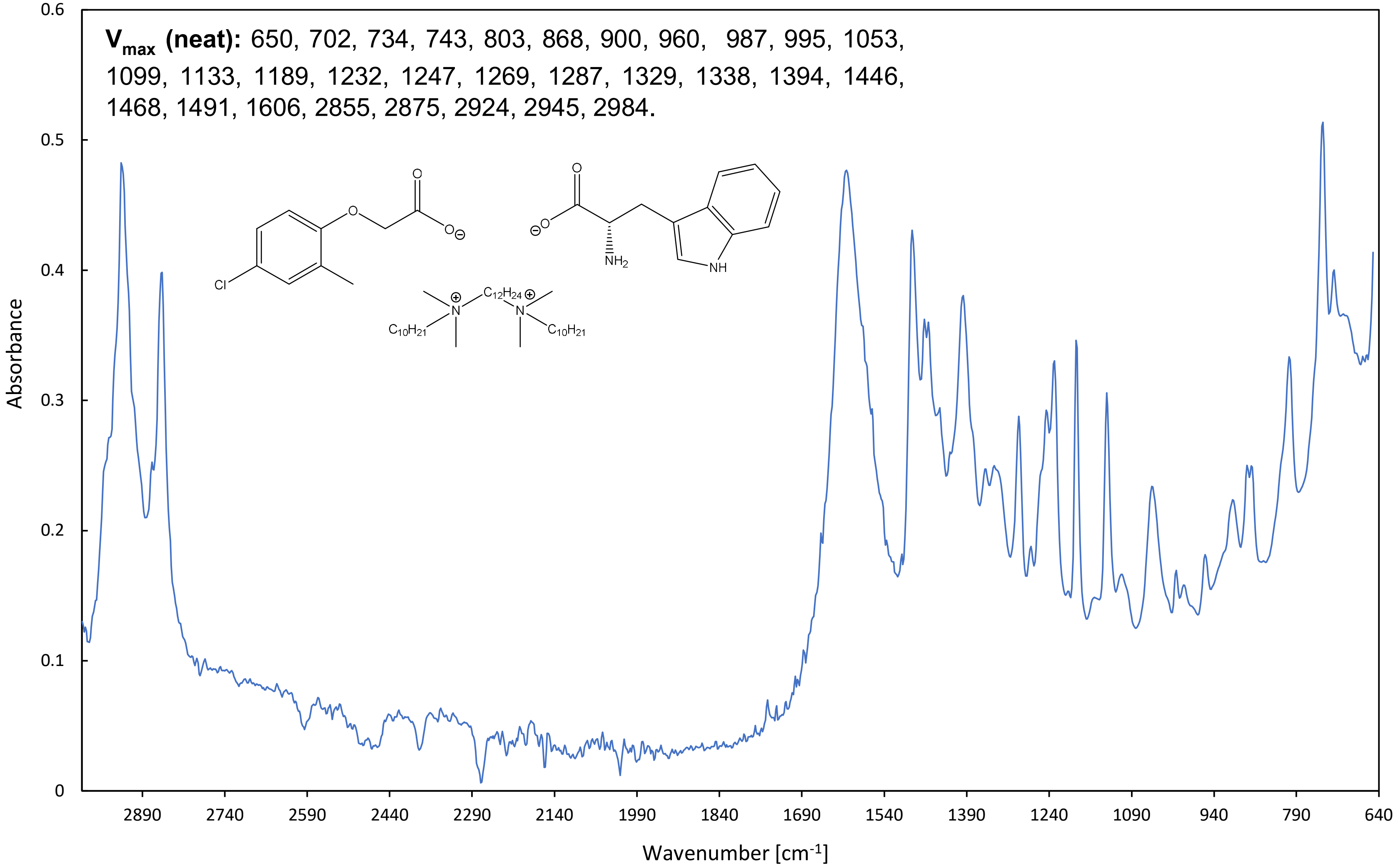

Figure S36. FT-IR spectrum of L-tryptophanate 4-chloro-2-methylphenoxyacetate dodecylmethylene-1,12-bis(decyldimethylammonium) (**12**)

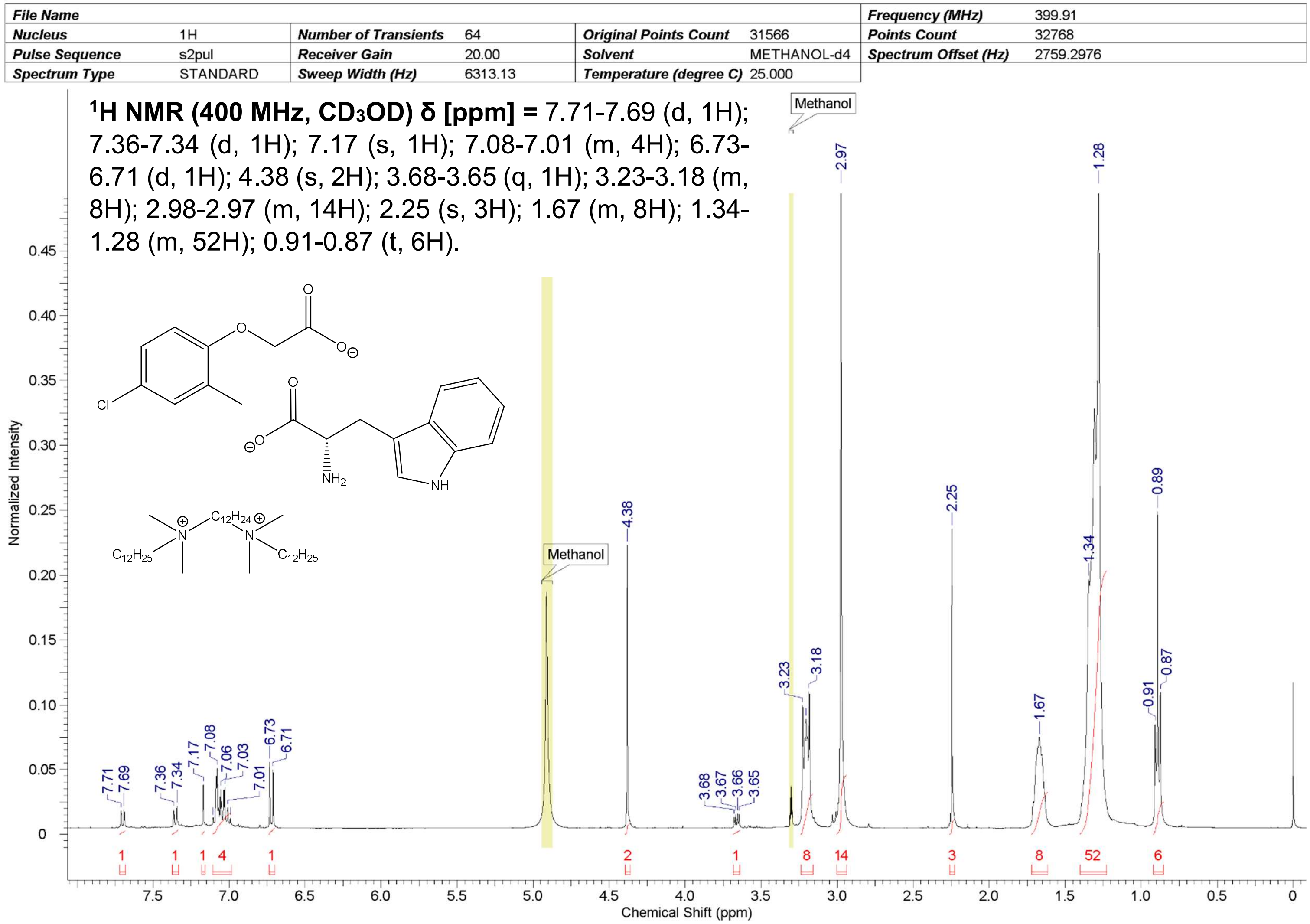

Figure S37. ^{1}H NMR spectrum of L-tryptophanate 4-chloro-2-methylphenoxyacetate dodecylmethylene-1,12-bis(dodecyldimethylammonium) (**13**)

File Name						Frequency (MHz)	100.57
Nucleus	13C	Number of Transients	1964	Original Points Count	26316	Points Count	32768
Pulse Sequence	s2pul	Receiver Gain	58.00	Solvent	METHANOL-d4	Spectrum Offset (Hz)	9908.0918
Spectrum Type	STANDARD	Sweep Width (Hz)	21929.82	Temperature (degree C)	25.000		

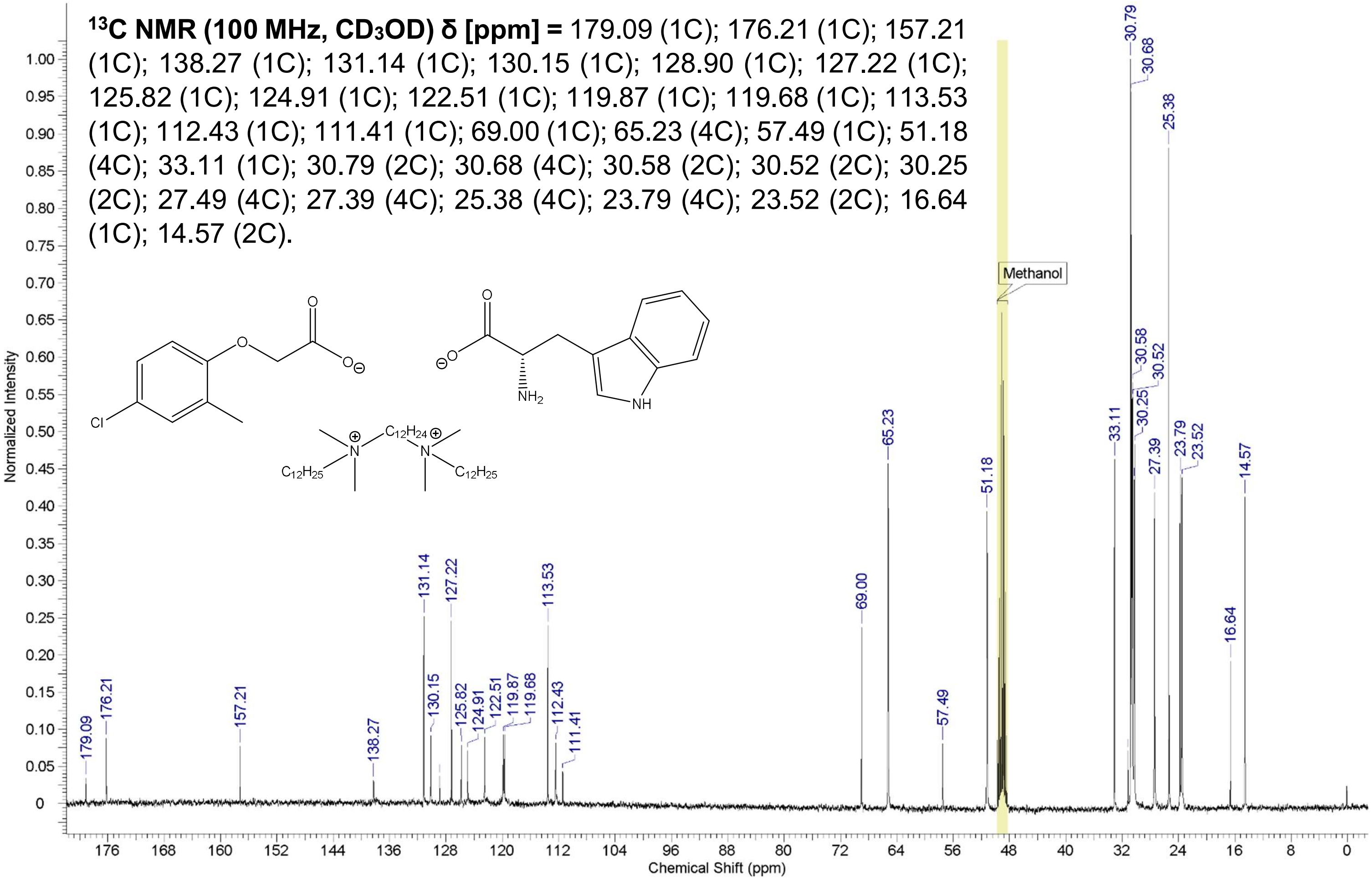

Figure S38. ^{13}C NMR spectrum of L-tryptophanate 4-chloro-2-methylphenoxyacetate dodecylmethylene-1,12-bis(dodecyldimethylammonium) (**13**)

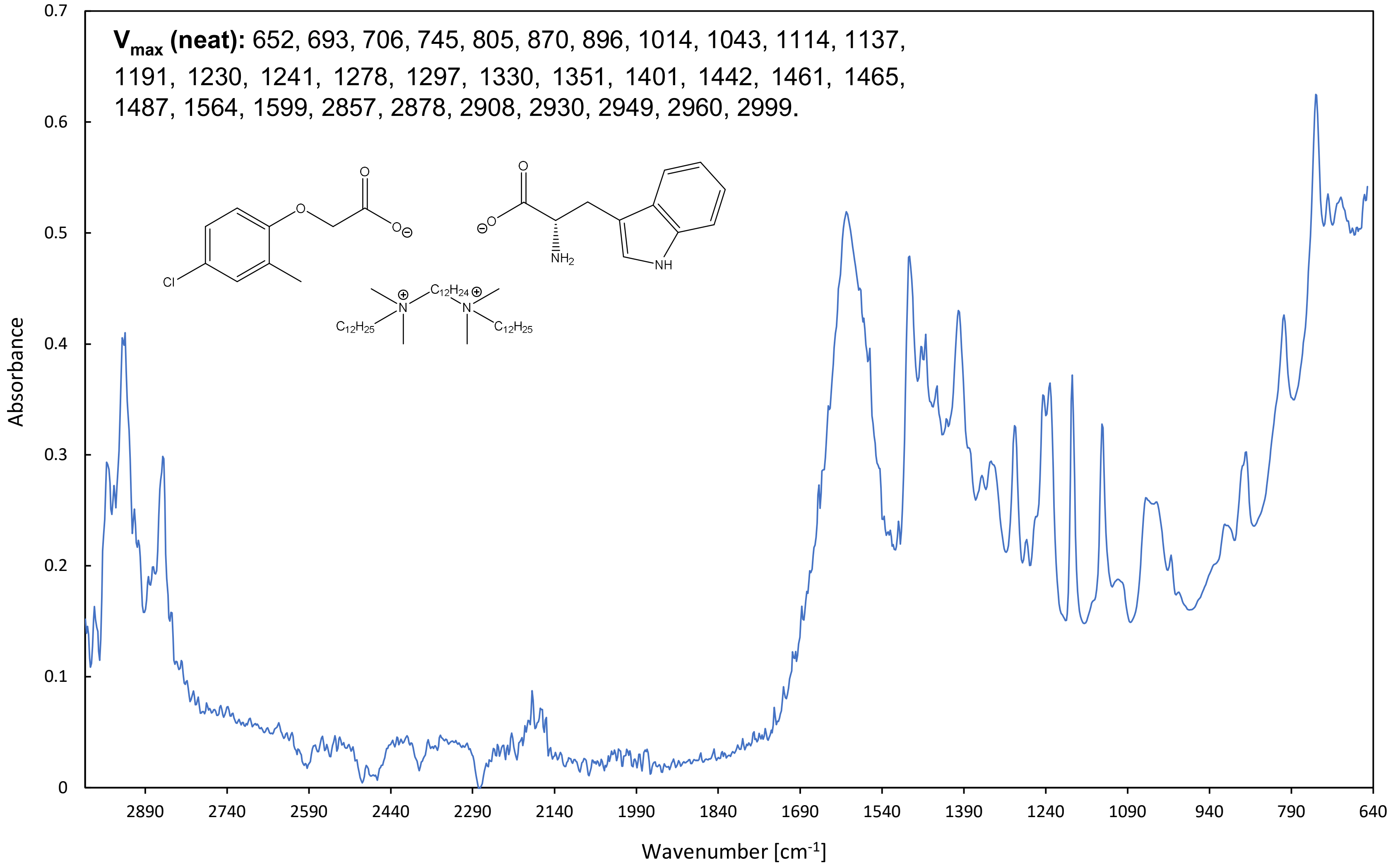

Figure S39. FT-IR spectrum of L-tryptophanate 4-chloro-2-methylphenoxyacetate dodecylmethylene-1,12-bis(dodecyldimethylammonium) (**13**)

File Name						Frequency (MHz)	399.91
Nucleus	1H	Number of Transients	64	Original Points Count	30049	Points Count	32768
Pulse Sequence	s2pul	Receiver Gain	18.00	Solvent	METHANOL-d4	Spectrum Offset (Hz)	2616.0938
Spectrum Type	STANDARD	Sweep Width (Hz)	6009.62	Temperature (degree C)	25.000		

^{1}H NMR (400 MHz, CD$_3$OD) δ [ppm] = 7.71-7.69 (d, 1H); 7.36-7.34 (d, 1H); 7.16 (s, 1H); 7.08-7.03 (m, 4H); 6.73-6.71 (d, 1H); 4.38 (s, 2H); 3.67-3.63 (q, 1H); 3.22-3.18 (m, 8H); 2.98-2.97 (m, 14H); 2.24 (s, 3H); 1.68 (m, 8H); 1.31-1.28 (m, 60H); 0.91-0.87 (t, 6H).

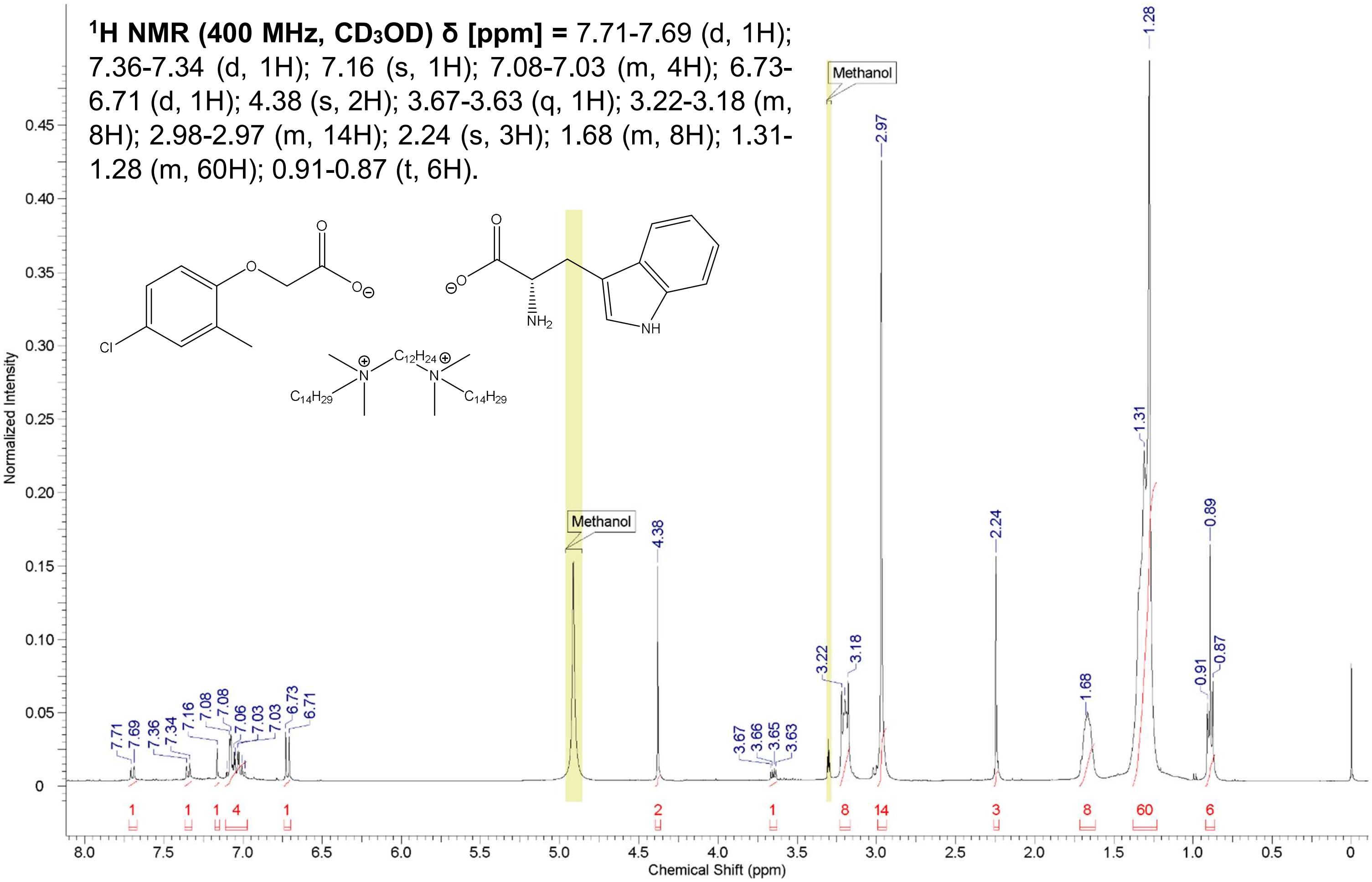

Figure S40. ^{1}H NMR spectrum of L-tryptophanate 4-chloro-2-methylphenoxyacetate dodecylmethylene-1,12-bis(tetradecyldimethylammonium) (**14**)

File Name						Frequency (MHz)	100.57
Nucleus	13C	Number of Transients	28796	Original Points Count	26316	Points Count	32768
Pulse Sequence	s2pul	Receiver Gain	58.00	Solvent	METHANOL-d4	Spectrum Offset (Hz)	9908.4258
Spectrum Type	STANDARD	Sweep Width (Hz)	21929.82	Temperature (degree C)	25.000		

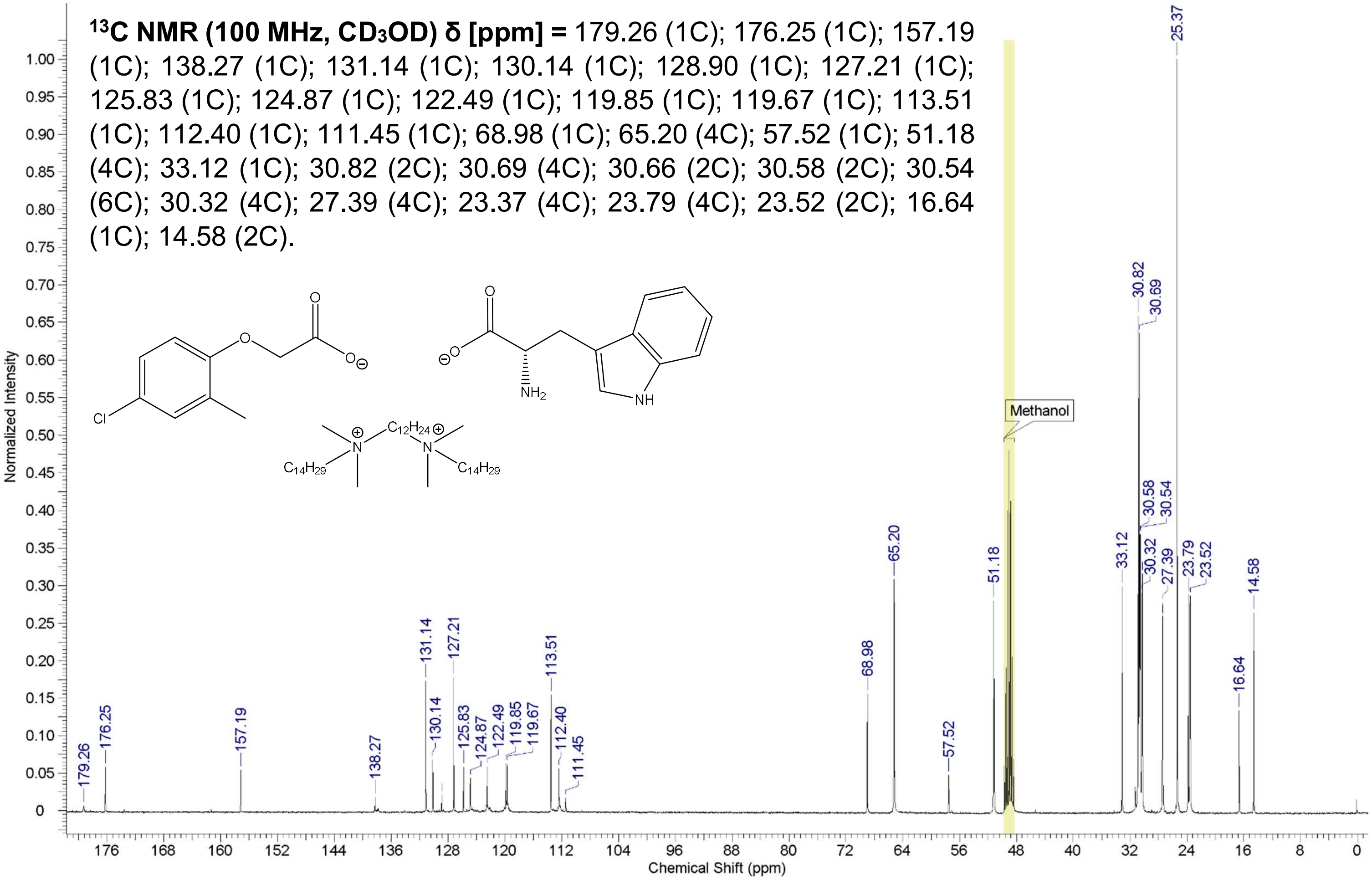

Figure S41. ^{13}C NMR spectrum of L-tryptophanate 4-chloro-2-methylphenoxyacetate dodecylmethylene-1,12-bis(tetradecyldimethylammonium) (**14**)

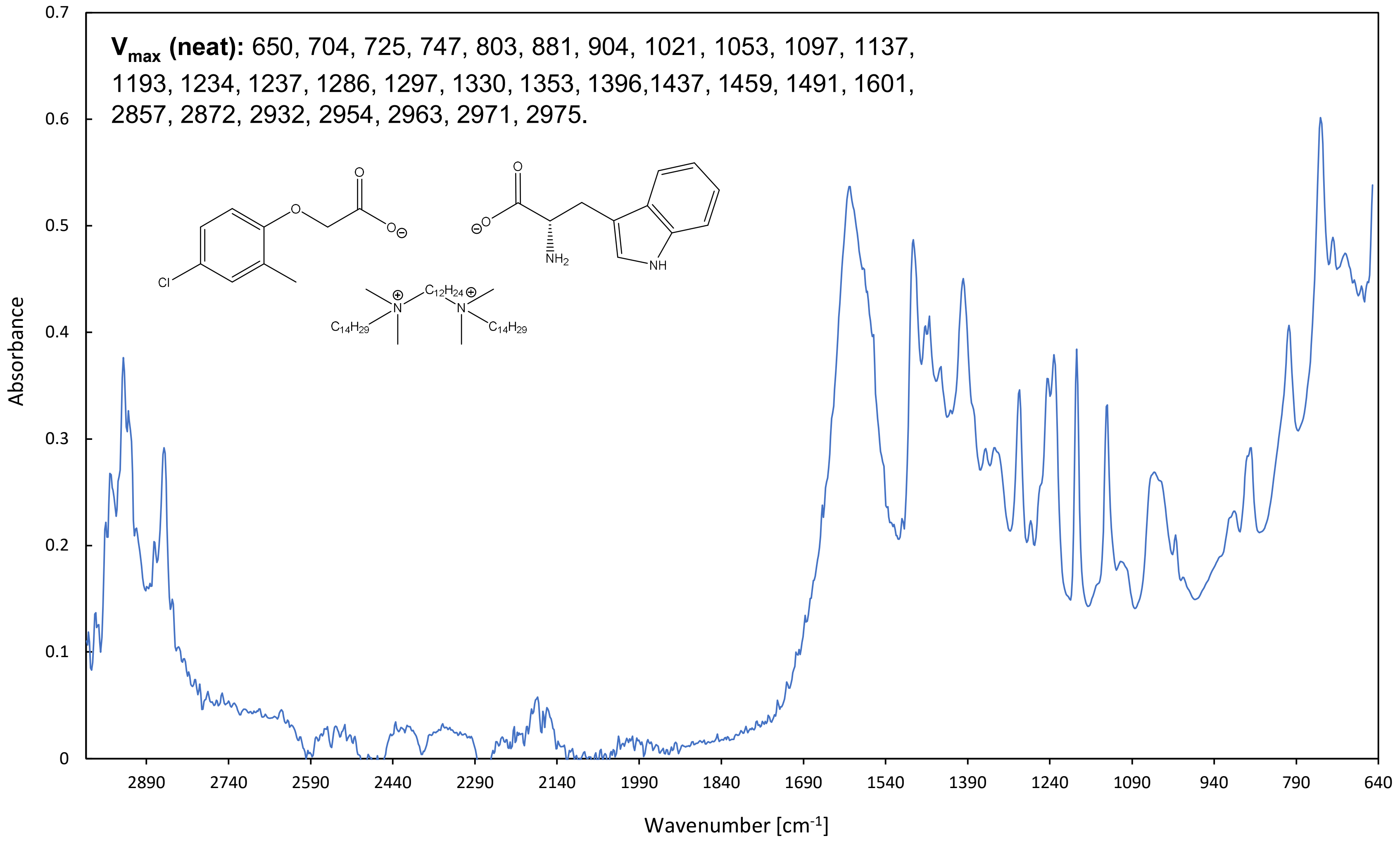

Figure S42. FT-IR spectrum of L-tryptophanate 4-chloro-2-methylphenoxyacetate dodecylmethylene-1,12-bis(tetradecyldimethylammonium) (**14**)

File Name						Frequency (MHz)	399.91
Nucleus	1H	Number of Transients	64	Original Points Count	30049	Points Count	32768
Pulse Sequence	s2pul	Receiver Gain	20.00	Solvent	METHANOL-d4	Spectrum Offset (Hz)	2616.3179
Spectrum Type	STANDARD	Sweep Width (Hz)	6009.62	Temperature (degree C)	25.000		

^{1}H NMR (400 MHz, CD_3OD) δ [ppm] = 7.71-7.69 (d, 1H); 7.36-7.34 (d, 1H); 7.16 (s, 1H); 7.08-7.03 (m, 4H); 6.73-6.71 (d, 1H); 4.38 (s, 2H); 3.68-3.65 (q, 1H); 3.24-3.19 (m, 8H); 2.99-2.98 (m, 14H); 2.24 (s, 3H); 1.68 (m, 8H); 1.35-1.28 (m, 68H); 0.91-0.88 (t, 6H).

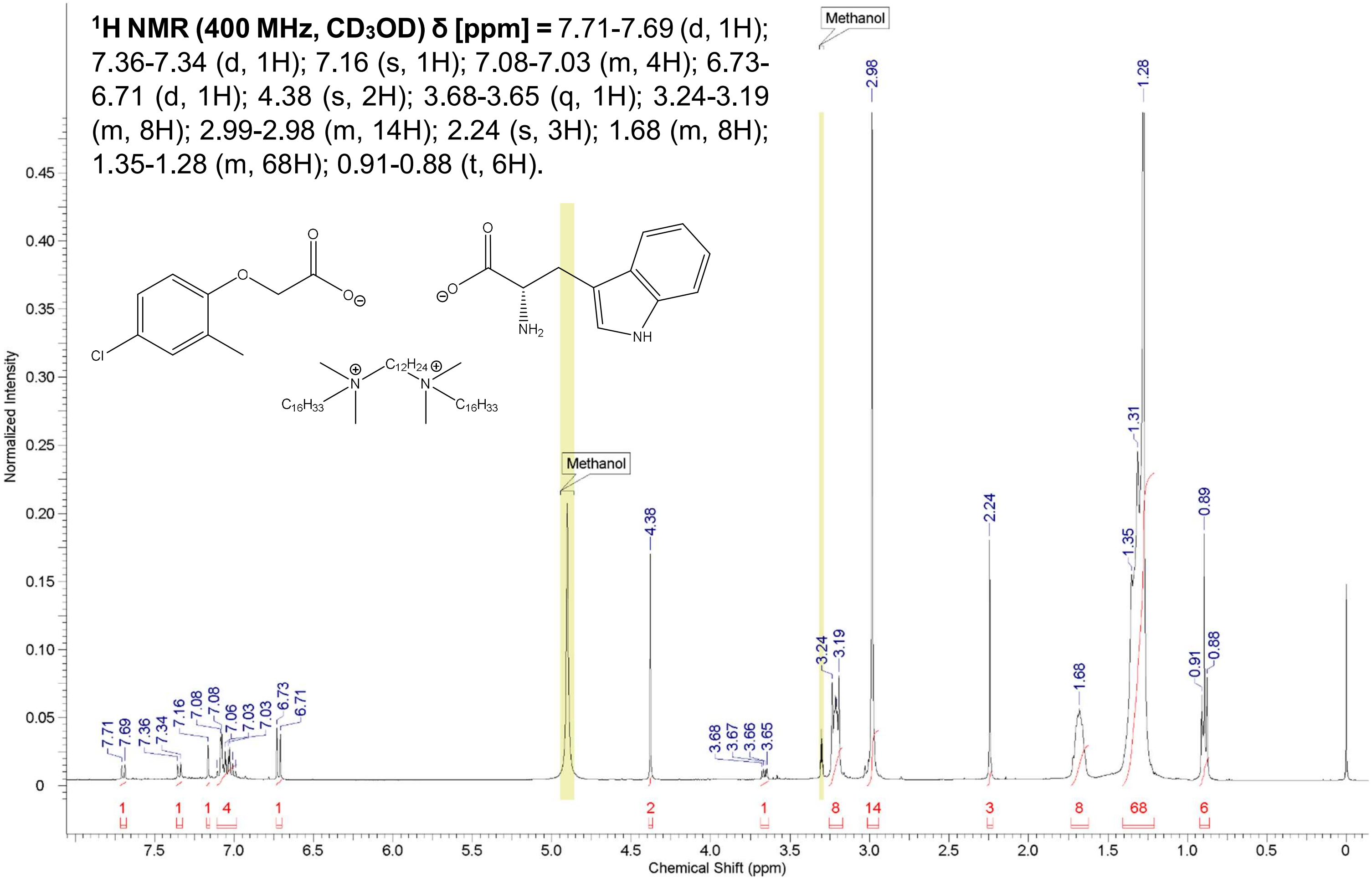

Figure S43. ^{1}H NMR spectrum of L-tryptophanate 4-chloro-2-methylphenoxyacetate dodecylmethylene-1,12-bis(hexadecyldimethylammonium) (**15**)

File Name						Frequency (MHz)	100.57
Nucleus	13C	Number of Transients	1024	Original Points Count	26316	Points Count	32768
Pulse Sequence	s2pul	Receiver Gain	58.00	Solvent	METHANOL-d4	Spectrum Offset (Hz)	9909.0957
Spectrum Type	STANDARD	Sweep Width (Hz)	21929.82	Temperature (degree C)	25.000		

^{13}C NMR (100 MHz, CD_3OD) δ [ppm] = 179.08 (1C); 176.28 (1C); 157.20 (1C); 138.28 (1C); 131.14 (1C); 130.15 (1C); 128.89 (1C); 127.21 (1C); 125.84 (1C); 124.86 (1C); 122.50 (1C); 119.86 (1C); 119.65 (1C); 113.51 (1C); 112.39 (1C); 111.37 (1C); 68.98 (1C); 65.23 (4C); 57.50 (1C); 51.20 (4C); 33.12 (1C); 30.85 (2C); 30.69 (4C); 30.64 (2C); 30.54 (2C); 30.32 (10C); 27.40 (4C); 25.35 (4C); 23.80 (4C); 23.53 (4C); 23.50 (2C); 16.63 (1C); 14.56 (2C).

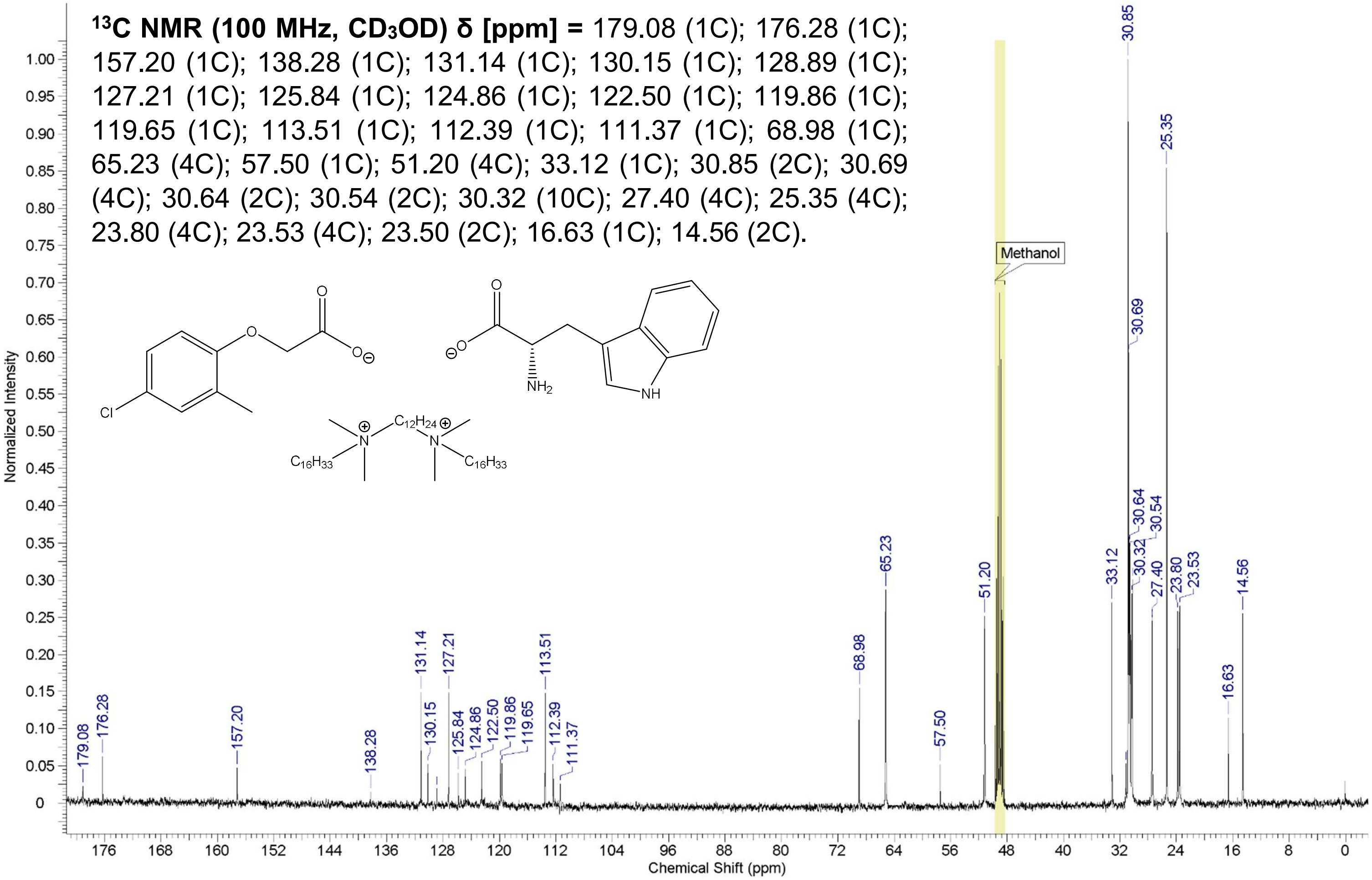

Figure S44. ^{13}C NMR spectrum of L-tryptophanate 4-chloro-2-methylphenoxyacetate dodecylmethylene-1,12-bis(hexadecyldimethylammonium) (**15**)

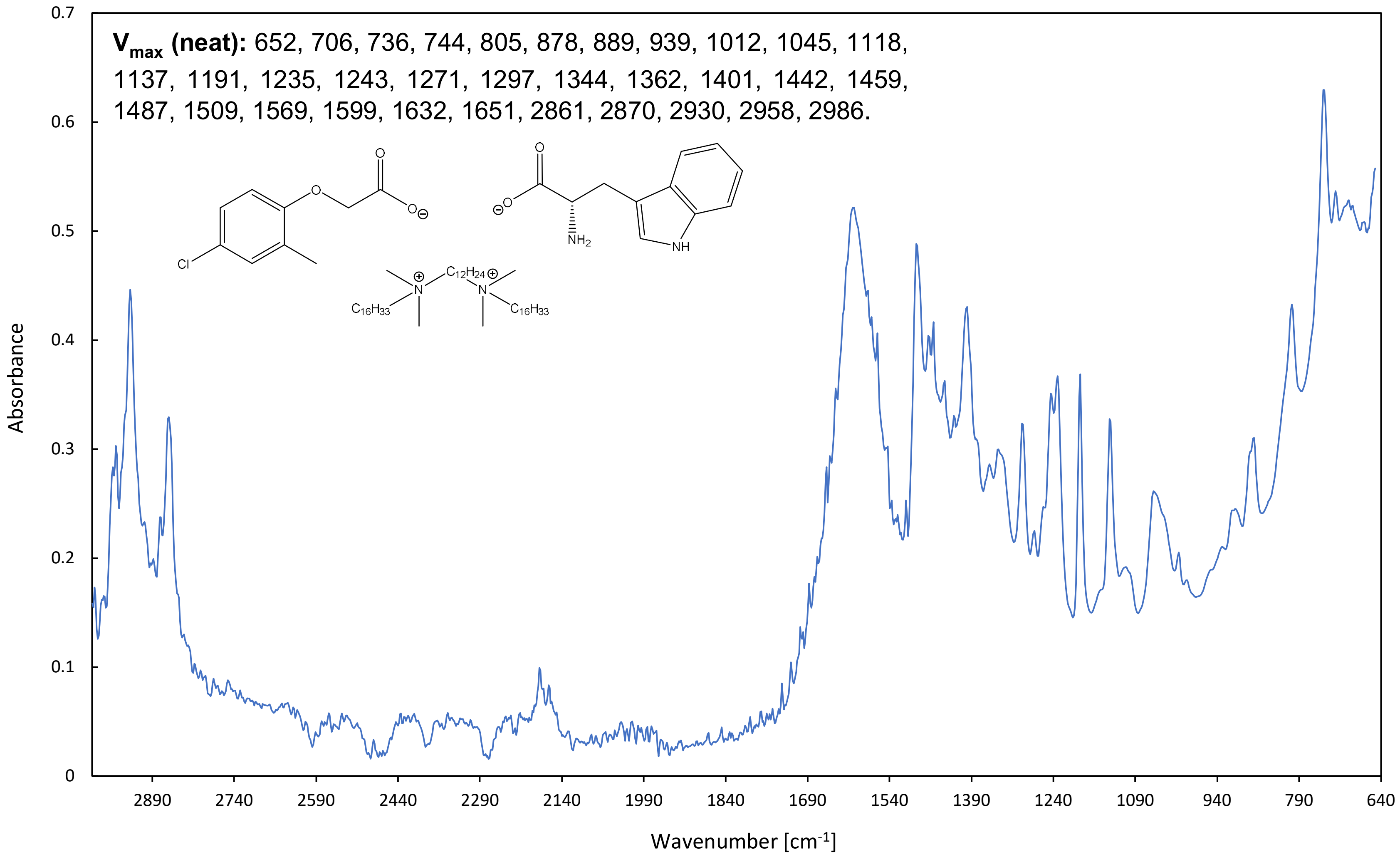

Figure S45. FT-IR spectrum of L-tryptophanate 4-chloro-2-methylphenoxyacetate dodecylmethylene-1,12-bis(hexadecyldimethylammonium) (**15**)

File Name						*Frequency (MHz)*	399.91
Nucleus	1H	*Number of Transients*	128	*Original Points Count*	16523	*Points Count*	32768
Pulse Sequence	s2pul	*Receiver Gain*	16.00	*Solvent*	METHANOL-d4	*Spectrum Offset (Hz)*	1350.6299
Spectrum Type	STANDARD	*Sweep Width (Hz)*	3304.69	*Temperature (degree C)*	AMBIENT TEMPERATURE		

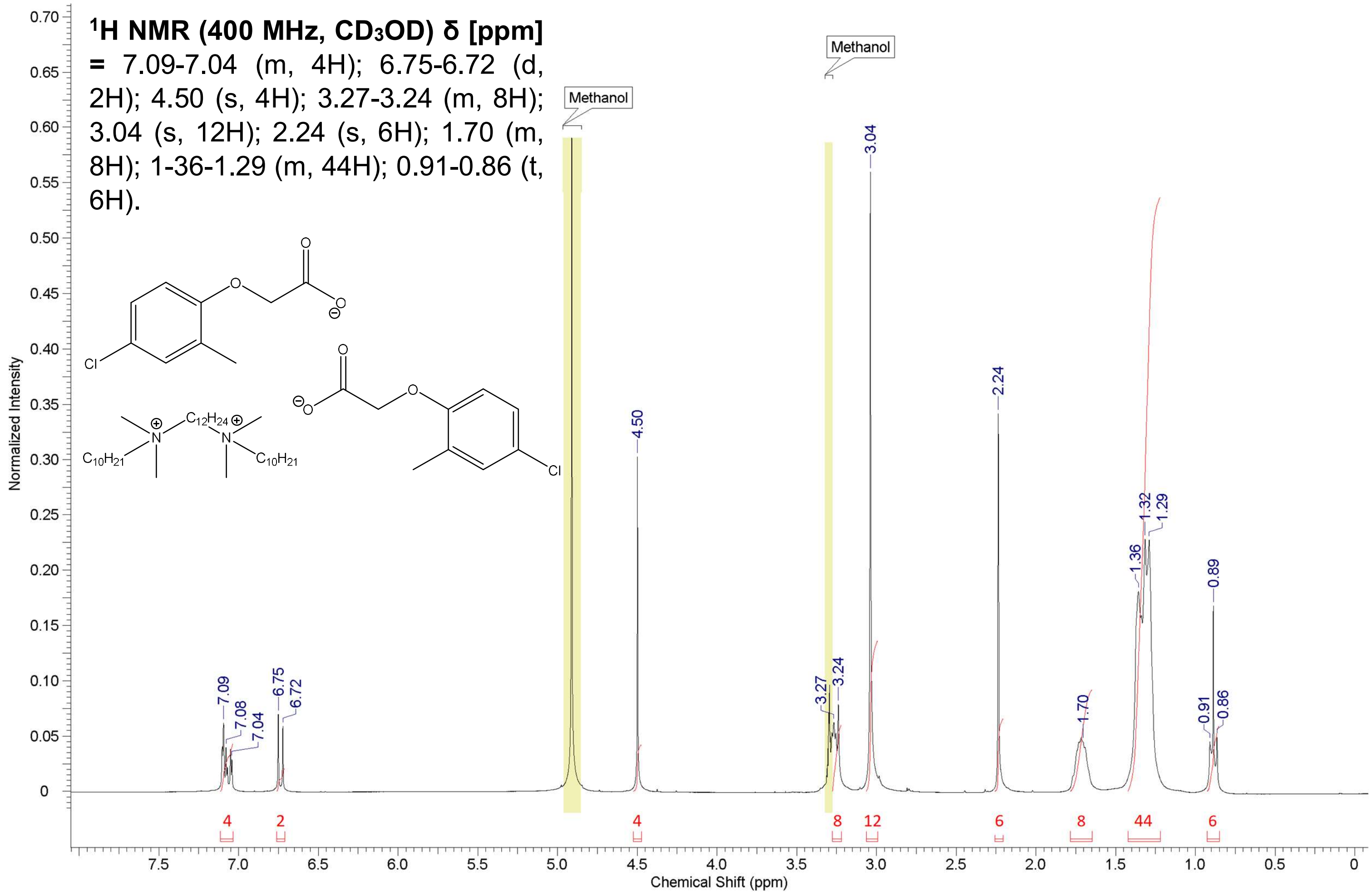

Figure S46. ^{1}H NMR spectrum of 4-chloro-2-methylphenoxyacetate dodecylmethylene-1,12-bis(decyldimethylammonium) (**12a**)

File Name						*Frequency (MHz)*	100.57
Nucleus	13C	*Number of Transients*	2348	*Original Points Count*	32769	*Points Count*	65536
Pulse Sequence	s2pul	*Receiver Gain*	34.00	*Solvent*	METHANOL-d4	*Spectrum Offset (Hz)*	7279.3154
Spectrum Type	STANDARD	*Sweep Width (Hz)*	17391.30	*Temperature (degree C)*	AMBIENT TEMPERATURE		

^{13}C NMR (100 MHz, CD_3OD) δ [ppm] = 174.59 (2C); 156.84 (2C); 131.26 (2C); 130.21 (2C); 127.30 (2C); 126.22 (2C); 113.56 (2C); 67.81 (2C) 65.30 (4C); 51.23 (4C); 33.07 (2C); 30.63 (4C); 30.60 (2C); 30.56 (2C); 30.47 (2C); 30.40 (4C); 27.40 (4C); 23.81 (4C); 23.76 (2C); 16.51 (2C); 14.53 (2C).

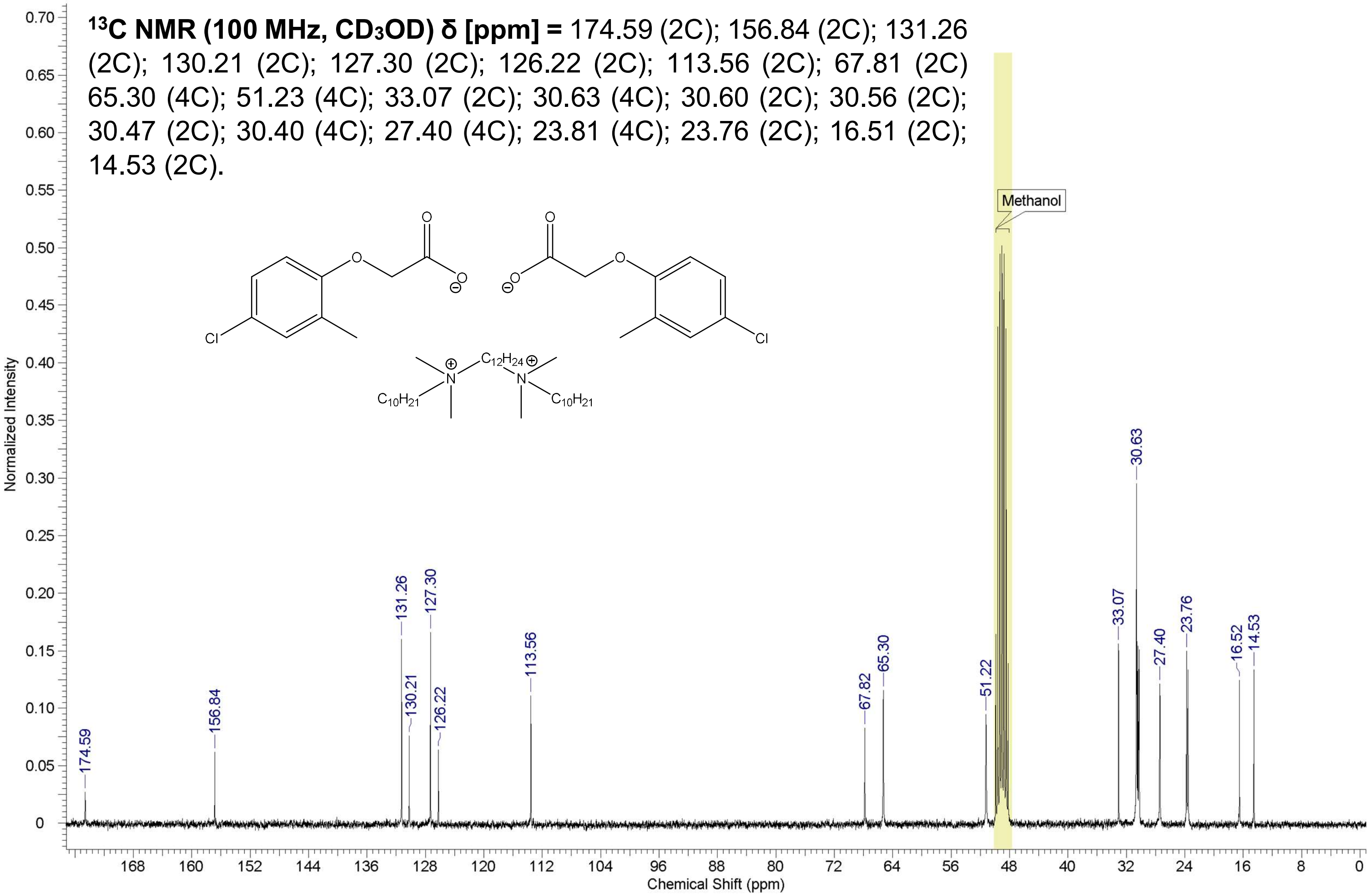

Figure S47. ^{13}C NMR spectrum of 4-chloro-2-methylphenoxyacetate dodecylmethylene-1,12-bis(decyldimethylammonium) (**12a**)

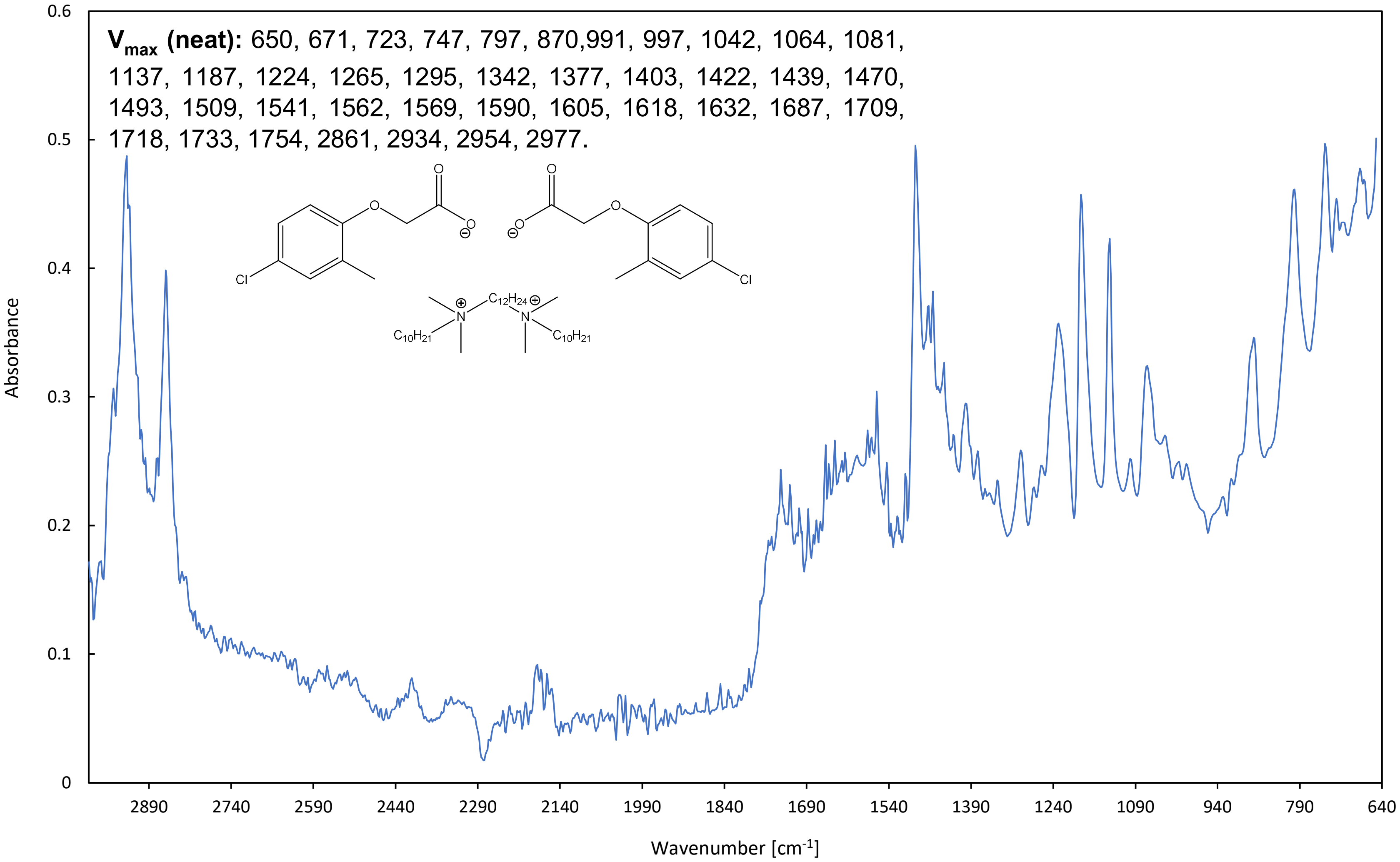

Figure S48. FT-IR spectrum of 4-chloro-2-methylphenoxyacetate dodecylmethylene-1,12-bis(hexadecyldimethylammonium) (**12a**)

File Name						***Frequency (MHz)***	399.91
Nucleus	1H	***Number of Transients***	64	***Original Points Count***	24814	***Points Count***	32768
Pulse Sequence	s2pul	***Receiver Gain***	12.00	***Solvent***	METHANOL-d4	***Spectrum Offset (Hz)***	1231.6826
Spectrum Type	STANDARD	***Sweep Width (Hz)***	3544.84	***Temperature (degree C)***	AMBIENT TEMPERATURE		

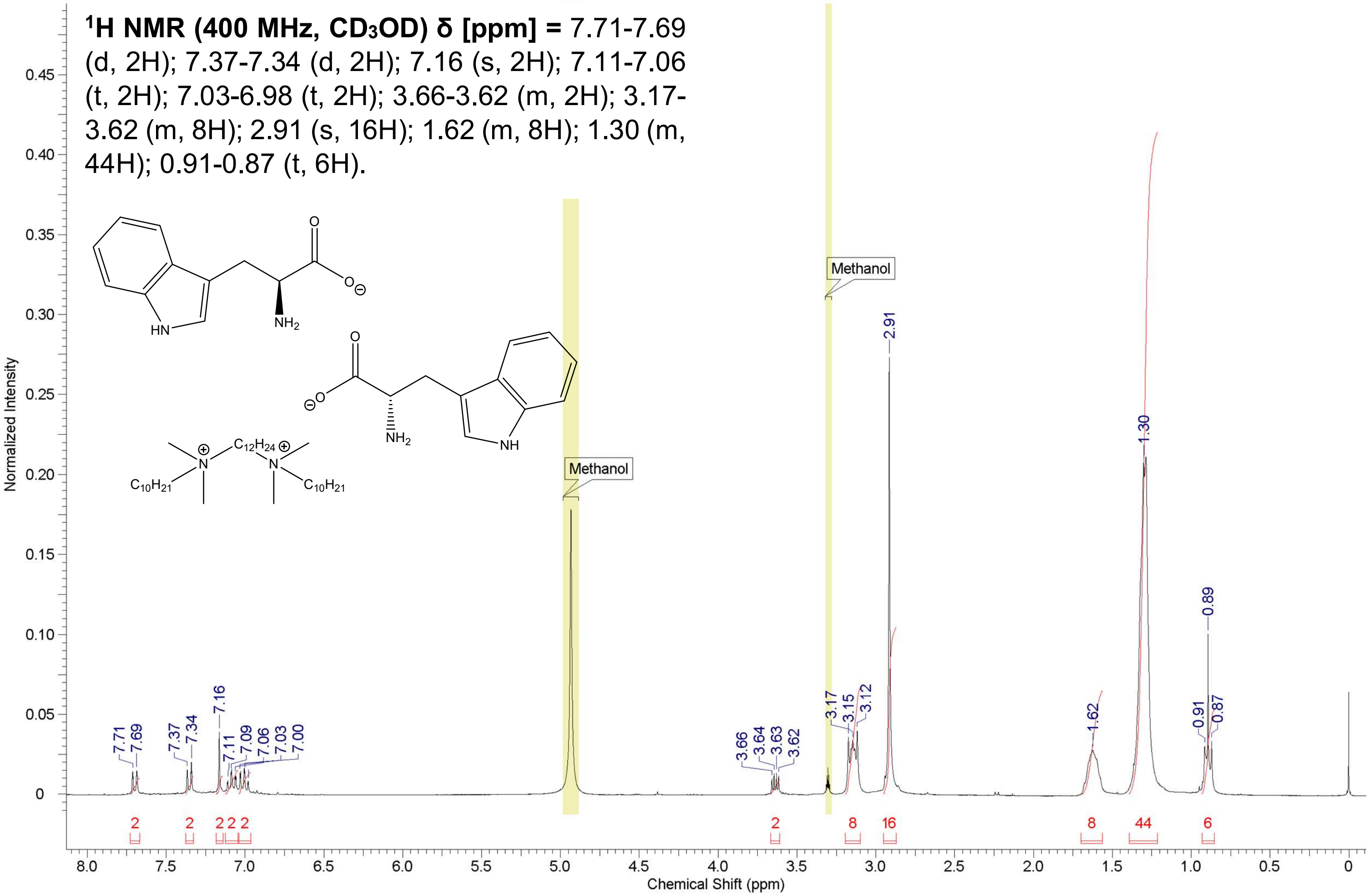

Figure S49. ^{1}H NMR spectrum of L-tryptophanate dodecylmethylene-1,12-bis(decyldimethylammonium) (**12b**)

File Name						Frequency (MHz)	100.57
Nucleus	13C	Number of Transients	27064	Original Points Count	27451	Points Count	32768
Pulse Sequence	s2pul	Receiver Gain	32.00	Solvent	METHANOL-d4	Spectrum Offset (Hz)	8408.4453
Spectrum Type	STANDARD	Sweep Width (Hz)	19607.84	Temperature (degree C)	AMBIENT TEMPERATURE		

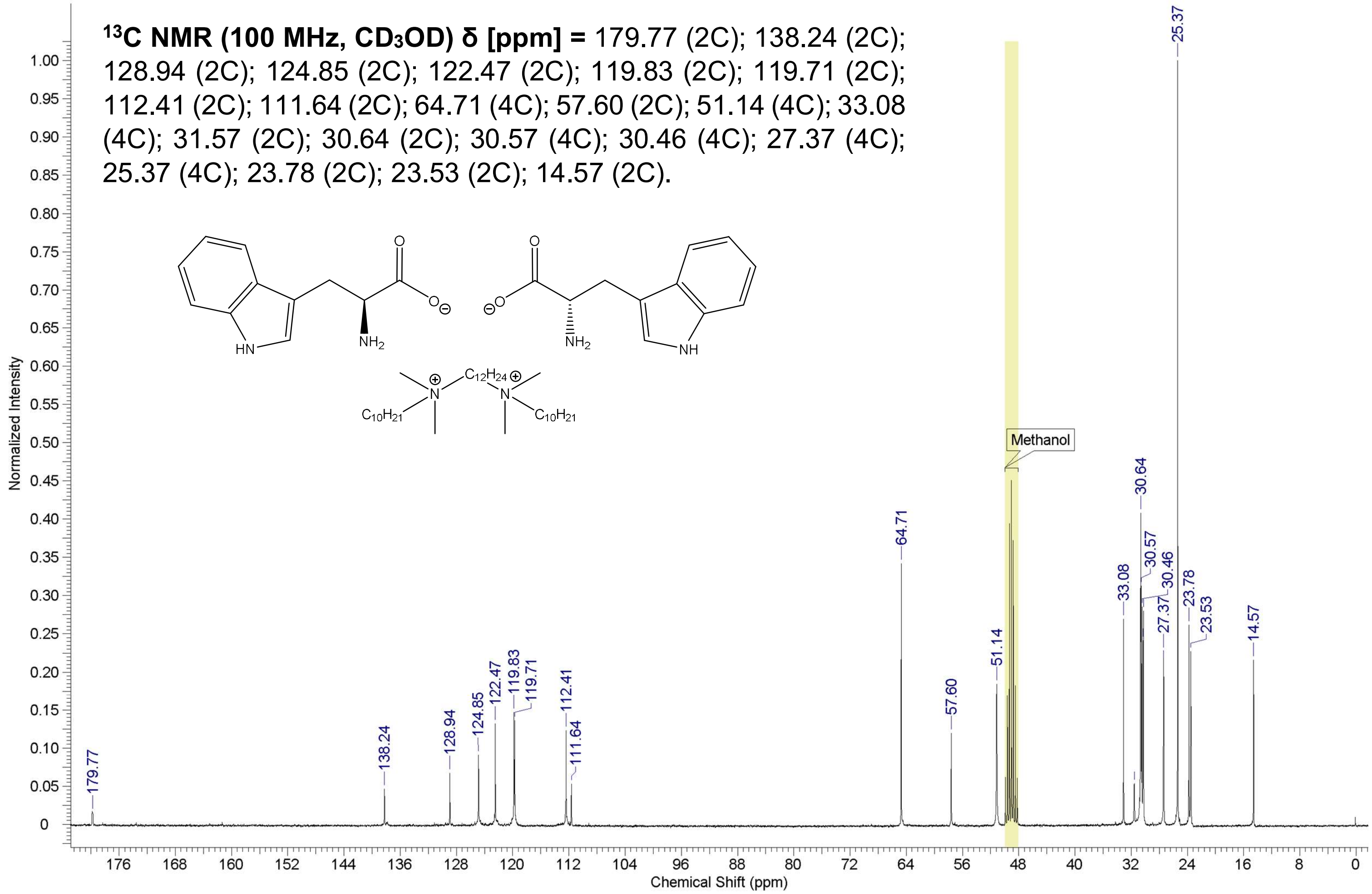

Figure S50. ^{13}C NMR spectrum of L-tryptophanate dodecylmethylene-1,12-bis(decyldimethylammonium) (**12b**)

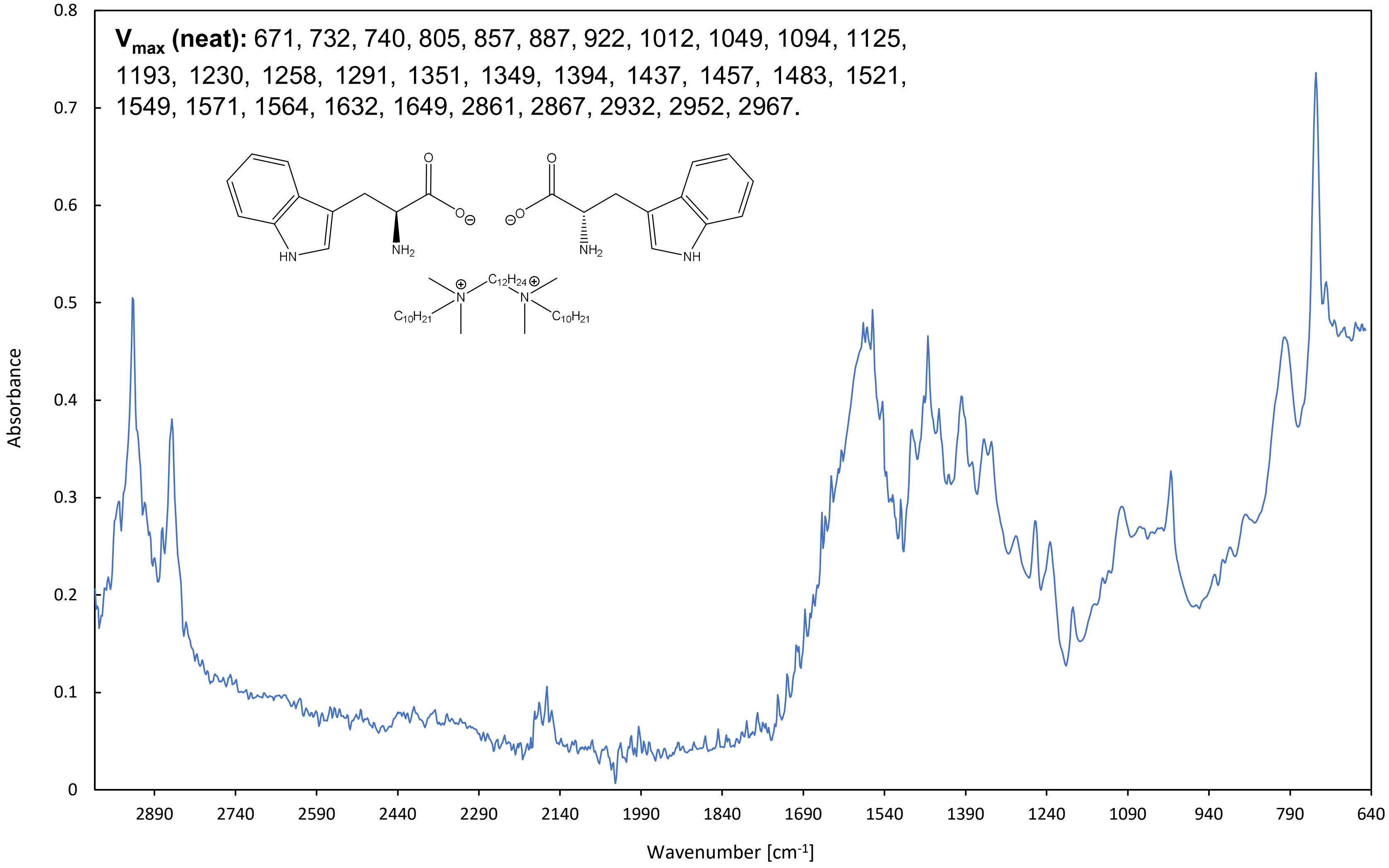

Figure S51. FT-IR spectrum of L-tryptophanate dodecylmethylene-1,12-bis(decyldimethylammonium) (**12b**)

File Name						***Frequency (MHz)***	399.91
Nucleus	1H	***Number of Transients***	64	***Original Points Count***	31566	***Points Count***	32768
Pulse Sequence	s2pul	***Receiver Gain***	18.00	***Solvent***	METHANOL-d4	***Spectrum Offset (Hz)***	2757.8528
Spectrum Type	STANDARD	***Sweep Width (Hz)***	6313.13	***Temperature (degree C)***	25.000		

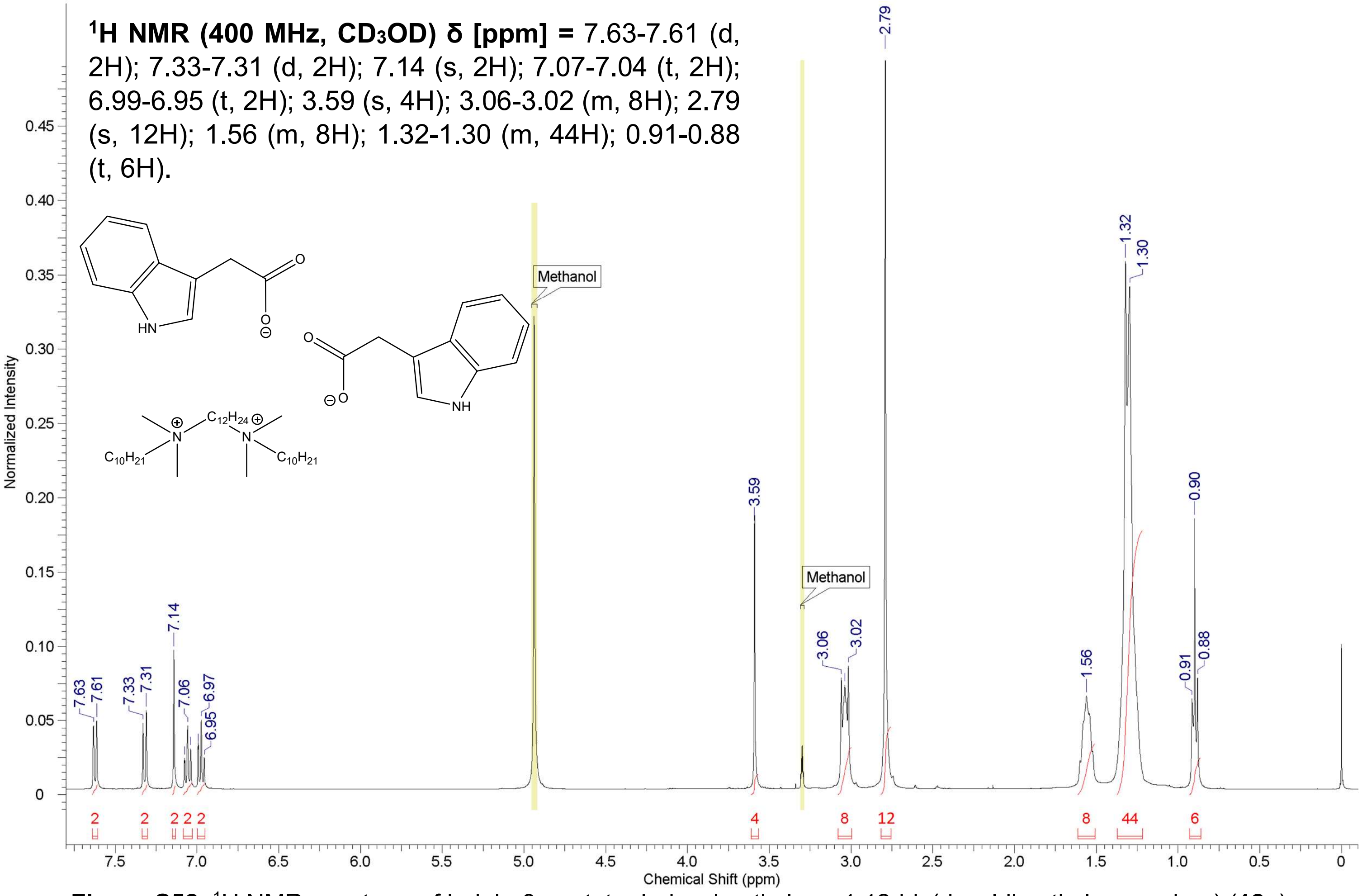

Figure S52. ^{1}H NMR spectrum of indole-3-acetate dodecylmethylene-1,12-bis(decyldimethylammonium) (**12c**)

File Name						Frequency (MHz)	100.57
Nucleus	13C	Number of Transients	1632	Original Points Count	26316	Points Count	32768
Pulse Sequence	s2pul	Receiver Gain	58.00	Solvent	METHANOL-d4	Spectrum Offset (Hz)	9908.7607
Spectrum Type	STANDARD	Sweep Width (Hz)	21929.82	Temperature (degree C)	25.000		

^{13}C NMR (100 MHz, CD_3OD) δ [ppm] = 180.47 (2C); 137.94 (2C); 129.23 (2C); 124.32 (2C); 122.16 (2C); 120.08 (2C); 119.52 (2C); 112.27 (2C); 112.19 (2C); 65.08 (4C); 51.04 (4C); 35.82 (4C); 33.09 (2C); 30.69 (2C); 30.65 (2C); 30.58 (2C); 30.48 (4C); 27.34 (4C); 25.36 (4C); 23.79 (2C); 23.44 (2C); 14.56 (2C).

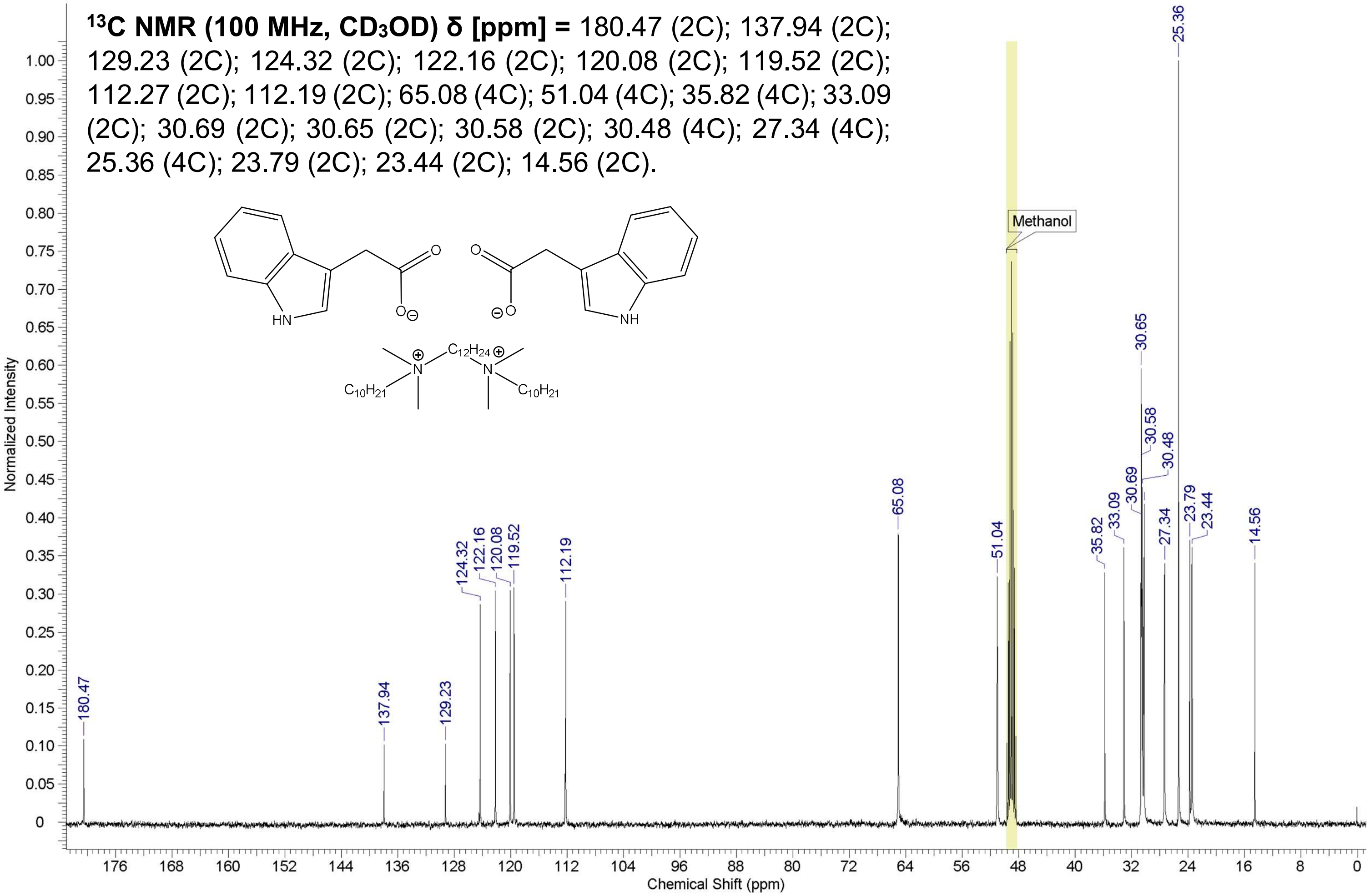

Figure S53. ^{13}C NMR spectrum of indole-3-acetate dodecylmethylene-1,12-bis(decyldimethylammonium) (**12c**)

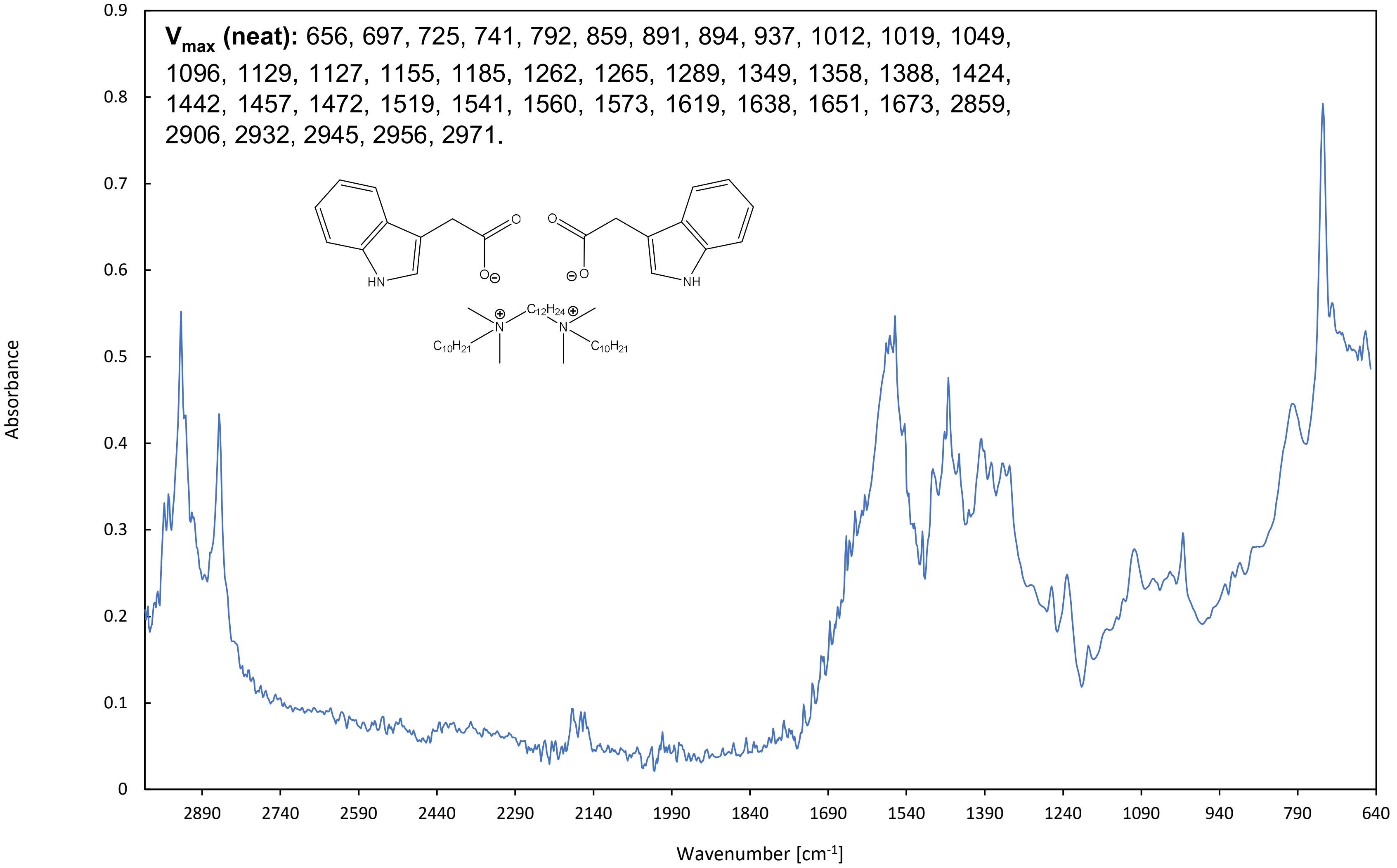

Figure S54. FT-IR spectrum of indole-3-acetate dodecylmethylene-1,12-bis(decyldimethylammonium) (**12c**)

File Name						Frequency (MHz)	399.91
Nucleus	1H	Number of Transients	64	Original Points Count	34091	Points Count	65536
Pulse Sequence	s2pul	Receiver Gain	22.00	Solvent	METHANOL-d4	Spectrum Offset (Hz)	2439.3665
Spectrum Type	STANDARD	Sweep Width (Hz)	5681.82	Temperature (degree C)	25.000		

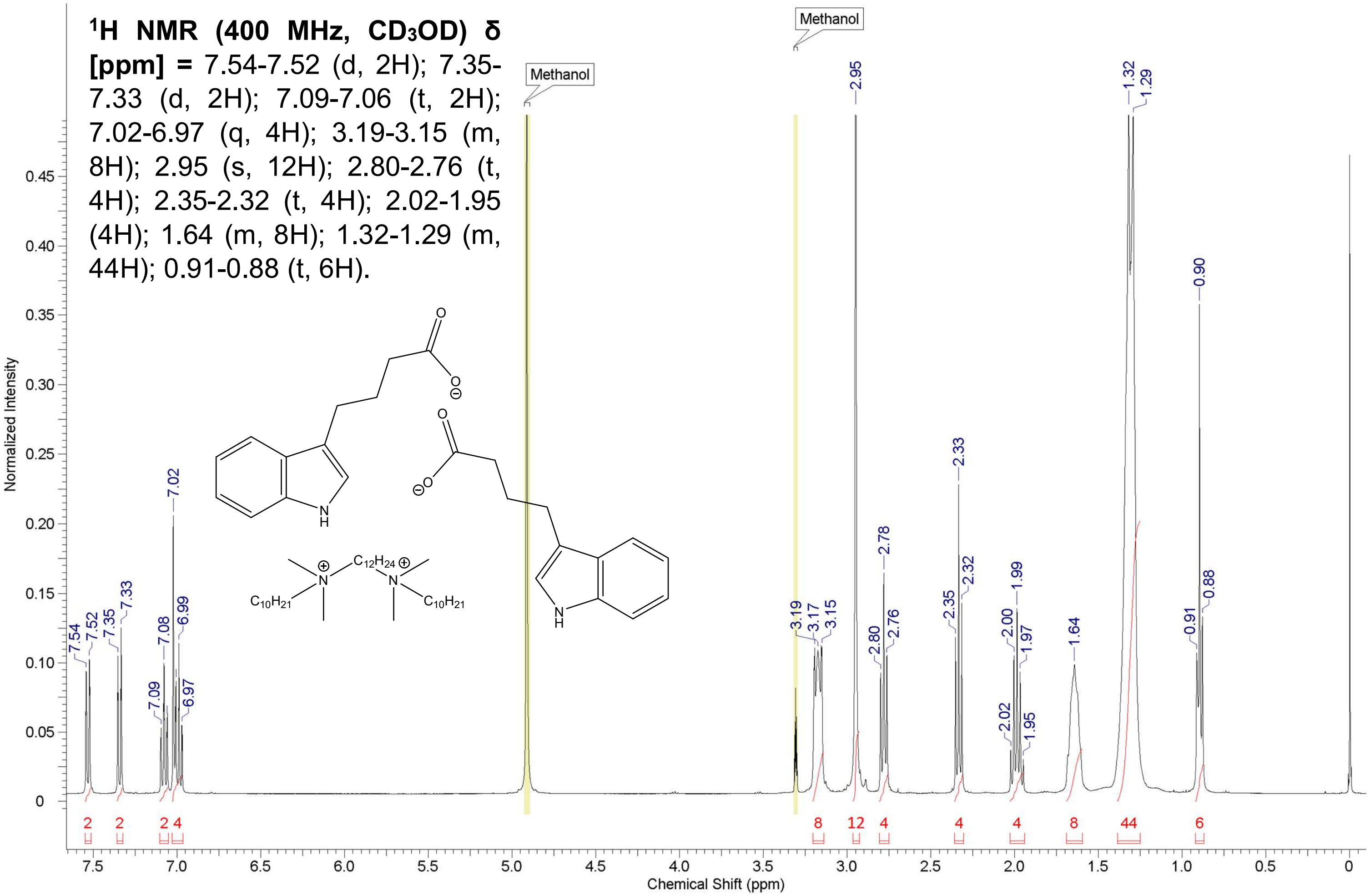

Figure S55. ^{1}H NMR spectrum of indole-3-butyrate dodecylmethylene-1,12-bis(decyldimethylammonium) (**12d**)

File Name						Frequency (MHz)	100.57
Nucleus	13C	Number of Transients	584	Original Points Count	26442	Points Count	32768
Pulse Sequence	s2pul	Receiver Gain	58.00	Solvent	METHANOL-d4	Spectrum Offset (Hz)	10912.5879
Spectrum Type	STANDARD	Sweep Width (Hz)	24038.46	Temperature (degree C)	25.000		

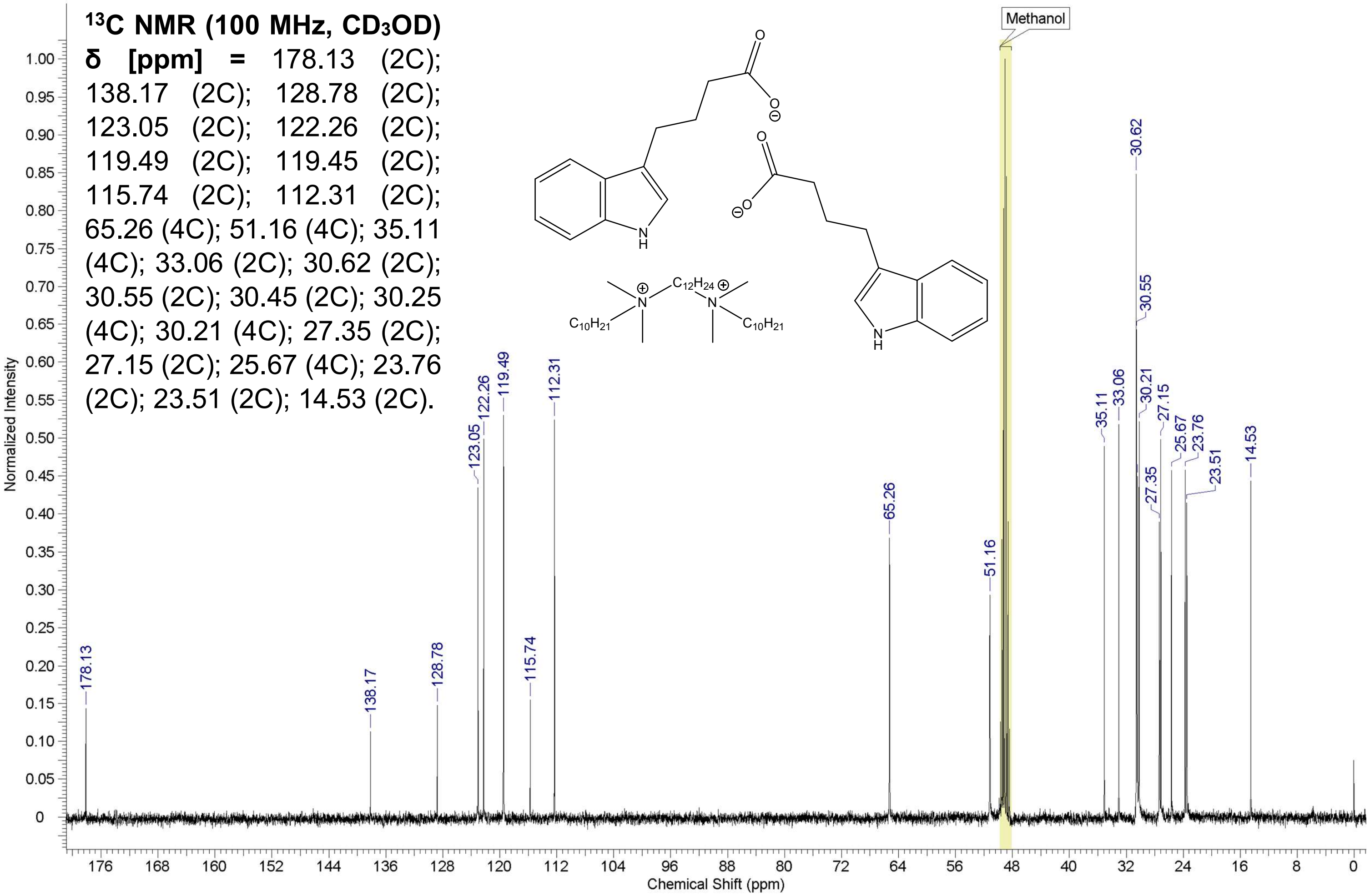

Figure S56. ^{13}C NMR spectrum of indole-3-butyrate dodecylmethylene-1,12-bis(decyldimethylammonium) (**12d**)

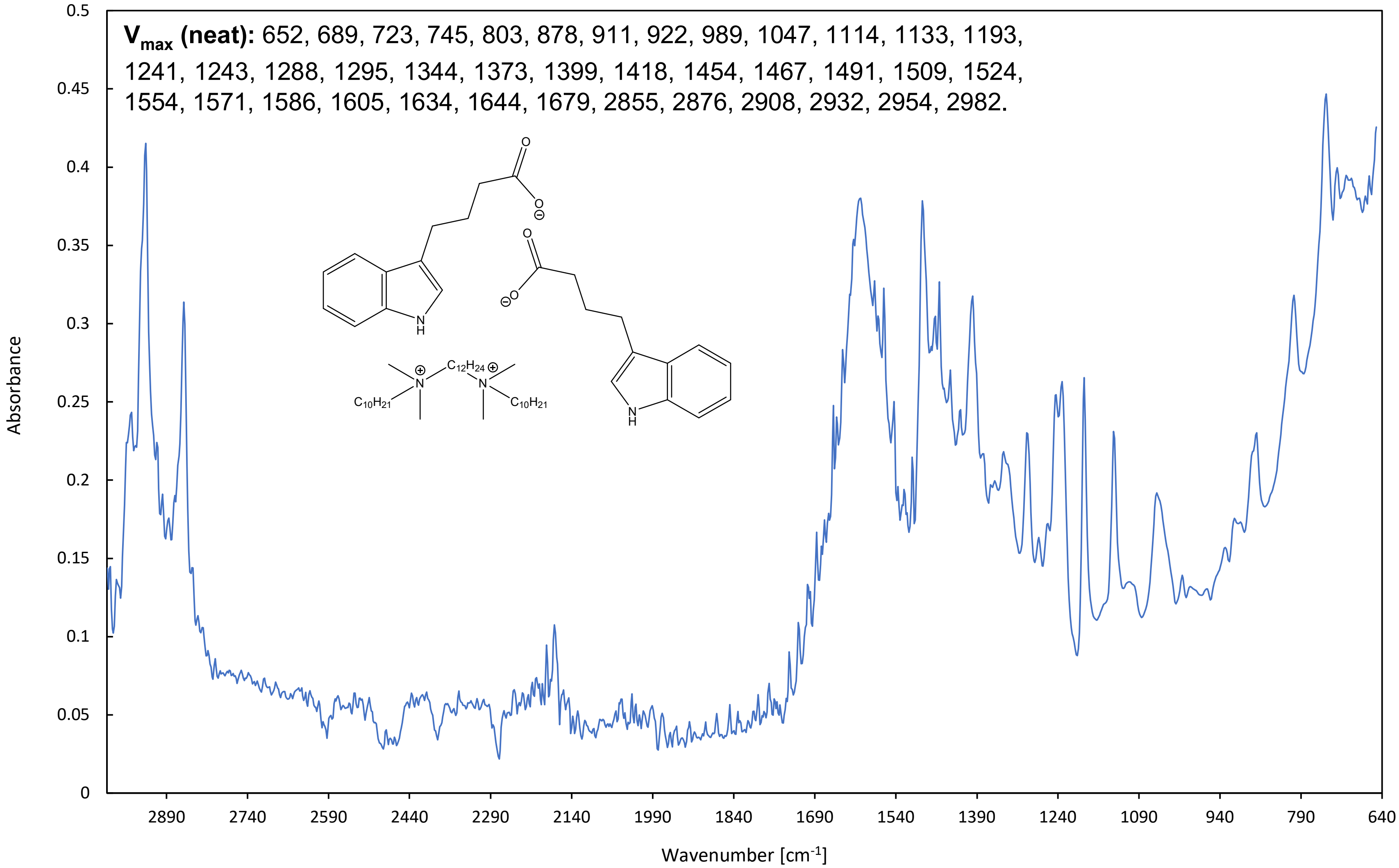

Figure S57. FT-IR spectrum of indole-3-butyrate dodecylmethylene-1,12-bis(decyldimethylammonium) (**12d**)

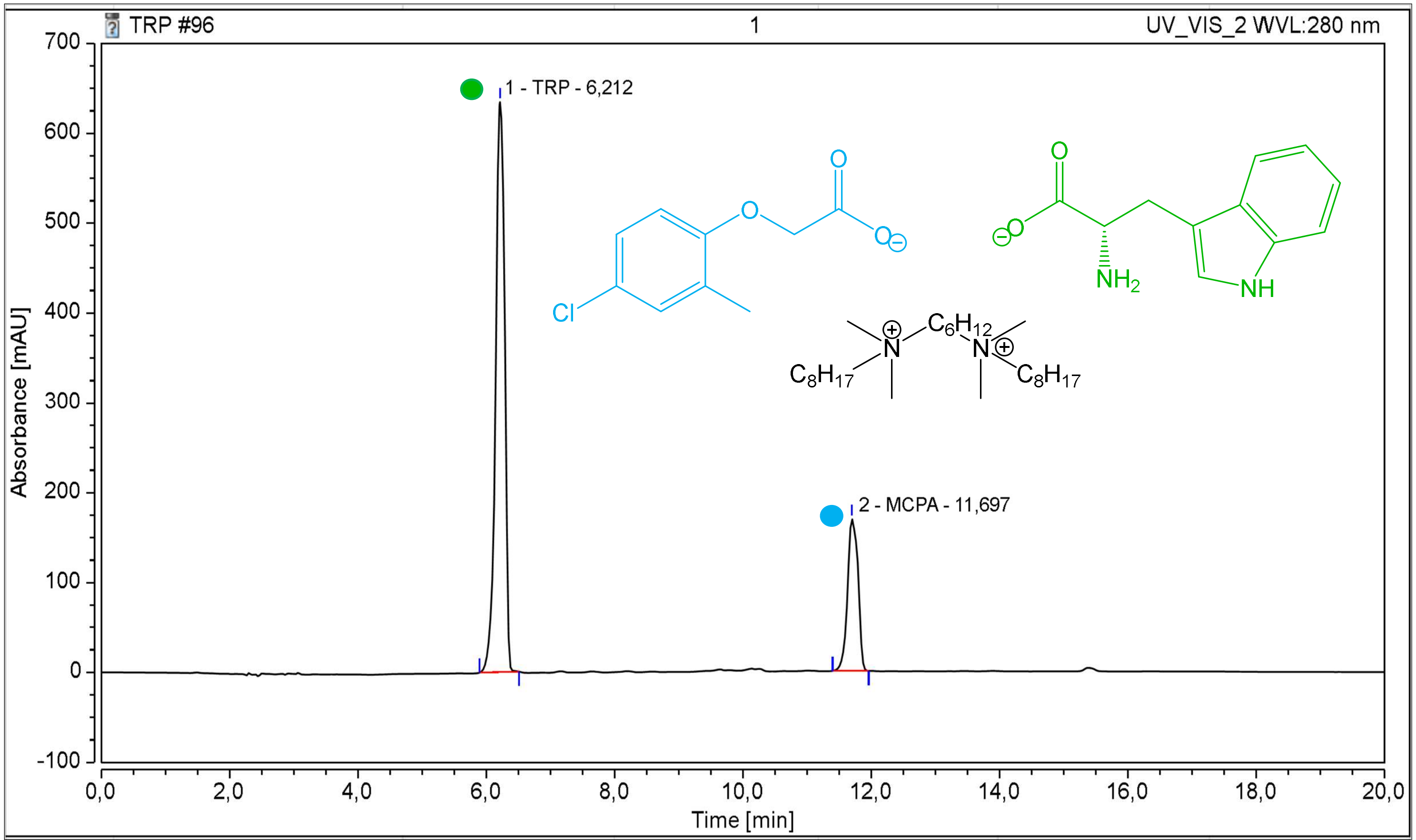

No.	Peak Name	Retention Time [min]	Area [mAU*min]	Height [mAU]	Relative Area [%]	Relative Height [%]
1	TRP	6.212	111.624	634.493	77.64	78.95
2	MCPA	11.697	32.143	169.167	22.36	21.05
**Total:**			**143.767**	**803.660**	**100.00**	**100.00**

Figure S58. HPLC chromatogram of L-tryptophanate 4-chloro-2-methylphenoxyacetate hexamethylene-1,6-bis(octadimethylammonium) (**1**)

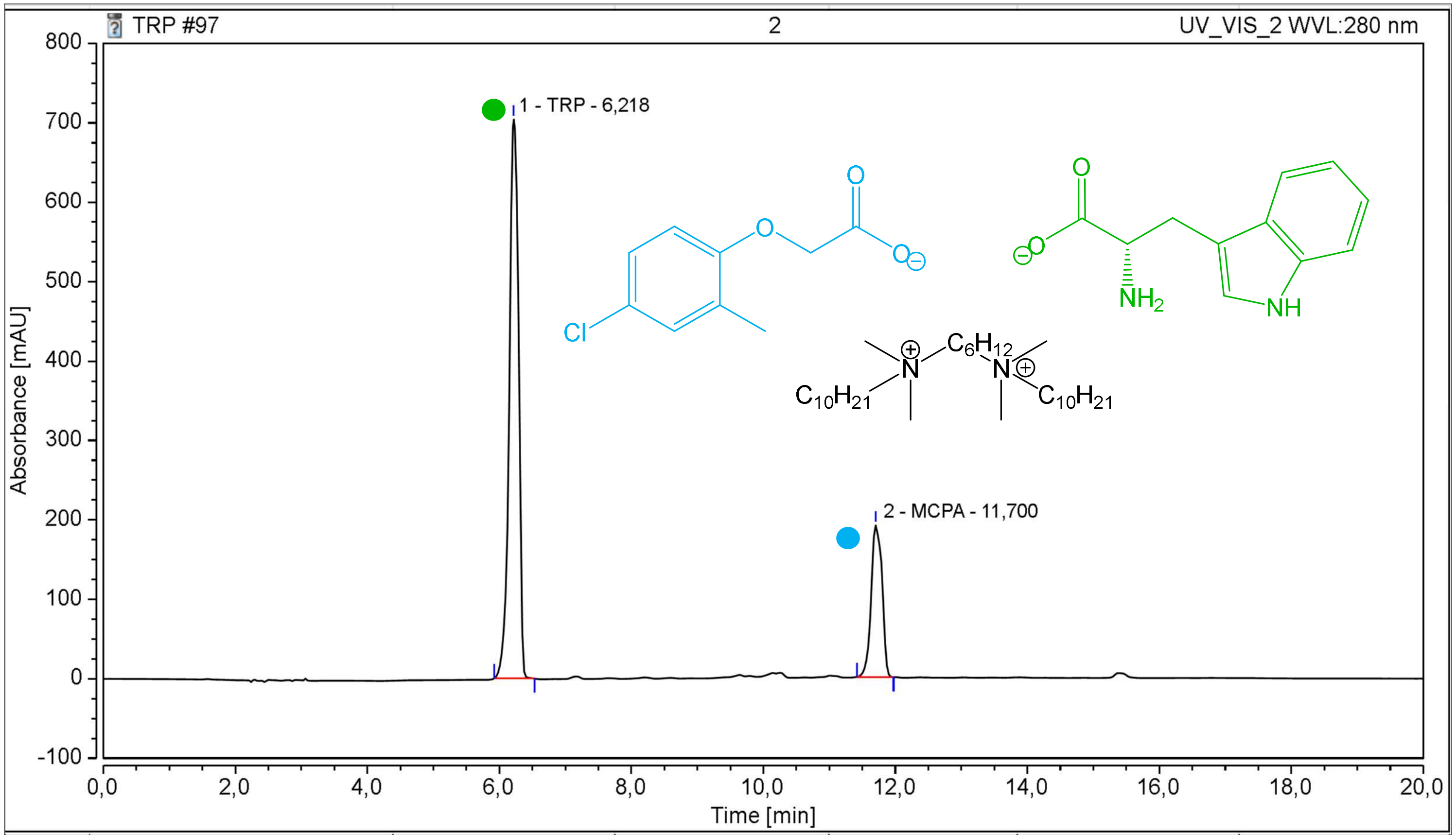

No.	Peak Name	Retention Time [min]	Area [mAU*min]	Height [mAU]	Relative Area [%]	Relative Height [%]
1	TRP	6.218	125.944	704.085	77.46	78.67
2	MCPA	11.700	36.657	190.917	22.54	21.33
**Total:**			**162.601**	**895.002**	**100.00**	**100.00**

Figure S59. HPLC chromatogram of L-tryptophanate 4-chloro-2-methylphenoxyacetate hexamethylene-1,6-bis(decyldimethylammonium) (**2**)

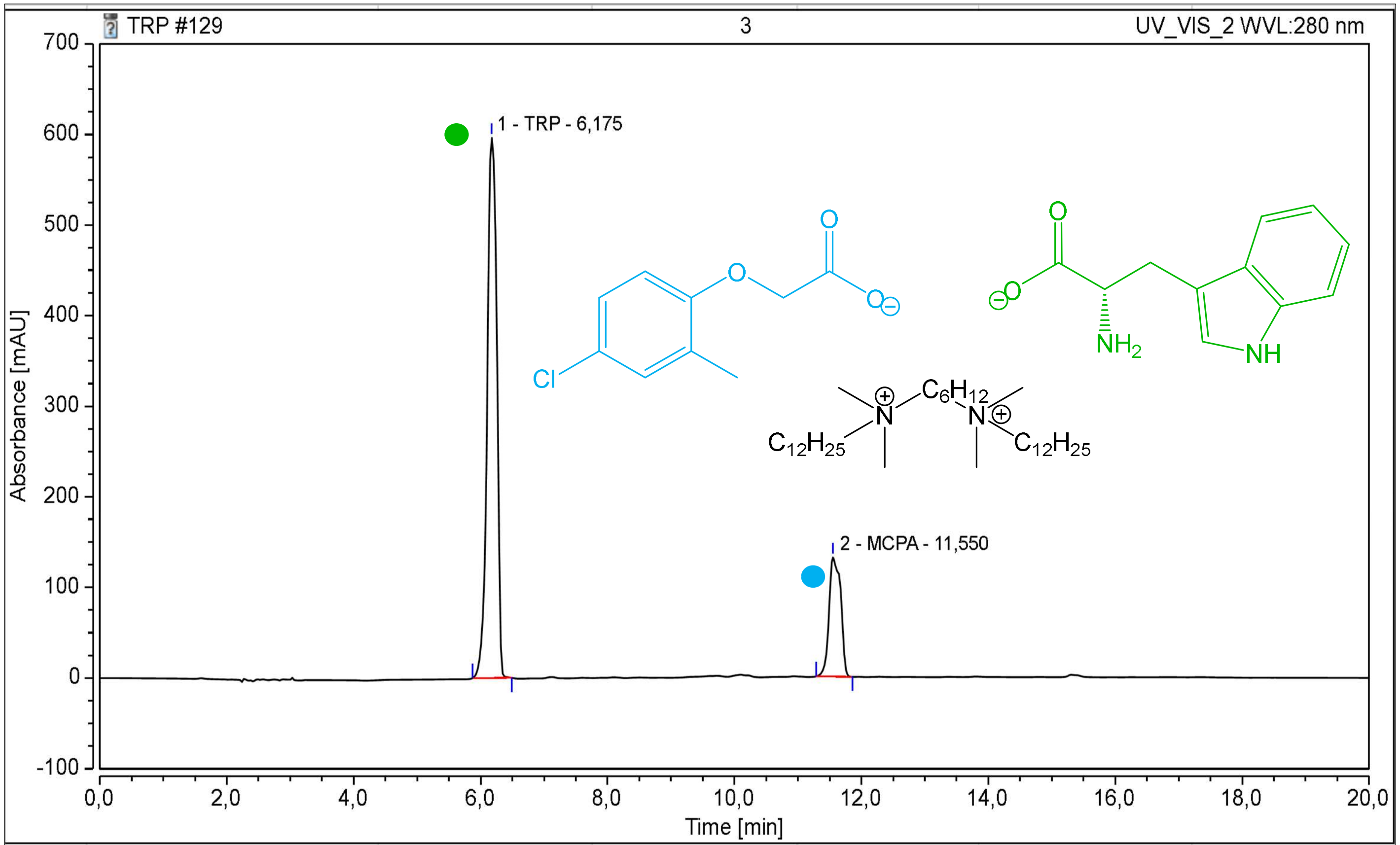

No.	Peak Name	Retention Time [min]	Area [mAU*min]	Height [mAU]	Relative Area [%]	Relative Height [%]
1	TRP	6.175	104.731	596.440	78.90	81.96
2	MCPA	11.550	28.015	131.250	21.10	18.04
**Total:**			**132.746**	**727.690**	**100.00**	**100.00**

Figure S60. HPLC chromatogram of L-tryptophanate 4-chloro-2-methylphenoxyacetate hexamethylene-1,6-bis(dodecyldimethylammonium) (**3**)

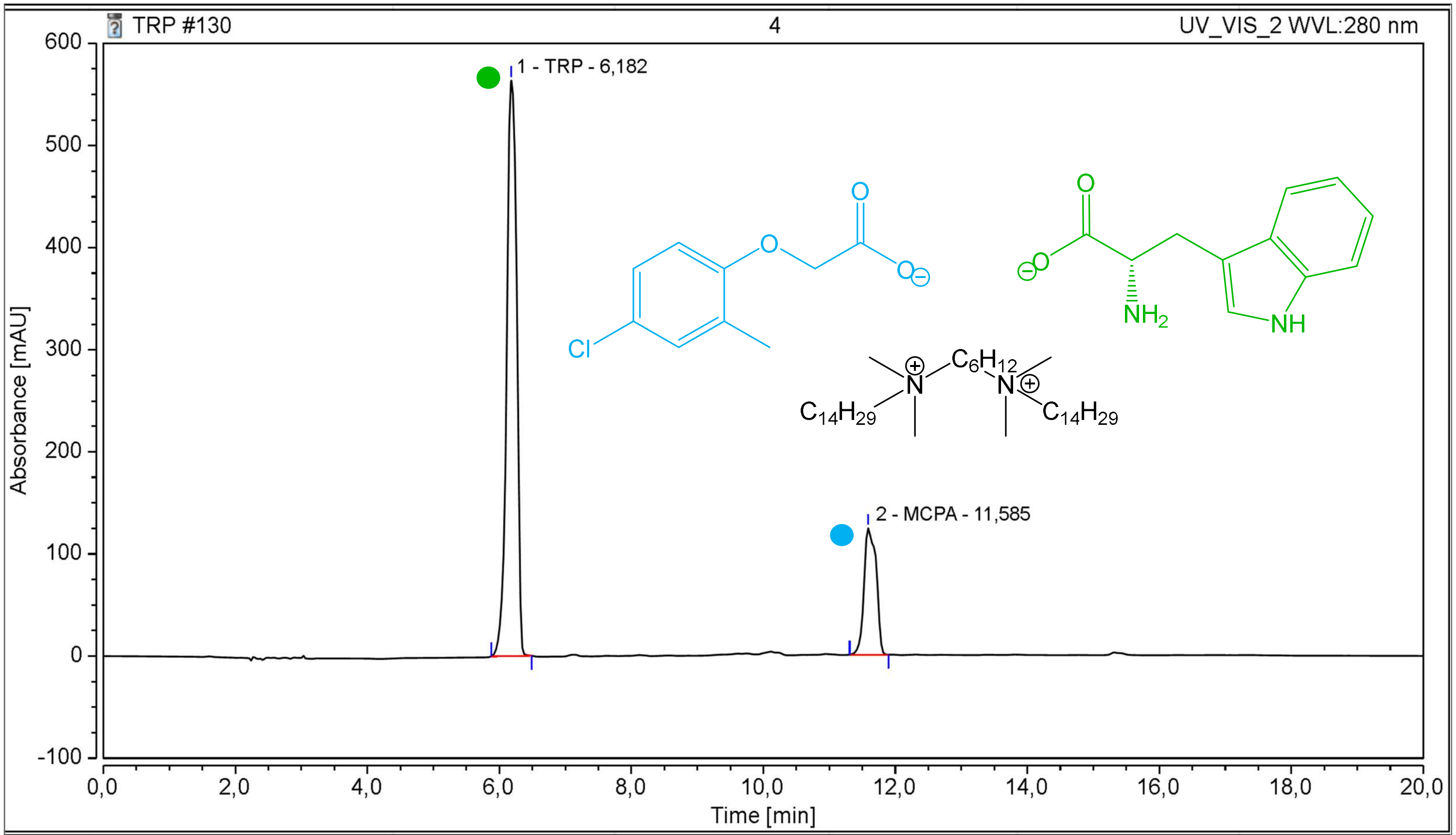

No.	Peak Name	Retention Time [min]	Area [mAU*min]	Height [mAU]	Relative Area [%]	Relative Height [%]
1	TRP	6.182	99.463	563.630	78.97	81.99
2	MCPA	11.585	26.495	123.821	21.03	18.01
**Total:**			**125.958**	**687.451**	**100.00**	**100.00**

Figure S61. HPLC chromatogram of L-tryptophanate 4-chloro-2-methylphenoxyacetate hexamethylene-1,6-bis(tetradecyldimethylammonium) (**4**)

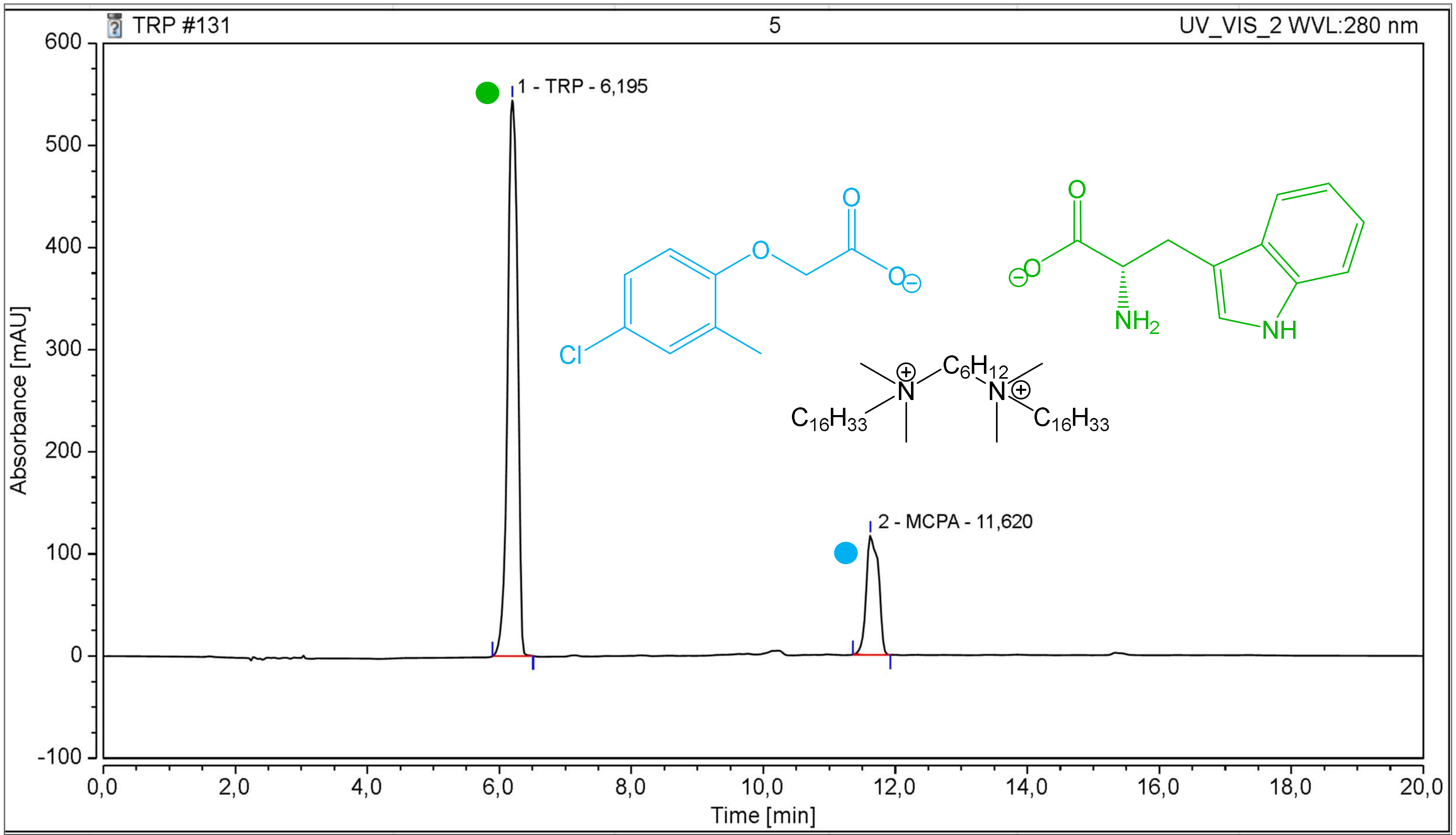

No.	Peak Name	Retention Time [min]	Area [mAU*min]	Height [mAU]	Relative Area [%]	Relative Height [%]
1	TRP	6.195	95.634	544.147	79.22	82.36
2	MCPA	11.620	25.082	116.527	20.78	17.64
**Total:**			**120.716**	**660.674**	**100.00**	**100.00**

Figure S62. HPLC chromatogram of L-tryptophanate 4-chloro-2-methylphenoxyacetate hexamethylene-1,6-bis(hexadecyldimethylammonium) (**5**)

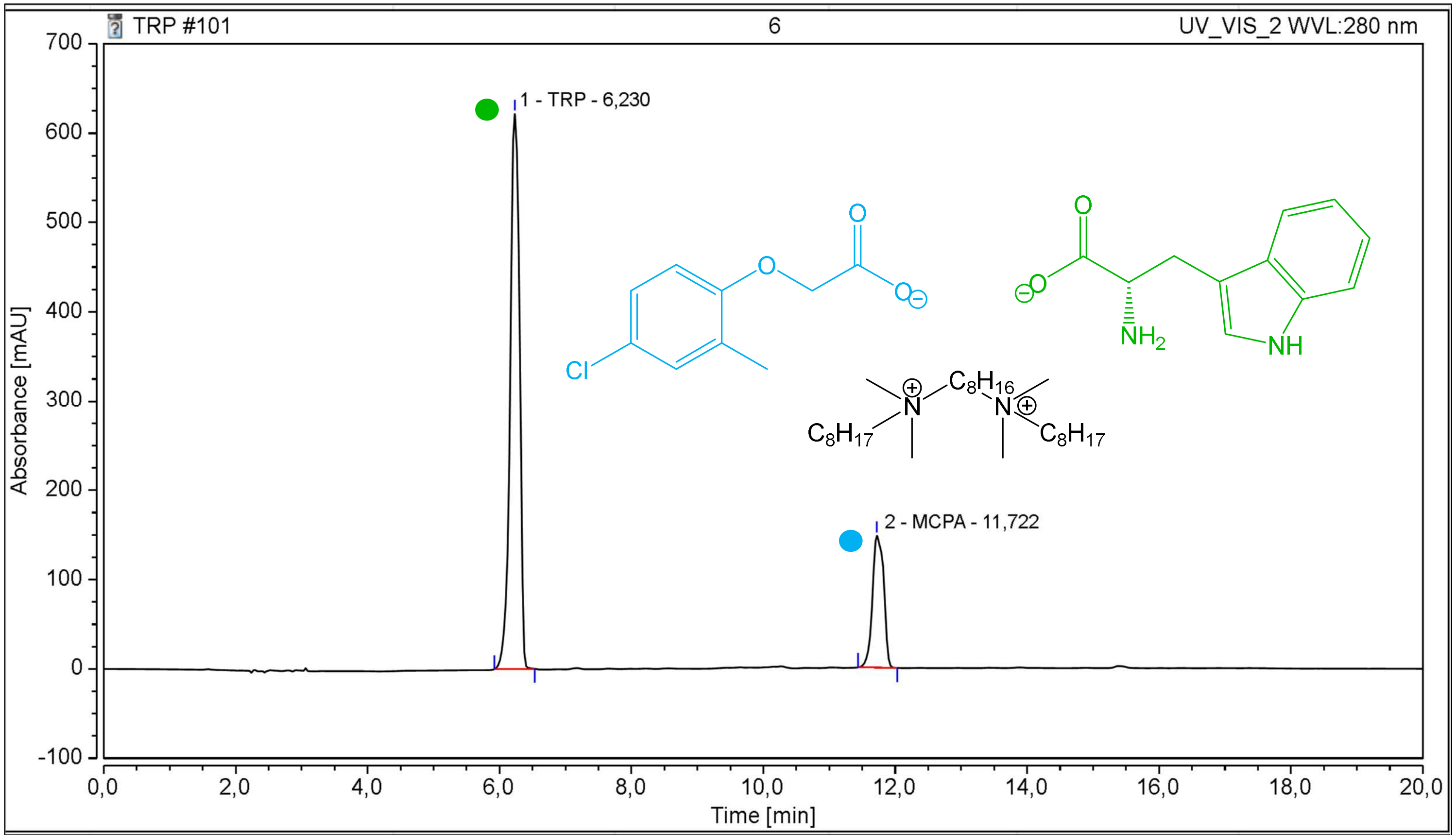

No.	Peak Name	Retention Time [min]	Area [mAU*min]	Height [mAU]	Relative Area [%]	Relative Height [%]
1	TRP	6.230	103.143	621.633	77.88	80.82
2	MCPA	11.722	29.297	147.532	22.12	19.18
**Total:**			**132.440**	**769.165**	**100.00**	**100.00**

Figure S63. HPLC chromatogram of L-tryptophanate 4-chloro-2-methylphenoxyacetate octamethylene-1,8-bis(octadimethylammonium) (**6**)

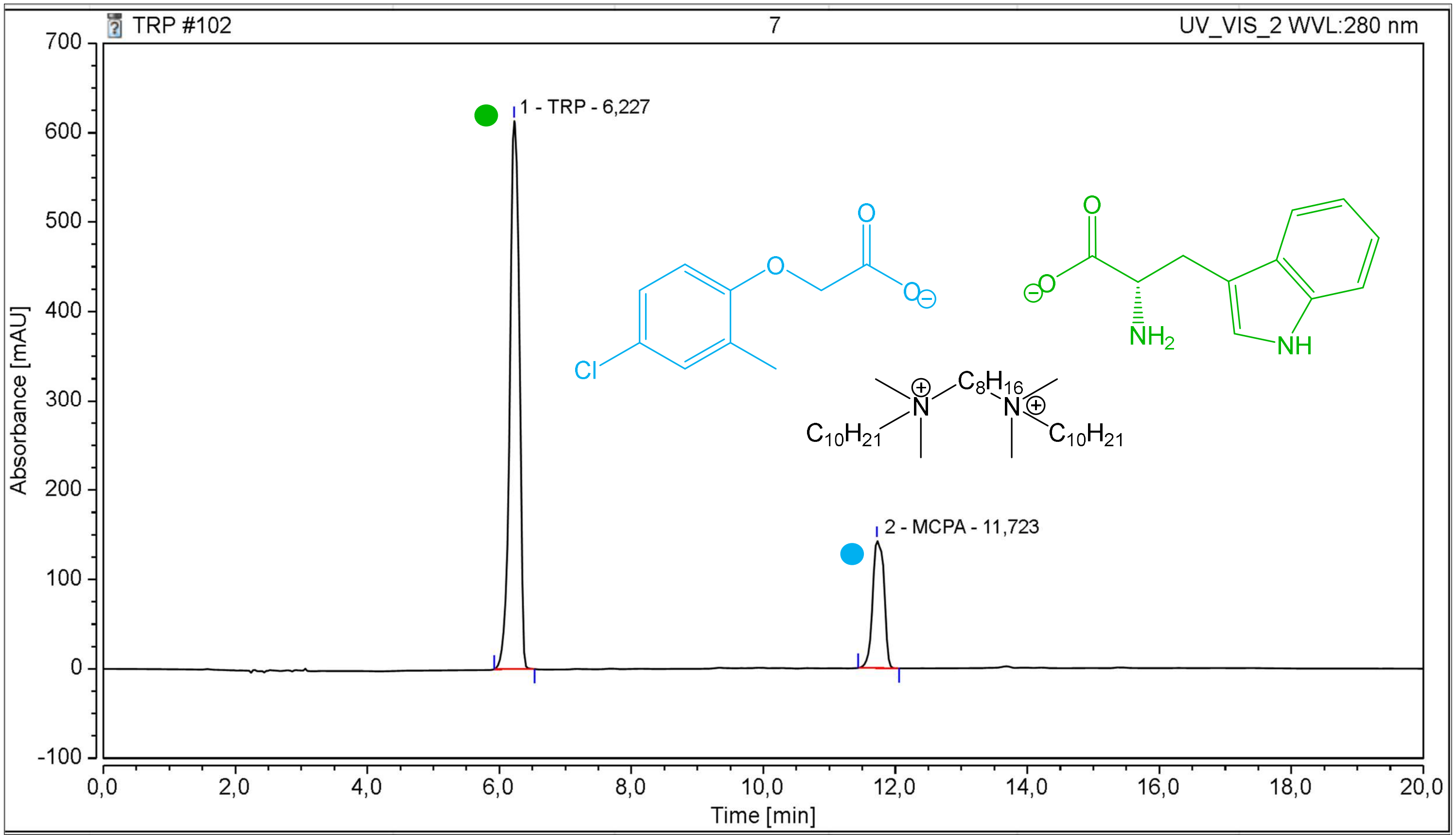

No.	Peak Name	Retention Time [min]	Area [mAU*min]	Height [mAU]	Relative Area [%]	Relative Height [%]
1	TRP	6.227	102.972	613.570	78.25	81.16
2	MCPA	11.723	28.615	142.449	21.75	18.84
**Total:**			**131.587**	**756.019**	**100.00**	**100.00**

Figure S64. HPLC chromatogram of L-t ryptophanate 4-chloro-2-methylphenoxyacetate octamethylene-1,8-bis(decyldimethylammonium) (**7**)

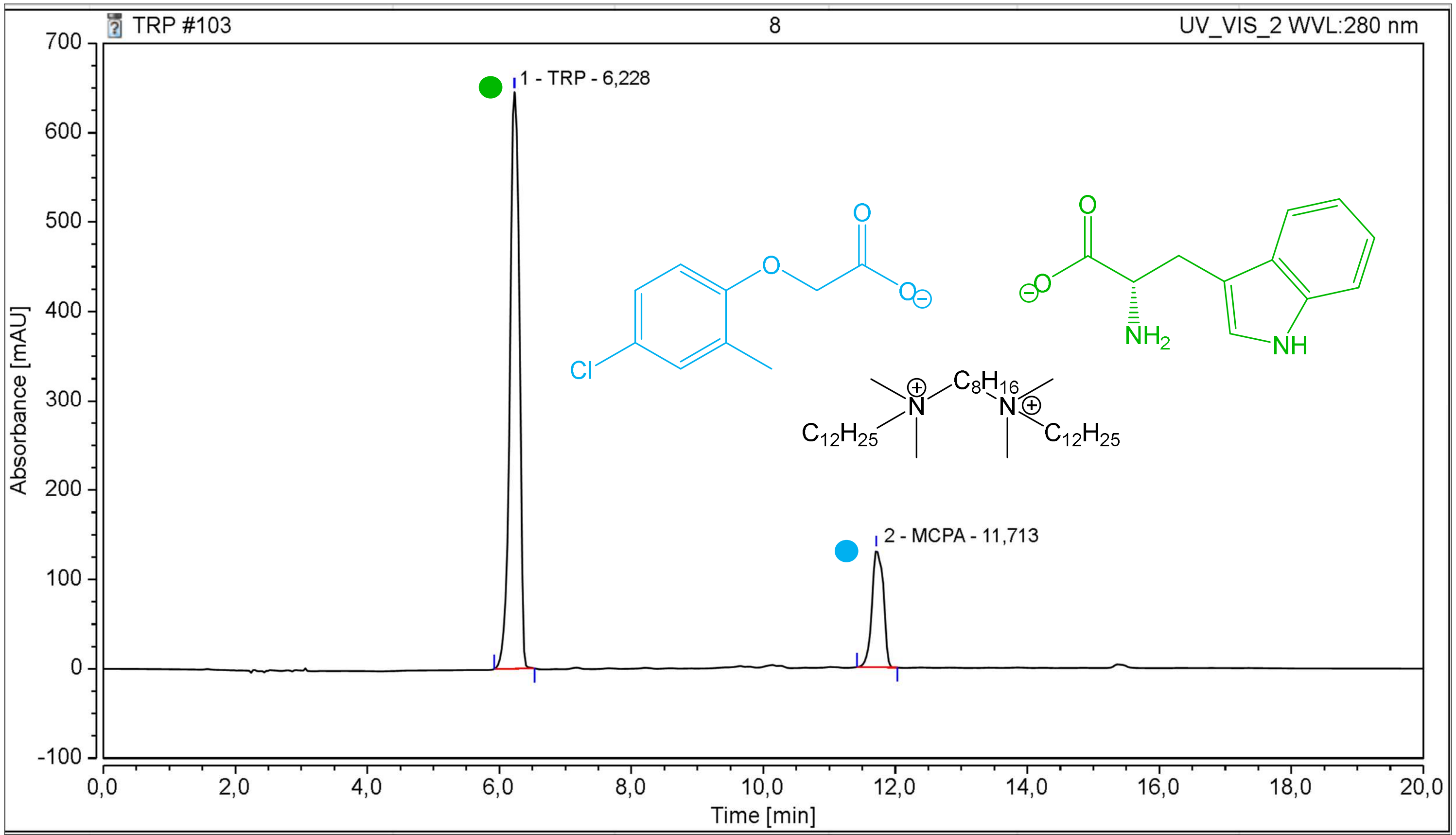

No.	Peak Name	Retention Time [min]	Area [mAU*min]	Height [mAU]	Relative Area [%]	Relative Height [%]
1	TRP	6.228	102.291	645.354	79.50	83.08
2	MCPA	11.713	26.370	131.435	20.50	16.92
**Total:**			**128.661**	**776.789**	**100.00**	**100.00**

Figure S65. HPLC chromatogram of L-tryptophanate 4-chloro-2-methylphenoxyacetate octamethylene-1,8-bis(dodecyldimethylammonium) (**8**)

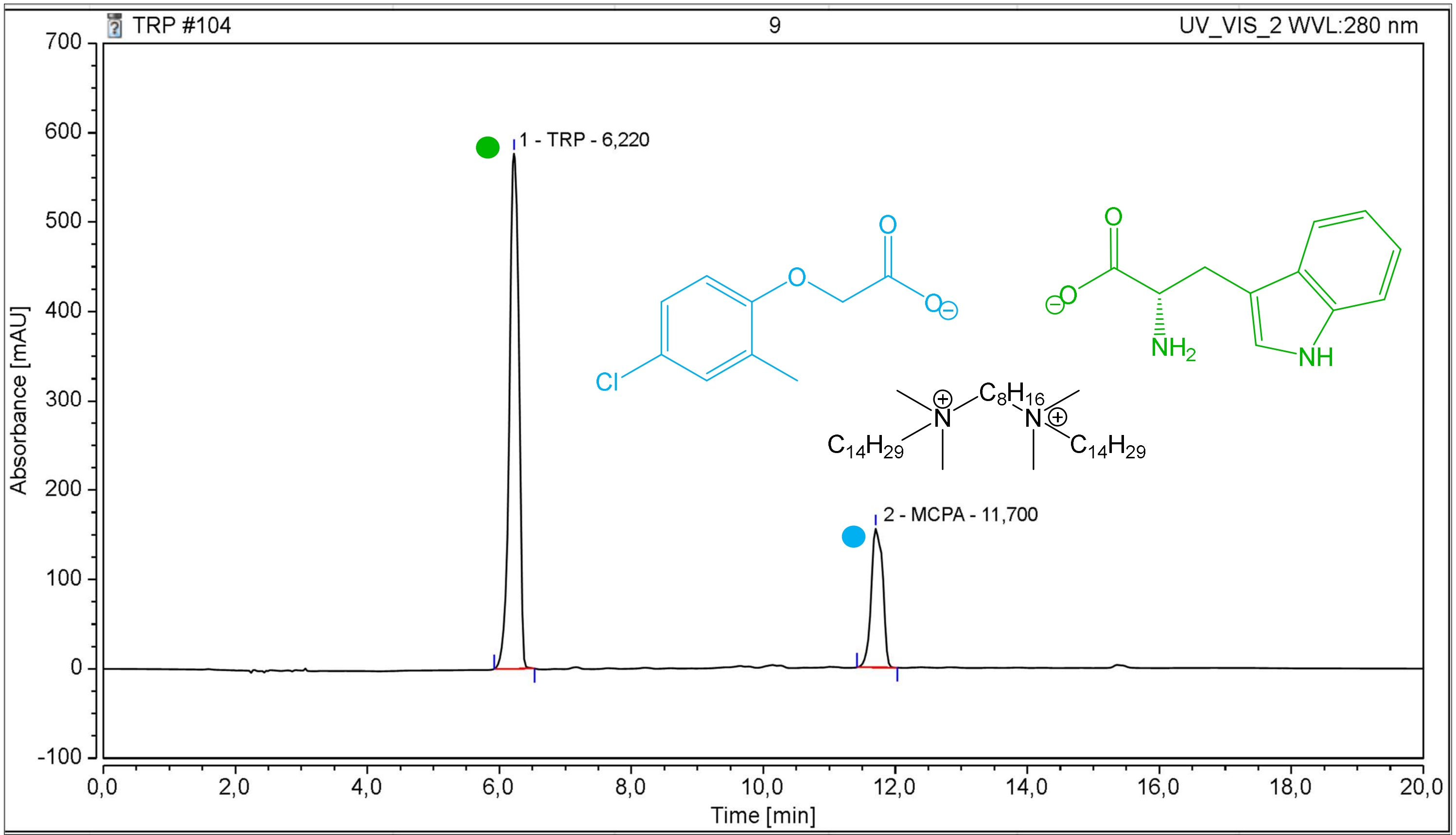

No.	Peak Name	Retention Time [min]	Area [mAU*min]	Height [mAU]	Relative Area [%]	Relative Height [%]
1	TRP	6.220	108.093	576.739	77.46	78.80
2	MCPA	11.700	31.461	155.203	22.54	21.20
**Total:**			**139.554**	**731.942**	**100.00**	**100.00**

Figure S66. HPLC chromatogram of L-tryptophanate 4-chloro-2-methylphenoxyacetate octamethylene-1,8-bis(tetradecyldimethylammonium) (**9**)

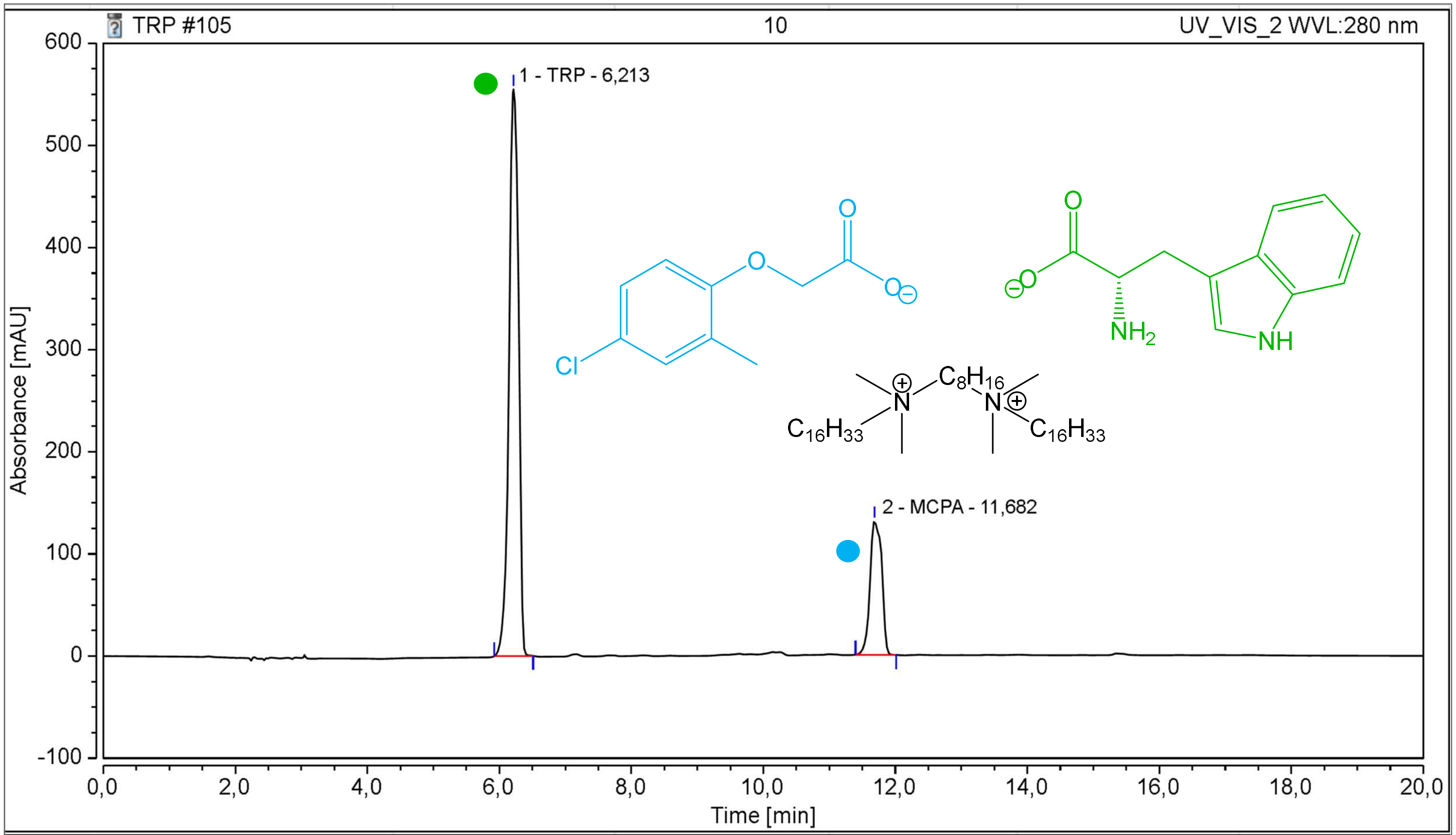

No.	Peak Name	Retention Time [min]	Area [mAU*min]	Height [mAU]	Relative Area [%]	Relative Height [%]
1	TRP	6.213	93.505	555.076	77.57	80.92
2	MCPA	11.682	27.038	130.908	22.43	19.08
**Total:**			**120.543**	**685.984**	**100.00**	**100.00**

Figure S67. HPLC chromatogram of L-tryptophanate 4-chloro-2-methylphenoxyacetate octamethylene-1,8-bis(hexadecyldimethylammonium) (**10**)

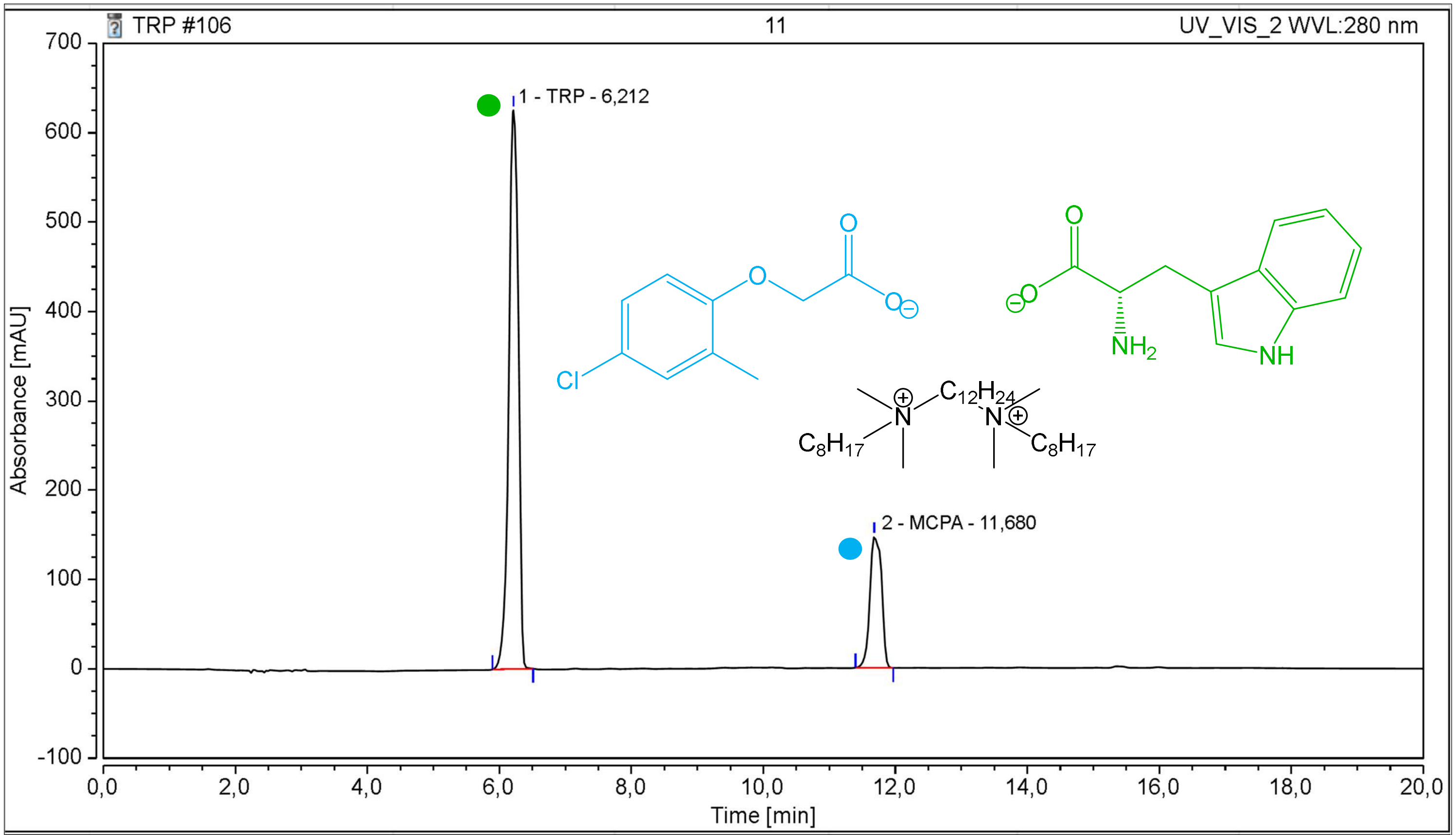

No.	Peak Name	Retention Time [min]	Area [mAU*min]	Height [mAU]	Relative Area [%]	Relative Height [%]
1	TRP	6.212	105.372	625.420	77.71	81.01
2	MCPA	11.680	30.225	146.638	22.29	18.99
**Total:**			**135.597**	**772.058**	**100.00**	**100.00**

Figure S68. HPLC chromatogram of L-tryptophanate 4-chloro-2-methylphenoxyacetate dodecylmethylene-1,12-bis(octadimethylammonium) (**11**)

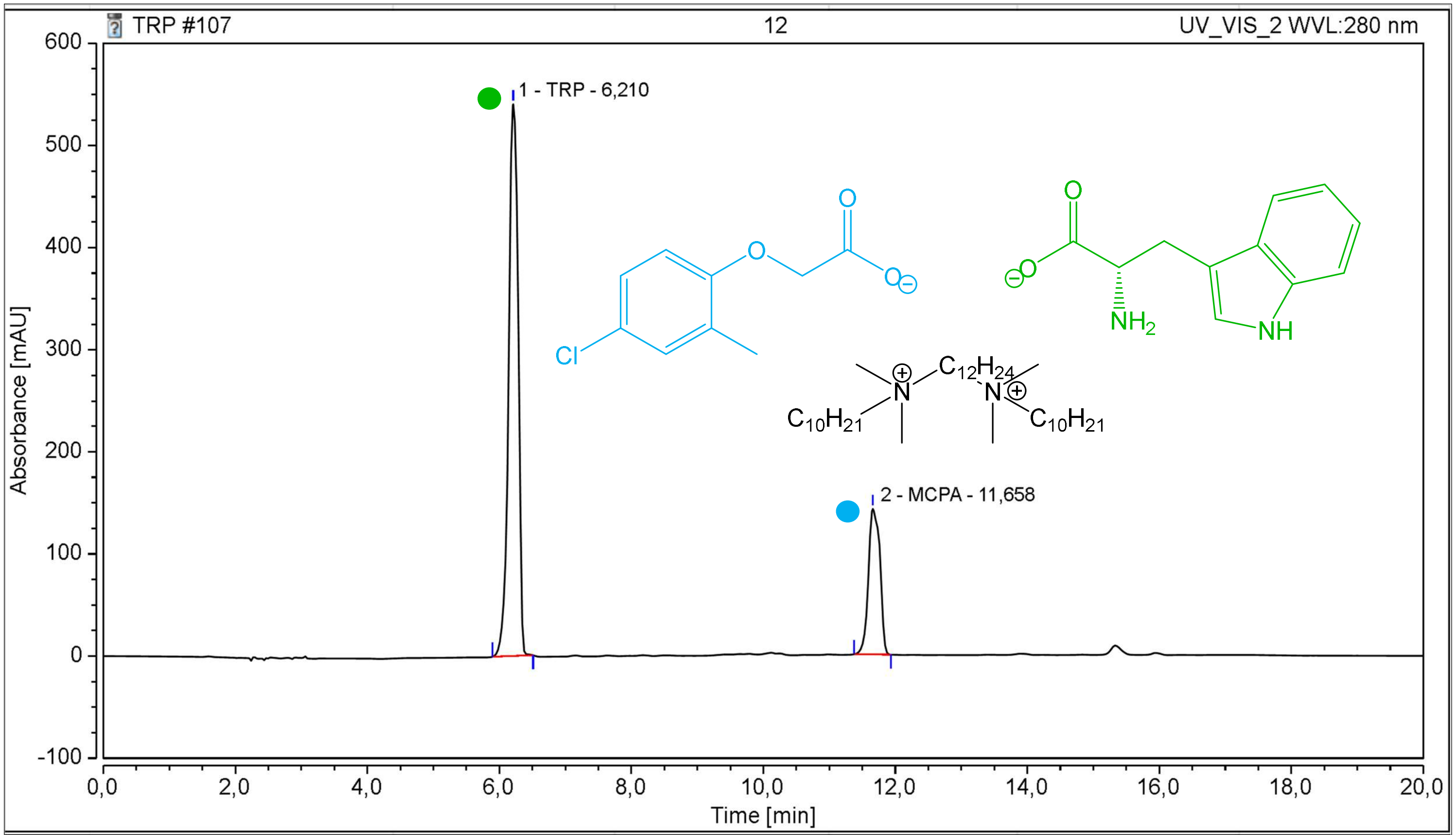

No.	Peak Name	Retention Time [min]	Area [mAU*min]	Height [mAU]	Relative Area [%]	Relative Height [%]
1	TRP	6.210	99.387	540.387	77.44	79.13
2	MCPA	11.658	28.953	142.525	22.56	20.87
**Total:**			**128.340**	**682.912**	**100.00**	**100.00**

Figure S69. HPLC chromatogram of L-tryptophanate 4-chloro-2-methylphenoxyacetate dodecylmethylene-1,12-bis(decyldimethylammonium) (**12**)

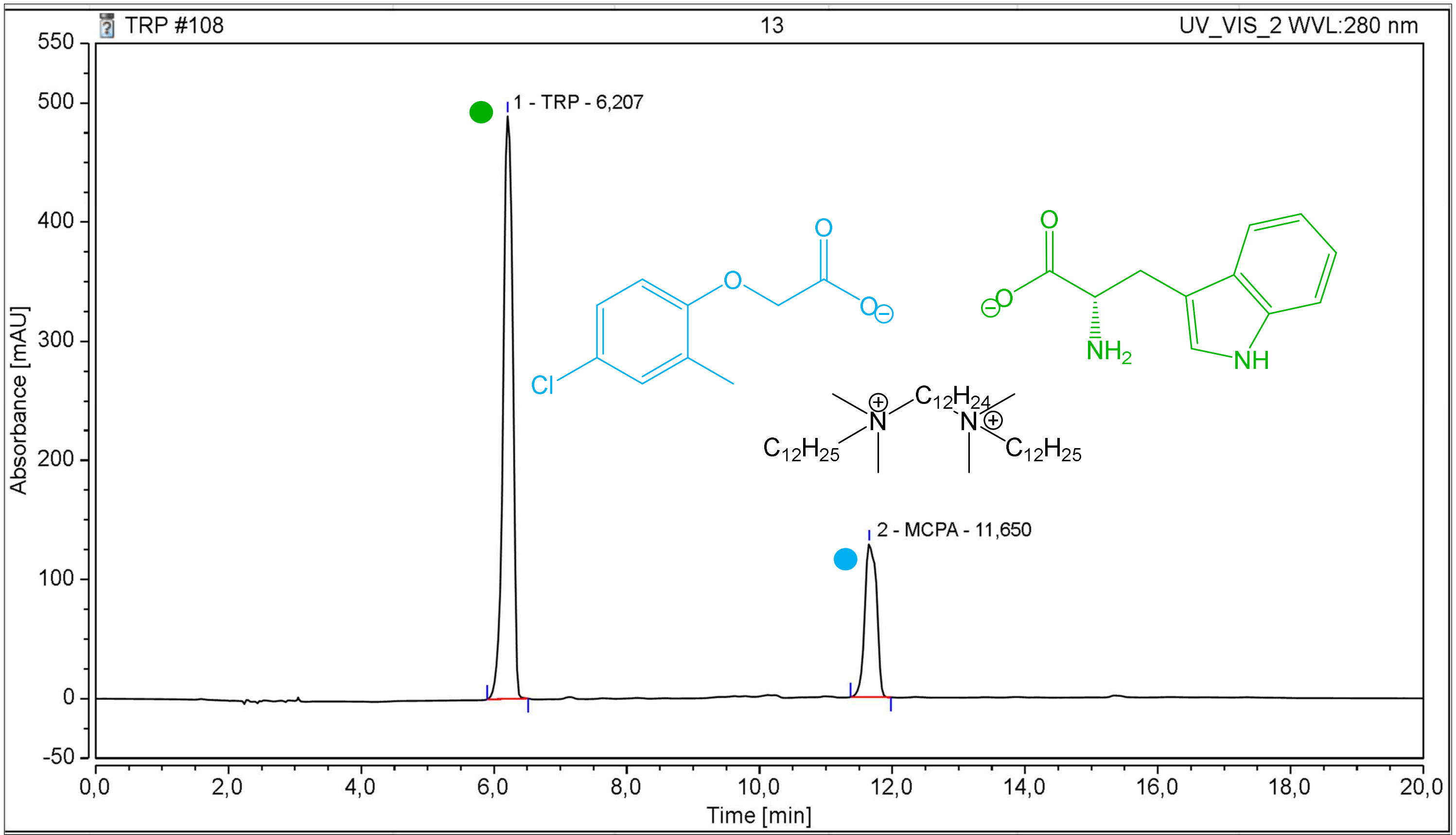

No.	Peak Name	Retention Time [min]	Area [mAU*min]	Height [mAU]	Relative Area [%]	Relative Height [%]
1	TRP	6.207	91.949	489.187	77.53	79.20
2	MCPA	11.650	26.641	128.483	22.47	20.80
**Total:**			**118.590**	**617.670**	**100.00**	**100.00**

Figure S70. HPLC chromatogram of L-tryptophanate 4-chloro-2-methylphenoxyacetate dodecylmethylene-1,12-bis(dodecyldimethylammonium) (**13**)

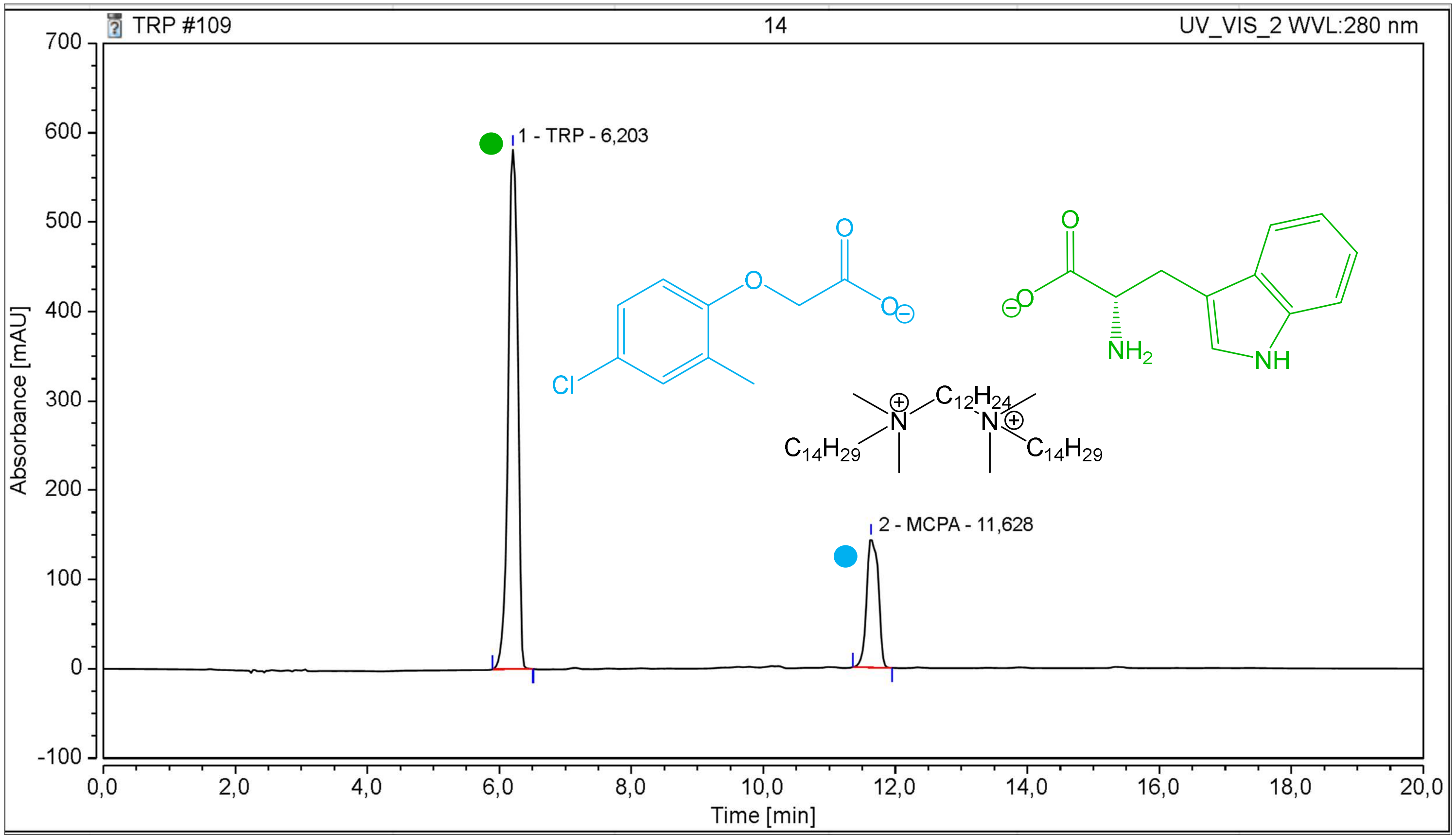

No.	Peak Name	Retention Time [min]	Area [mAU*min]	Height [mAU]	Relative Area [%]	Relative Height [%]
1	TRP	6.203	101.858	581.591	77.45	80.10
2	MCPA	11.628	29.647	144.510	22.55	19.90
**Total:**			**131.505**	**726.101**	**100.00**	**100.00**

Figure S71. HPLC chromatogram of L-tryptophanate 4-chloro-2-methylphenoxyacetate dodecylmethylene-1,12-bis(tetradecyldimethylammonium) (**14**)

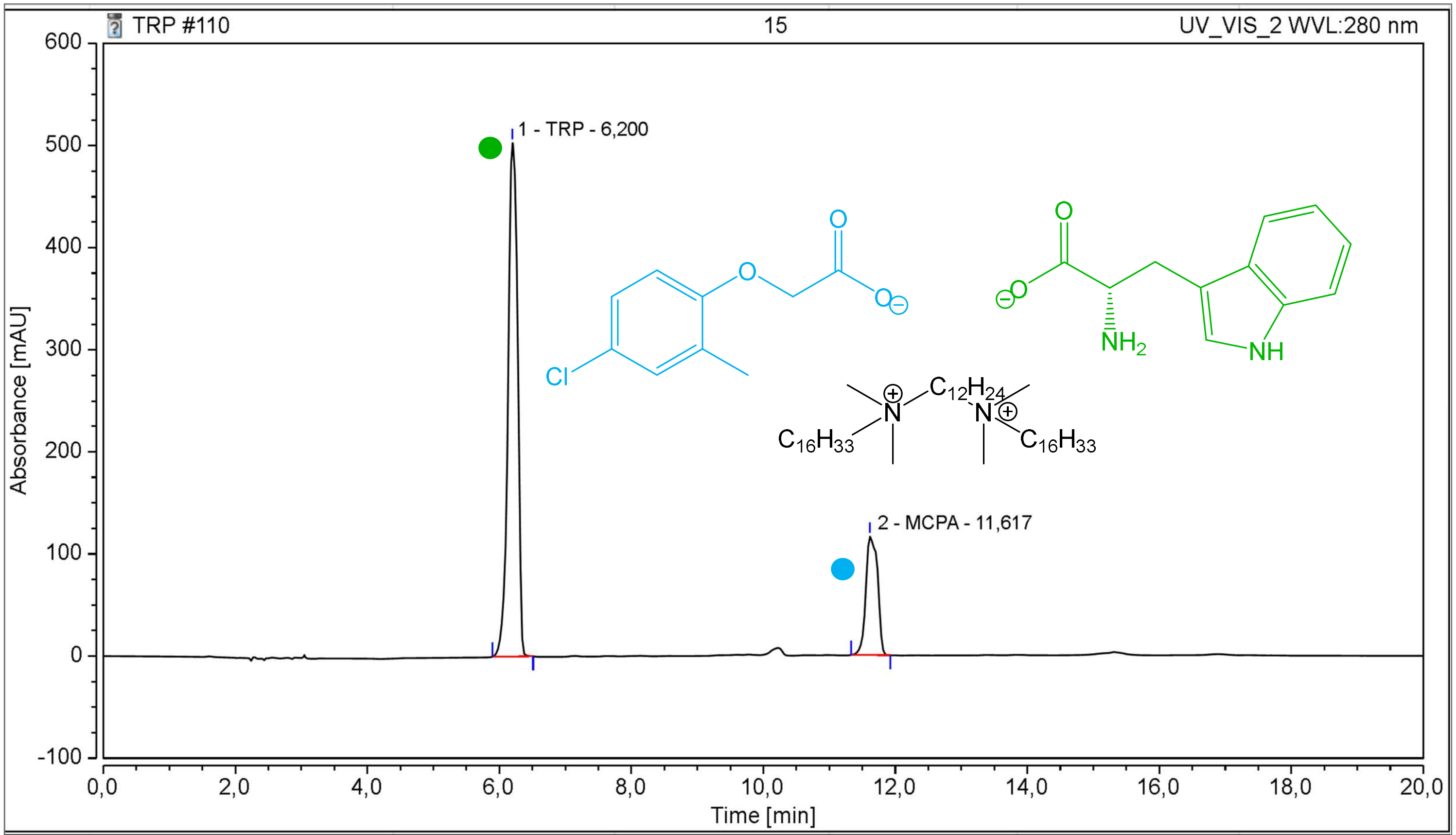

No.	Peak Name	Retention Time [min]	Area [mAU*min]	Height [mAU]	Relative Area [%]	Relative Height [%]
1	TRP	6.200	84.889	502.993	77.95	81.26
2	MCPA	11.617	24.007	115.987	22.05	18.74
**Total:**			**108.896**	**618.980**	**100.00**	**100.00**

Figure S72. HPLC chromatogram of L-tryptophanate 4-chloro-2-methylphenoxyacetate dodecylmethylene-1,12-bis(hexadecyldimethylammonium) (**15**)

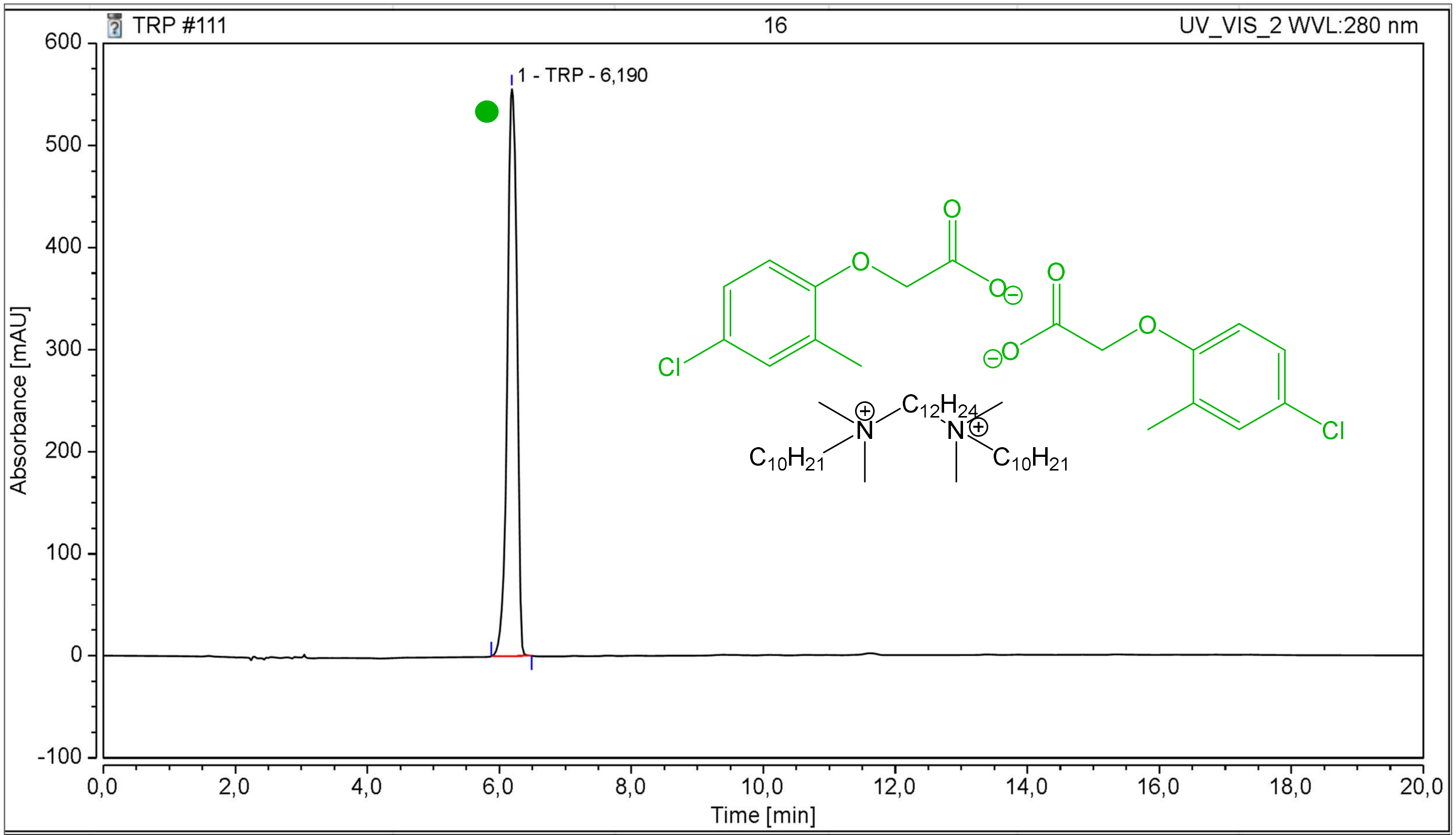

No.	Peak Name	Retention Time [min]	Area [mAU*min]	Height [mAU]	Relative Area [%]	Relative Height [%]
1	TRP	6.190	94.099	555.624	100.00	100.00
n.a.	MCPA	n.a.	n.a.	n.a.	n.a.	n.a.
**Total:**			**94.099**	**555.624**	**100.00**	**100.00**

Figure S73. HPLC chromatogram of 4-chloro-2-methylphenoxyacetate dodecylmethylene-1,12-bis(hexadecyldimethylammonium) (**12a**)

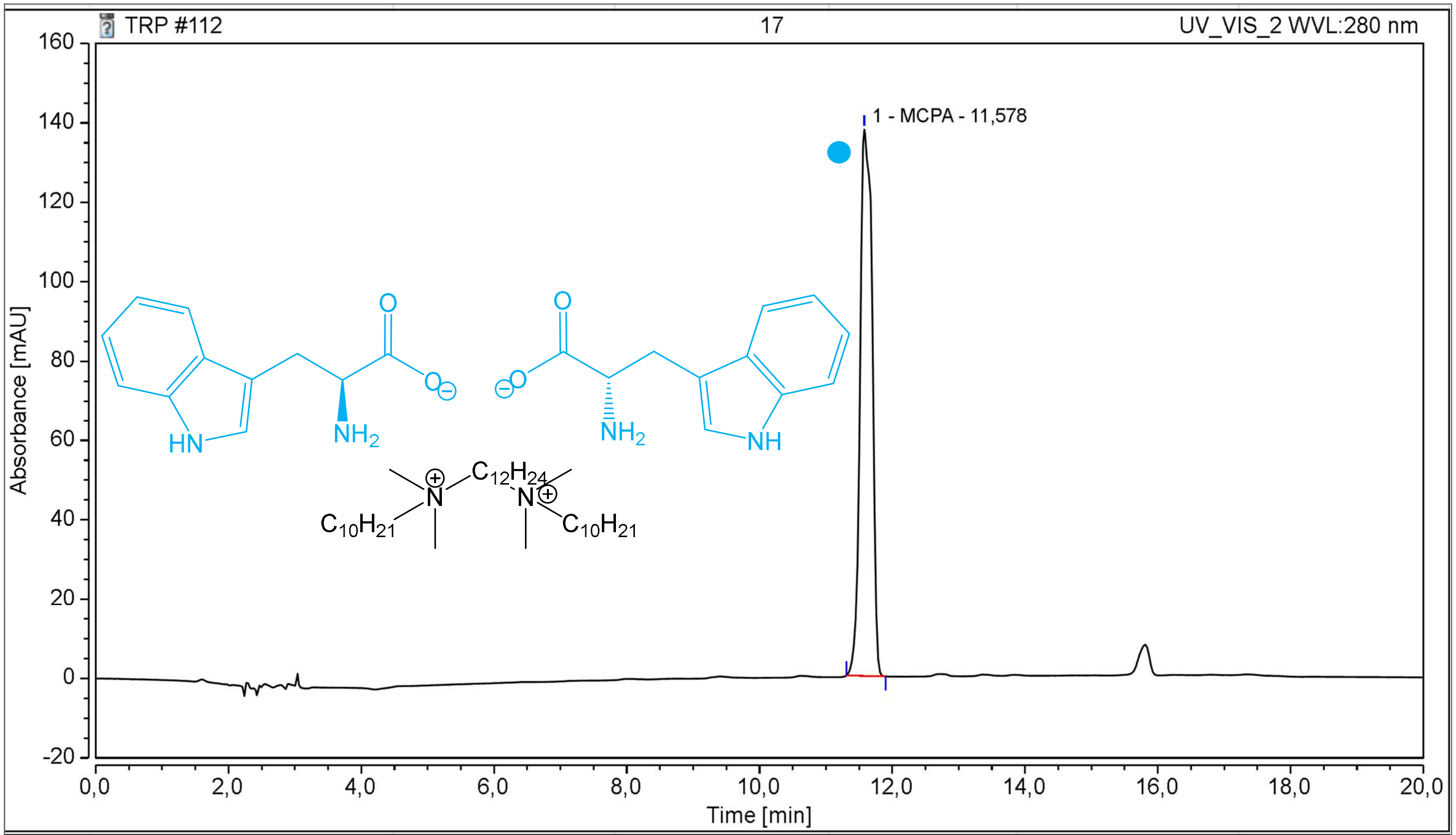

No.	Peak Name	Retention Time [min]	Area [mAU*min]	Height [mAU]	Relative Area [%]	Relative Height [%]
n.a.	TRP	n.a.	n.a.	n.a.	n.a.	n.a.
1	MCPA	11.578	28.916	137.738	100.00	100.00
**Total:**			**28.916**	**137.738**	**100.00**	**100.00**

Figure S74. HPLC chromatogram of L-tryptophanate dodecylmethylene-1,12-bis(hexadecyldimethylammonium) (**12b**)

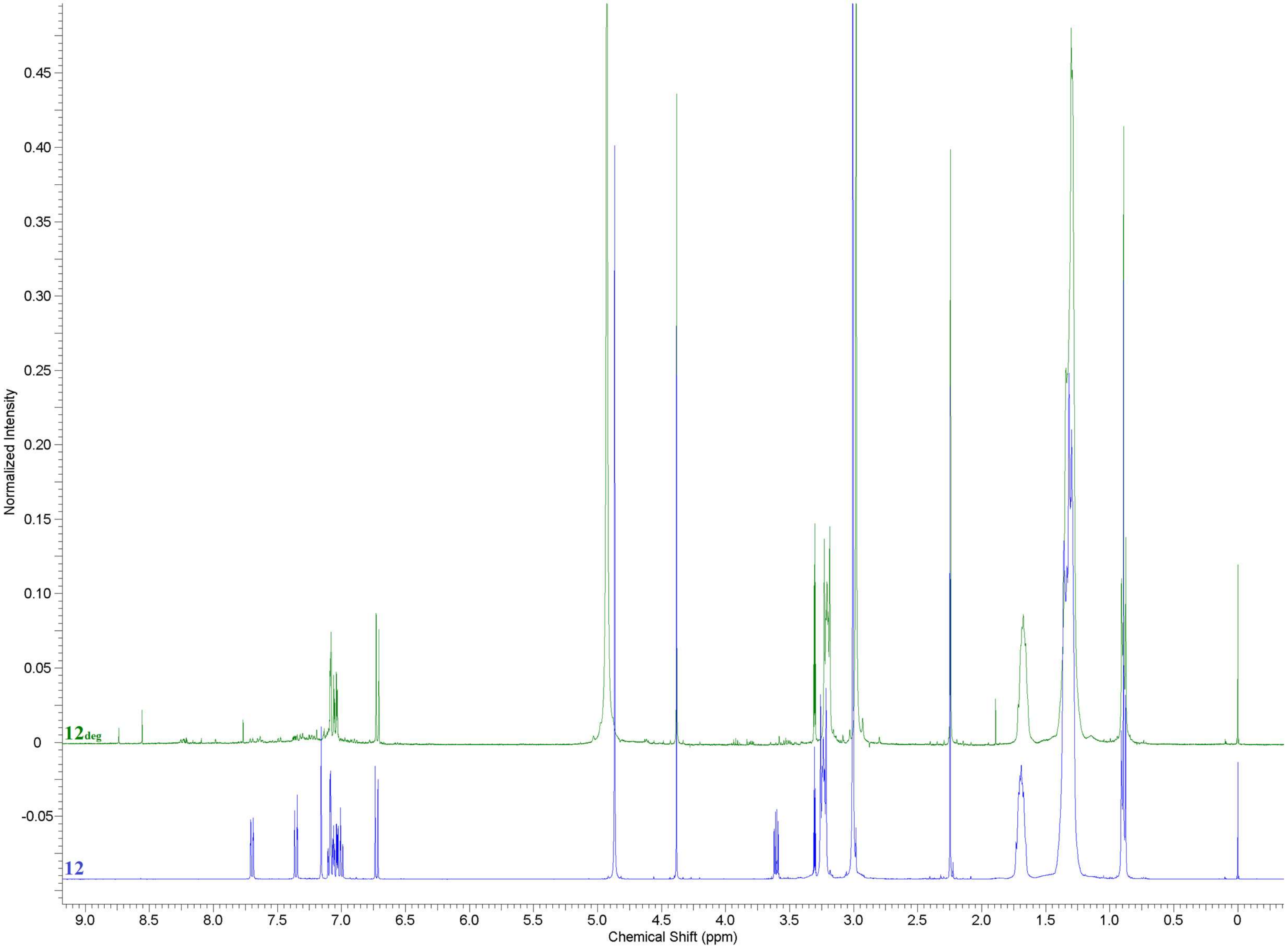

Figure S75. Comparison of the spectra ^1H NMR of DSIL **12** and its degradation products (**12_{deg}**)

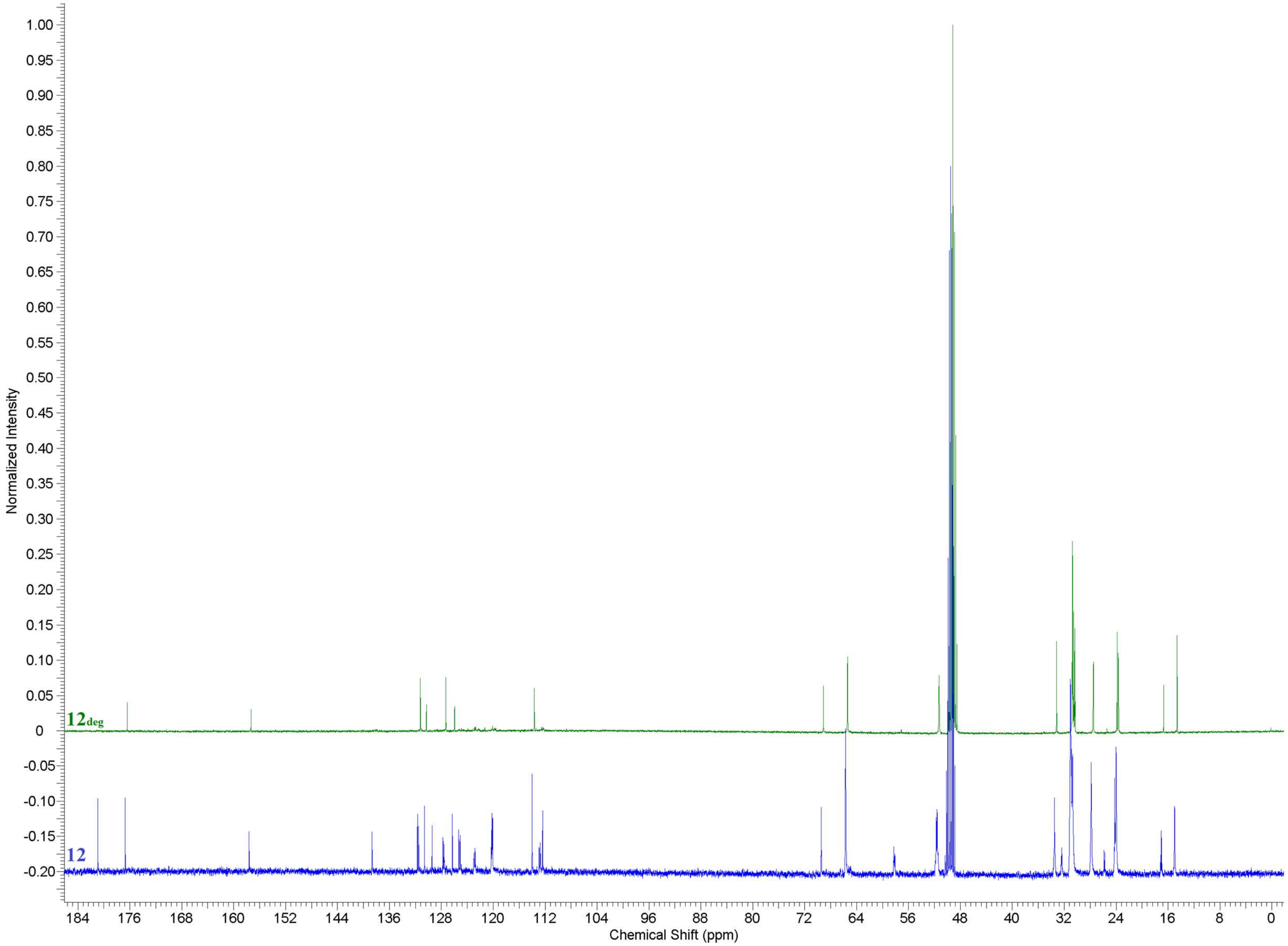

Figure S76. Comparison of the spectra ^{13}C NMR of DSIL **12** and its degradation products (**12$_{deg}$**)

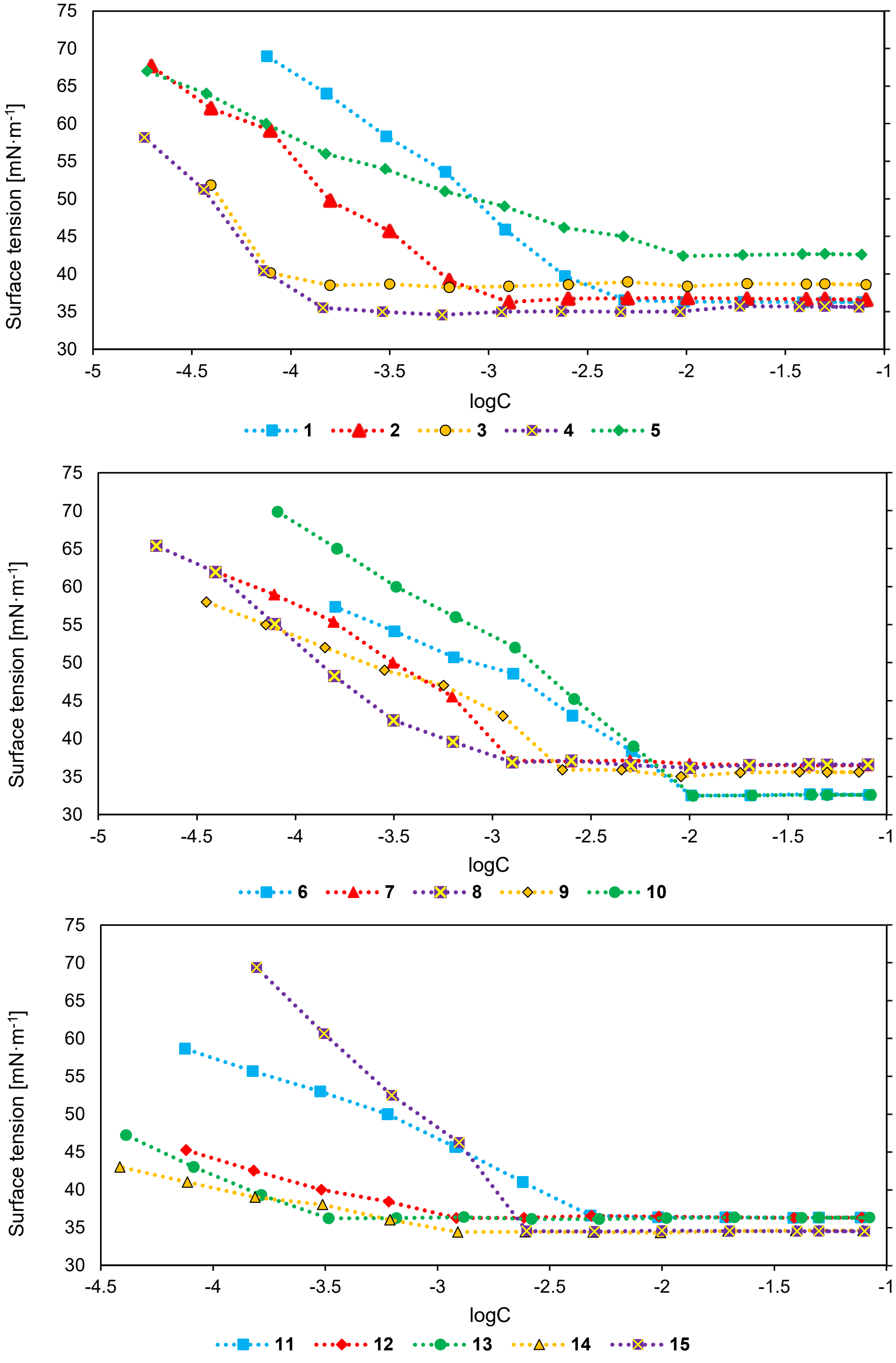

Figure S77. The surface tension isotherms for analyzed DSILs at 25 °C
